# Repetitive DNA sequence detection and its role in the human genome

**DOI:** 10.1038/s42003-023-05322-y

**Published:** 2023-09-19

**Authors:** Xingyu Liao, Wufei Zhu, Juexiao Zhou, Haoyang Li, Xiaopeng Xu, Bin Zhang, Xin Gao

**Affiliations:** 1 Computational Bioscience Research Center (CBRC), Computer, Electrical and Mathematical Sciences and Engineering Division, King Abdullah University of Science and Technology (KAUST), Thuwal, 23955 Saudi Arabia; 2 Department of Endocrinology, Yichang Central People’s Hospital, The First College of Clinical Medical Science, China Three Gorges University, 443000 Yichang, P.R. China

**Keywords:** Data mining, Machine learning, High-throughput screening

## Abstract

Repetitive DNA sequences playing critical roles in driving evolution, inducing variation, and regulating gene expression. In this review, we summarized the definition, arrangement, and structural characteristics of repeats. Besides, we introduced diverse biological functions of repeats and reviewed existing methods for automatic repeat detection, classification, and masking. Finally, we analyzed the type, structure, and regulation of repeats in the human genome and their role in the induction of complex diseases. We believe that this review will facilitate a comprehensive understanding of repeats and provide guidance for repeat annotation and in-depth exploration of its association with human diseases.

## Abstract (web summary)

This Review explores the characteristics of DNA repeats, their functional impacts, and available tools for their analysis.

## Introduction

Repetitive DNA sequences (repeats) are patterns of nucleic acids that occur in multiple copies throughout the genome^1^. Both eukaryotic and prokaryotic organisms contain a certain proportion of repeats in the genome^2–4^, particularly mammalians, in which repeats account for 25–50% of their entire genome (Supplementary Fig. S1). For instance, about 50% of the human genome consists of repeats^5^, while roughly 4% of human genes harbor transposable elements in their protein-coding regions^6^. Because many of these repeats (~89.5%) are located within introns, they have been erroneously assumed to be non-functional^7^. However, increasing research indicates the significant impacts that repeats in coding and noncoding regions can have on evolution, gene expression regulation, and variation induction^8–10^. For example, when repeats are present in the coding region they get translated canonically. Not only can non-coding repeats be translated by a non-canonical mechanism^11^, but even the telomeric repeat RNAs can get translated^12^. Moreover, recent studies have shown that such repeats are closely related to a variety of diseases, such as genetic disorders (e.g., Hemophilia), neurological diseases (e.g., poly-Q diseases), and cancers (e.g., endometrial, stomach and colorectal cancers)^13–15^. A glossary table (Supplementary Table S1) used to explain acronyms/terminologies in this study is shown in Supplementary Note 1.

**Fig. 1:**
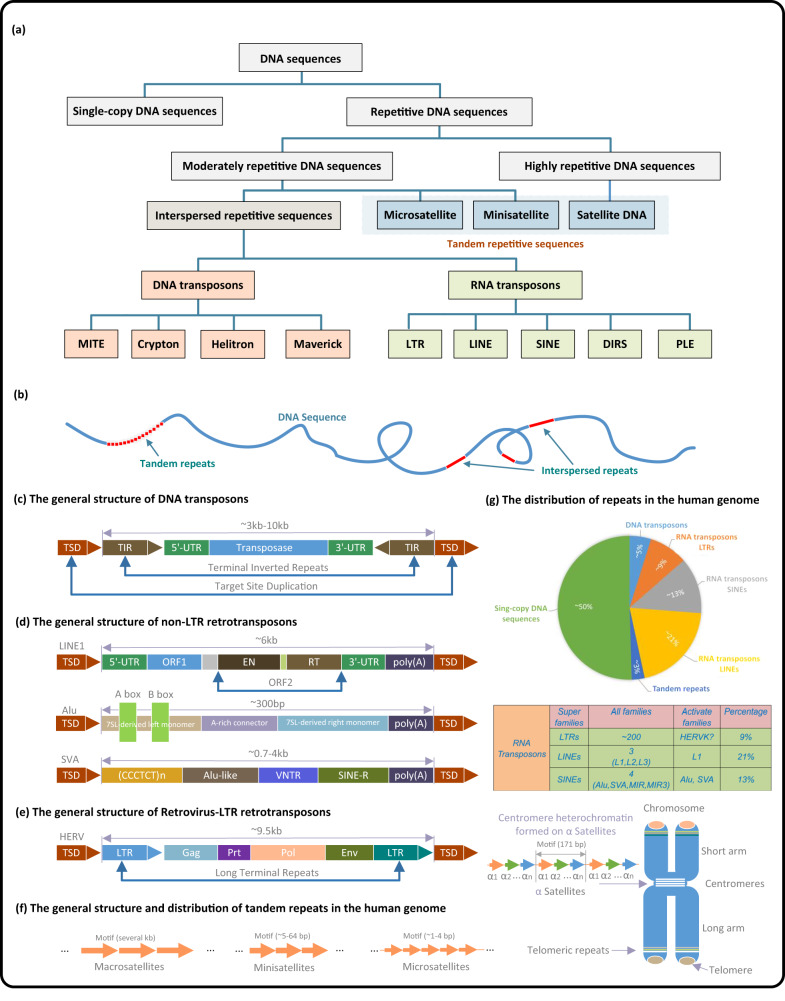
General classification of repeats, the typical structure of TEs and TRs, and the proportion of various types of repetitive elements in the human genome. Sub-graph (**a**): Classification of repeats in the human genome. Sub-graph (**b**): Arrangement and characterization of repeats in the human genome. Sub-graph (**c**): Typical structure of DNA transposons, in which TIR and TSD respectively represent the terminal inverted repeat and target site duplication. Sub-graph (**d**): Typical structure of *non-LTR* retransposons, in which the color blocks represent the protein domains contained in each family, and the gray block represents the non-coding regions. Sub-graph (**e**): Typical structure of retrovirus-like *LTR* retrotransposons, in which *LTR* represents the long terminal repeat. Sub-graph (**f**): Typical structure and distribution of TRs in the human genome. Sub-graph (**g**): Proportion of TRs and active TEs in the human genome. Specifically, *LINE-1* and *LINE-2* retransposons are represented by *L1* and *L2* respectively, while *SINE-VNTR-Alu* retrotransposon and Mammalian-wide interspersed repeats are represented by *SVA* and MIR. The color arrows represent the repetitive unit (or motif) of each kind of TR, and the light black structure represents the chromosome.

DNA sequences can be categorized into three groups according to their recurrence frequency^16^, as shown in Fig. 1(a). The first group is composed of high-frequency repeats, also known as satellite DNA sequences (**satDNAs**), which are found in various regions of the chromosomes, including pericentromeric, subtelomeric, and interstitial regions. These sequences typically form constitutive blocks of heterochromatin that are essential components of structures such as centromeres and telomeres^17^. The length of satDNA repeating units can vary from a few base pairs to over 1 kilobase pairs, forming arrays that can span up to 100 megabases and be repeated over 10^6^ times, making up ~8–10% of the human genome^18^.

The second group comprises moderate-frequency repeats that are typically 500–300,000 base pairs in length and repeated between 10 and 10^5^ times, accounting for ~30% of all repeats^19^. These repeats are further classified into two subcategories: (A) microsatellites and minisatellites (VNTR), and (B) dispersed repeats, which are primarily made up of transposable elements (**TEs**)^20^. It is worth noting that many moderate-frequency repeats have been implicated in gene expression regulation^21^.

The third group comprises unique, single-copy DNA sequences, which do not share homology with any other sequences in the genome. Examples of such sequences in the human genome include protein-coding genes (e.g., the *globin*, *ovalbumin*, and *silk fibroin* genes), non-coding RNAs, and regulatory elements that control gene expression^22,23^. Approximately 40–50% of the total human DNA sequences are single-copy DNA sequences, meaning that about half of the human genome is composed of unique and non-repetitive sequences.

According to the arrangement of repeating units, repeats can be classified into two types: tandem repeats (TRs) and interspersed repeats^24^, as depicted in Fig. 1(b). Interspersed repeats, also known as **transposons** or **TEs**, consist of DNA and RNA transposons^25^. Generally, TRs refer to a sequence array formed by the repeated occurrence of basic repeating units connected head-to-tail^26^ (Supplementary Note 2). TRs, especially satellite DNA, are clustered in specific chromosomal regions such as centromeres, tetramers, and telomeres, which play an essential role in cellular processes, including chromosome segregation, genome organization, and chromosome end protection^27^. For example, centromeres contain long tandem arrays of *alpha*-satellite repeats that extend over millions of base pairs and are organized in a hierarchical manner. The tandem arrays span between 100 and 5000 bp on different chromosomes, ranging from 0.2 to 10 Mb. Some of these arrays include 17 bp binding motifs for the centromere-specific DNA binding protein, which have been used to create synthetic human chromosomes^28^.

### Tandem repeats

Tandem Repeats in the human genome can be divided into the following subcategories: microsatellites, minisatellites, centromeric satellites, and telomeric and subtelomeric repeats (Fig. 1(f) and Table 1). The difference between microsatellites and minisatellites is represented in their length and frequency of occurrence. Microsatellites are DNA sequences of <5 bp units repeated in tandem and are most frequent in the human genome^29^. Minisatellites are tandem repetitions of more than 5 bp units, and their frequency in the human genome is relatively rarer than that of the former^30^. In the human genome, centromeric satellites can be classified into the *alpha*-satellite and Satellite II/III. Among them, Satellite II/III comprises of various variations on the *ATTCC* motif^31^. Telomeric repeats (satellites) are located at the telomeres, consisting of 300–8000 precise *CCCTAA/TTAGGG* motifs and covering a range of 2–50 kb on the end of the chromosomes^32^. Subtelomeric repeats are located in the boundary of 100–300 kb between the telomere and the remaining part of the chromosome, consisting of satellite-like sequences^33^. Type, length, frequency, and distribution of TRs in the human genome are summarized in Table 1 and Supplementary Table S2.

**Table 1:** Classes and length distribution of tandem repeats in the human genome.

Class of TRs	Length of TR unit	Length of TR array
Telomeres	~6 bp	~10–15 kb
Tandem paralogous		
rDNA	~43 kb	~3–6 Mb
Segmental duplications	~1–400 kb	~1kb–5Mb
Microsatellites	~2–6 bp	~10–100bp
Minisatellites	~10–100bp	~100bp–20kb
Satellites		
Alpha satellite	~171bp	~0.2–8Mb
Beta satellite	~68 bp	~60–80kb
Gamma satellite	~48–220bp	~11–121kb
Satellite I	~17–25bp	~2.5kb
Satellite II	~23–200bp	~11–70kb
Satellite III	~5bp	~3.6kb
Satellite IV	~35bp	~25–530kb
Macrosatellites	~100bp–5kb	~300kb
Megasatellites	~1–5kb	~400kb

A glossary table (Supplementary Table S1) included in supplementary, presenting detailed explanations for all acronyms and terminologies utilized in the manuscript.

### Transposons

Transposons are classified into RNA and DNA transposons, depending on their mode of transposition. RNA transposons use a cut-and-paste mechanism, where the transposase enzyme excises the transposon from its original location and inserts it elsewhere in the genome via an RNA intermediate. DNA transposons also use a cut-and-paste mechanism, but they move directly as DNA and are excised from their donor locus and reinserted elsewhere in a conservative mechanism. This divergence results in various dissimilarities in their transposition mechanisms and evolutionary trajectories. Typical structures of retrotransposons, transposons, and tandem repeats are illustrated in Supplementary Fig. S2(a),(b) and (c), respectively.

DNA transposons, also known as Class II transposons, can be classified into four super families based on their constituent structures and transposition patterns: miniature inverted-repeat TEs (*MITEs*), *Cryptons*, *Mavericks* (or *Polintons*), and *Helitrons*. *MITEs* are non-autonomous transposons primarily found in the non-coding regions of plant and animal genomes^34^, with the ability to alter gene structures and functions. *Cryptons* are a unique class of DNA transposons that use *Tyrosine Recombinase* (YR) to cut and reattach recombining DNA molecules^35^, allowing them to incorporate YR sequences and drive animal evolution. *Mavericks* are large DNA transposons commonly found in eukaryotic genomes, with 6 bp target site duplication (TSD) sequences and genes homologous to viral proteins^36^. *Helitrons* are recently discovered eukaryotic transposons present in many plant and animal species^37^, which propagate through a rolling circle mechanism but don’t generate terminal repeats or TSDs. DNA transposons are characterized by terminal inverted repeat sequences (TIRs), which are complementary to each other at the left and right ends of the transposon. These transposons, also known as jumping genes, can move and integrate into diverse genomic regions. Figure 1 (c) illustrates the general structure of DNA transposons in genomes. DNA transposons, which make up about 5% of the human genome^38^, are considered DNA fossils because no family of them currently remains active in most mammals, including humans^39,40^.

RNA transposons, also known as retrotransposons or Class I transposons, can be classified into five super families based on their structures and transposition patterns: Long terminal repeats (*LTRs*), Long interspersed nuclear elements (*LINEs*), Short interspersed nuclear elements (*SINEs*), Dictyostelium intermediate repeat sequence (*DIRS*), and Penelope-like elements (*PLEs*)^41,42^. *LTR* retrotransposons are related to retroviruses and have *LTRs* at their 5′ and 3′ ends, which likely originated from ancient retroviral infections^43^. *LINEs* contain an internal promoter that drives the expression of transposition machinery, including reverse transcriptase and an endonuclease^44^. *SINEs* depend on *LINEs* for their transposition, with specificity determined by their 5′ tails. Most *SINEs* are derived from tRNA, 7SL RNA, or 5s RNA and have an RNA-Pol III promoter^45,46^. *DIRS* retrotransposons, which have tyrosine recombinase, differ from integrases or endonucleases commonly used by retrotransposons for site-specific genomic integration^47,48^. *PLEs* share an ancestor with telomerase reverse transcriptases (TERTs) and have unique features in retroelement phylogeny^49^. In the phylogeny of reverse transcriptases (RTs), *PLEs* do not belong to the *LTR* or *non-LTR* retrotransposon groups but form a sister clade with TERTs. TERTs are major components of the telomerase complex that maintain the linear chromosome ends in most eukaryotes^50,51^.

The RNA transposons in the human genome can be classified into *LTR* and *Non-LTR* retrotransposons. *Non-LTR* retrotransposons lack *LTRs*, but contain genes for reverse transcriptases, RNA-binding proteins, nucleases, and sometimes the Ribonuclease H domain^52^. *LINE* and *SINE* are two remaining active super families contained in *non-LTR* retrotransposons of the human genome, consisting of *LINE1* (*L1*), *Alu*, and *SINE-VNTR-Alu* (*SVA*), three active families (Table 2). Many studies have suggested that *L1* may contribute to human cancers by mutating specific oncogenes or tumor suppressor genes in somatic cells^53^. For example, there is evidence that *APC tumor suppressor* gene failure is caused by the *L1* insertions, which may be an important factor in the development of colorectal cancer^54^. In addition, *Alu* elements are retrotransposons specifically present in primate genomes that can regulate gene function by providing canonical polyadenylation signals and play a critical role in the primate genomic diversity, causing complex diseases^55^. For instance, many complex human diseases, such as meningococcal disease, venous thromboembolism, obesity, and breast cancer, are related to the structural variants caused by *Alu* insertions^56^. Currently, *SVA* is more active than high-copy pseudogenes (e.g., processed ribosomal pseudogenes), and *SVA* insertions may alter gene expression and cause several human diseases^57^. For example, *SVA* regulates the expression of related genes whose insertions have been identified as a significant contributor to diseases such as *X*-linked dystonia-parkinsonism, Neurofibromatosis type 1, and hemophilia B^58^, through mechanisms, such as loss of function mutation, modulation of splicing, and deletions at the site of insertion. The general structures of *non-LTR* retrotransposons are presented in Fig. 1(d). The type, family, and length distribution of repeats, as well as a brief introduction to their biological functions, are shown in Supplementary Table S3.

**Table 2:** Active transposable elements (TEs) in the human genome.

TE	Super family	Family	Introduction
*Non-LTR*	*SINE*	*Alu*/*SVA*	The *Alu*, *SVA*, *MIR*, and *MIR3* are four *SINE* families found in the human genome^45^. The *Alu* and *SVA* families are the two active members of the *SINE* family. More than one million *Alu* elements are scattered throughout the human genome, with an average length of about 300 bp, cumulatively accounting for about 10.7% of the genome^214,215^. The *SVAs* are evolutionarily young and presumably mobilized by the *LINE-1* reverse transcriptase in trans^216^. Transposition of the *SVA* element requires the transposase encoded by the *LINE-1* element. An *SVA* element comprises the following five parts: a hexameric repeat, an *Alu*-like sequence, a *GC*-rich *VNTR*, *SINE*, and a *poly-A* tail (Fig. 1(D)). The *SVAs* are shorter than *LINEs* but longer than *SINEs*, and a canonical *SVA* is an average of 2 kb but *SVA* insertions may range in size from 700 to 4000 bp^217^. In the human genome, *SVAs* are present in about 2700 copies.
	*LINE*	*L1*	There are three *LINE* families in the human genome: *L1* (*LINE1*), *L2* (*LINE2*), and *L3* (*LINE3*)^44^. Comprising roughly 17% of the human genome, *L1* is the only member of the *LINE* family that is still functioning and contains over 500,000 copies. Older lineages (*L2* and *L3*) account for <4% of the human genome^218^.
*LTR*	*HERV*	*HERV-K*	Some features of exogenous retroviruses (e.g., human immunodeficiency virus (*HIV*), human T-cell lymphotropic virus (*HTLV*), etc.) are retained in human endogenous retroviruses (*HERVs*). The typical genetic structure of the *HERVs* consists of group-associated antigen (*gag*), polymerase (*pol*), and envelope (*env*) genes sandwiched between a pair of *LTR* regions^219^. According to several studies, one member of the *HERV-K*(*HML-2*) family continued to be active during the evolution of the human lineage, eventually generating a number of human-specific *HERV-K*(*HML-2*) loci^220^.

One type of repetitive element that is unique to the human genome is known as the Human Endogenous Retrovirus (HERV). HERVs are remnants of ancient retroviral infections that occurred millions of years ago and became integrated into the human genome. They comprise ~9% of the human genome and are considered to be a type of transposable element.

The general structure of retroviruses and *LTR* retrotransposons are similar^59^. Several *LTR* retrotransposons have similar open reading frames (*ORFs*) to those of retroviruses, consisting of the *gag* and *pol* (*pro*) genes and, in some cases, *env* and other accessory genes. The main difference between retroviruses and *LTR* is the presence of a functional envelope (*env*) gene in retroviruses, which is absent or nonfunctional in *LTR*retrotransposons^60^. The general structure of the *retrovirus-LTR* is illustrated in Fig. 1 (e). No retrotransposable *LTR* retrotransposons have been identified in the human genome, and no *LTR* retrotransposon insertions have been collected in the database of human mutations. However, many elements belonging to the young human endogenous retroviruses (*HERV*) family, such as *HERV-K* (*K* denotes a lysine-tRNA-specific primer binding site to initiate reverse transcription), have an individual *ORF* domain in their structure capable of translation and production of functional proteins^61^. Furthermore, *HERVs* and mammalian apparent *LTR* retrotransposons (MaLRs) are remnants of ancient retroviral infections found within the human genome. These genetic components are notable for their up-regulation after innate immune activation and are primarily regulated in the context of immunity (Table 2). Retroelements and isolated *LTRs*, as part of molecular evolution, may benefit the host by promoting plasticity and gene expression regulation (i.e., via promoters and *cis*-regulatory sequences)^62^. The expression of *HERV-K* envelope transcripts is typically undetectable in normal human breast tissues but is detectable in most breast cancer tissues^63^. Therefore, this expression pattern can be used as a new disease biomarker in clinical diagnosis. The general structure and distribution of tandem repeats, and the percentage of TE families in the human genome are illustrated in Fig. 1(f) and (g), respectively. The proportion of the most abundant repeats in the genomes of *Humans*, *Rice* and *Drosophila* is presented in Supplementary Fig. S1.

Sequence analysis techniques such as de novo assembly, multiple sequence alignment (MSA), sequencing error correction, SNP and variation detection are often impacted by repeats^64,65^. For example, they are a primary cause of assembly errors in contigs generated by de novo assembly^66^. Repeats also introduce ambiguity in MSA of sequencing reads, which can interfere with downstream sequencing error correction, SNP identification, variant detection, and gene expression abundance analysis^67,68^.

Ambiguous paths in assembly graphs such as *de Bruijn*, string, and overlap graphs are often caused by repeats. Repeats eventually form misassemblies and gaps in contigs, affecting the accuracy and completeness of assemblies and limiting downstream applications (Supplementary Fig. S3(a) and (b))^69^. Obtaining accurate sequence composition of highly complex short TRs (STRs) in regions such as telomeres, subtelomeres, and centrioles through de novo assembly is challenging^70^. This limitation severely restricts the study of these regions. Repeats also pose a significant challenge to multiple sequence alignment (MSA), complicating alignment position determination and reducing the performance of sequencing error correction and the sensitivity of detecting SNPs, indels, and other mutations (Supplementary Fig. S3(c))^71^. A summary of the challenges posed by repeats for sequence analysis is provided in Supplementary Note 3.

## Biological functions of repeats and their roles in the human genome

Repeats play crucial roles in biological processes with both functional and non-functional implications. Certain repeats, like promoter and enhancer repeats, regulate gene expression by acting as binding sites for regulatory proteins. They also serve as structural elements, such as centromeres and telomeres, which are vital for genome stability and cell division. Moreover, repeats drive genome evolution through duplication, recombination, and transposition processes. Most repeats in the human genome are derived from TEs, which can move within the genome and act as regulatory elements controlling gene transcription, splicing, and genome architecture, potentially causing mutations or altering genome size and structure^72^ (Supplementary Fig. S4). In addition, TRs can alter the chromatin structure and affect transcription, leading to gene expression and protein abundance changes, although they represent only a tiny fraction (e.g., TRs accounted for only ~ 3%, as shown in Fig. 1(g)) of the human genome (Supplementary Fig. S5). The biological functions of repeats and their roles in the human genome are discussed in the following sections, and several typical examples of their influence are summarized in Supplementary Note 4.

### Biological functions of transposable elements

**Fig. 2:**
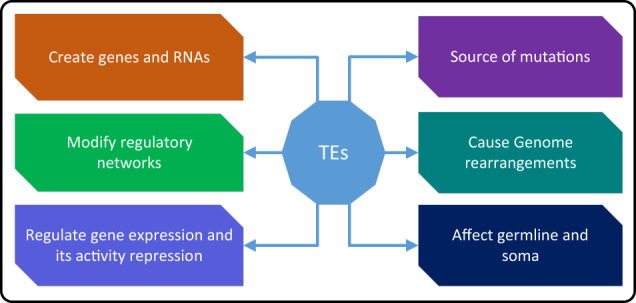
How TEs affect the genome. TEs can directly or indirectly affect the genome through some specific mechanisms.

The movement of TEs may result in mutations, alter gene expression, induce chromosome rearrangements, and enlarge genome sizes due to increased copy numbers^73^. Thus, they are considered an essential contributor to gene and genome evolution^74^. In addition, TEs have also been recognized as promising candidates for stimulating gene adaptation through their ability to regulate the expression levels of nearby genes^75^. Furthermore, combined with their mobility, TEs can relocate adjacent to their targeted genes and control the expression levels of those genes, depending on the circumstances^76^. The illustrations in Fig. 2 and Supplementary Fig. S4 show how the genome can be affected by TEs in direct or indirect ways.

#### Transposable elements can cause mutations and genetic polymorphisms

Many TE families are still active and undergoing constant transposition. Variations are induced when TEs transpose nearby genes and regulatory regions, and these are often rare mutations under purifying selection. For example, an experimental study revealed that the spontaneous insertion of multiple TEs causes more than 50% of all known phenotypic mutants in *D. melanogaster*^77^. Another experimental study found that ~10–15% of inherited mutant phenotypes in the mouse genome are caused by the autonomous activity of a family of persistently active *LTR* retransposons^8^. Furthermore, in another study^78^, the researchers found that the average difference between any two human haploid genomes is caused by ~1000 TE-dominated insertions, primarily from the *L1* or *Alu* families. The primary mechanisms by which TEs cause mutations and genetic polymorphisms are described subsequently:

##### Insertion

TEs can insert themselves into new genomic locations, which can result in various types of mutations^79^. When TEs insert into protein-coding regions, they can disrupt the reading frame, introduce premature stop codons, or alter splicing patterns, leading to loss-of-function mutations. Insertion into regulatory regions can disrupt the binding sites of transcription factors or other regulatory elements, affecting gene expression levels or patterns. These insertional mutations can result in genetic variations and contribute to phenotypic diversity.

##### Retrotransposition

Retrotransposons, a type of TE, can undergo retrotransposition, where they are transcribed into RNA and then reverse transcribed back into DNA, leading to reintegration at a new genomic location. This process can result in the duplication of TEs and adjacent genomic sequences, creating copy number variations^80^. Retrotransposition can also lead to the formation of processed pseudogenes, which are nonfunctional copies of genes^81^. The repeated retrotransposition events of TEs can generate genetic polymorphisms and contribute to the evolution of genomes.

**Fig. 3:**
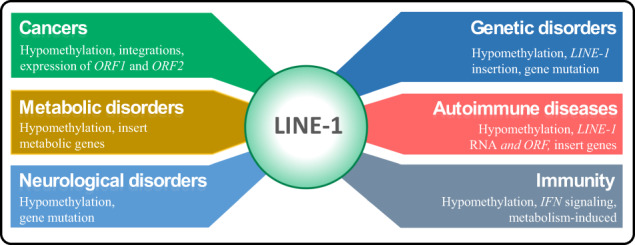
The association between the *L1* transposon and some complex diseases and its regulatory mechanism^233^. For example, hypomethylation, aberrant integration, and highly expressed *ORF1* and *ORF2* domains of *L1* are related to cancers and thus serve as markers for cancer diagnosis.

In the human genome, gene mutations and the formation of malignant tumors may be caused by active TEs transposition (Supplementary Note 4). For example, *LINEs* are a group of *non-LTR* retrotransposons and are widespread in the genome of many eukaryotes. *L1* is the only abundant and active *LINE* in the human genome, and the human genome contains an estimated 100,000 truncated and 4000 full-length *L1* elements accounting for about 17% of the entire genome^82^. Since *L1* correlations with disease and immunity by producing gene mutations, it has become a significant hallmark of several cancers (e.g., ovarian, endometrial, breast, colon, kidney, etc.) and other disorders (Supplementary Table S4). The associations between *L1* and some complex diseases and its regulatory mechanism are presented in Fig. 3. In addition, *L1* promotes the occurrence of malignant tumors through three main mechanisms: hypomethylation, aberrant integrations, and high expression of its internal *ORF1* and *ORF2* domains^83,84^. The relationship between *L1* and gene mutations producing malignant tumors is introduced in Supplementary Note 4. Another well-known example is the *Alu* element, a type of *SINE*, which can disrupt gene regulation and contribute to genomic diversity and disease susceptibility^85^. Furthermore, one study reported an association between *SVA* insertions and neurological diseases such as Parkinson’s disease and amyotrophic lateral sclerosis^86^. In addition, a recent research has indicated that *HERV-KHML-2* insertions can contribute to somatic mosaicism and influence gene expression in certain tissues, potentially impacting disease development^87^.

#### Transposable elements can regulate gene expression and activity repression

The TE transposition is an essential factor in gene expression variation, often resulting in extreme gene expression changes much more significantly than those produced by rare SNPs^88^. Involvement in gene expression regulation is another crucial function of TEs in the human genome. There are two primary mechanisms by which TEs regulate gene expression. First, they provide *cis*-regulatory sequences in the genome with intrinsic regulatory properties for their expression, making them potential regulators of host gene expression. Second, TEs can encode regulatory RNAs. A growing number of studies have demonstrated that their sequences are found in most miRNAs and long noncoding RNAs (lncRNAs), implying that these RNAs are derived from TEs^89^. Moreover, TEs can be activated or repressed under stress conditions. In some cases, the repression of TEs occurs after the initial activation^90^. For instance, to suppress TEs activity, host cells have developed a variety of mechanisms, including epigenetic pathways, such as DNA methylation and histone modifications. The primary mechanisms by which TEs regulate gene expression and activity repression are described subsequently:

##### Epigenetic modification

TEs can influence gene expression by modifying the epigenetic landscape of the genome. TEs often contain regulatory sequences, such as promoters and enhancers, that can interact with nearby genes. The presence of TEs can attract epigenetic modifiers, resulting in the deposition of repressive chromatin marks, such as DNA methylation and histone modifications. These epigenetic modifications can lead to gene repression or silencing by preventing the binding of transcription factors and the access of transcriptional machinery to gene regulatory regions. Conversely, some TEs may also act as regulatory elements, promoting gene activation when demethylated or associated with activating chromatin marks.

##### Production of non-coding RNAs

TEs can generate non-coding RNAs, such as long non-coding RNAs (lncRNAs) and small interfering RNAs (siRNAs), that play a role in gene regulation. TEs can serve as transcriptional starting sites for the production of lncRNAs, which can interact with chromatin and modulate gene expression. In addition, TEs can be transcribed into siRNAs, which can then guide RNA-induced gene silencing complexes to complementary mRNA sequences, leading to the degradation or repression of target transcripts.

In the human genome, more than 60% of *SVAs* are within genes or located in their 10 kb flanking regions^57^. Moreover, *SVAs* could recruit transcription factors and influence the local chromatin structure, regulating the transcription and expression of nearby genes, as has been demonstrated for human endogenous retroviruses, causing a region to become either accessible or inaccessible to transcriptional machinery. Specifically, how it is regulated depends on the epigenetic marks spread throughout the element^91^. As described in the previous chapters, the hypomethylation of retrotransposable elements has become an epigenetic mark of several diseases (Supplementary Note 5), such as cancers (Supplementary Fig. S6(a),(b) and (c)). As demonstrated by the regulatory role of *L1s* in cancer, and changes in epigenetic marks of *SVAs*, such elements are inappropriately reactivated, possibly leading to the dysregulation of neighboring genes and their associated pathways (Supplementary Fig. S7(a)). For example, a recent study highlighted that certain *SVA* insertions can act as enhancers and influence the expression of nearby genes in a tissue-specific manner^92^. Another recent study have shown that *Alu* elements can act as enhancers or repressors and contribute to tissue-specific gene regulation^93^. The relationship between *SVAs* and gene expression regulation is presented in Supplementary Note 5.

#### Transposable elements can associate with genome rearrangement

In reality, TEs can be associated with genome rearrangement through various mechanisms, such as de novo TE insertion, TE insertion-mediated deletion, and homologous recombination between them. These rearrangements increase the genomic difference between genomes, and some specific rearrangements may lead to complex diseases^94^. As an illustration, the expression of retrotransposition-competent TEs may result in additional insertions, which may affect the expression or function of genes^95^ and trigger chromosome rearrangements through an ectopic recombination between repeated copies of a TE, causing mutations^96^, resulting in several complex diseases, such as cancers^97^, Alzheimer’s disease^98^, and autoimmune and neurological disorders^99^. The primary mechanisms by which TEs associate with genome rearrangement are described subsequently:

##### Transposition

TEs are mobile genetic elements that can undergo transposition, a process in which they move from one genomic location to another. During transposition, TEs can insert themselves into new sites within the genome, leading to rearrangements. For example, when TEs transpose and insert themselves between genes, they can disrupt gene order, create gene duplications, or cause gene deletions. These structural changes can have significant effects on the organization and function of the genome.

##### Recombination

TEs can serve as recombination sites in the genome, promoting genomic rearrangements. In some cases, recombination events between different TEs or between TEs and their target sequences can result in large-scale genomic rearrangements. This includes chromosomal inversions, translocations, and deletions, which can alter gene order, disrupt regulatory elements, and impact the overall genomic architecture.

Compared to other TEs, *Alu* and *L1* elements in the human genome are more likely to cause genomic rearrangements due to their widespread presence. Specifically, 492 *Alu* recombination-mediated deletions (ARMDs) have been identified in the human genome, deleting ~400 kb of human genomic sequences, including exons of known or predicted genes^100^. The ARMD process has significantly contributed to genomic and phenotypic variations between humans and chimpanzees since their evolutionary divergence. For another example, a recent research suggests that *L1* insertions can cause genomic rearrangements, including deletions, inversions, and duplications, leading to structural variations in the human genome^101^. The specific relationship between genome rearrangements caused by TEs and complex diseases is discussed in Supplementary Note 6.

#### Transposable elements can act as insertional mutagens in germline and somatic cells

Mobile elements, such as *L1*, *Alu*, *SVA* and *HERV-K*, are in charge of novel germline insertions, which may lead to genetic illness (Table 3) (Supplementary Note 6.1 to Note 6.7). The primary mechanisms by which TEs act as insertional mutagens in germline and somatic cells are described subsequently:

**Table 3:** The association between repeats and human diseases.

Repeat	Family/Motif	Gene/Loci	Disease/genetic disorders
	*Alu*	*APC*	Colon cancer
	*Alu*	*BRCA1*	Breast cancer/ovarian cancer
	*Alu*	*BRCA2*	Breast cancer/ovarian cancer
	*Alu*	*MLVI2*	Leukemia
	*Alu*	*NF1*	Neurofibromatosis type I
	*Alu*	*F8*	Hemophilia A
	*Alu*	*U2AF65*	Loss of hnRNP C binding, leading to aberrant exonization
	*Alu*	*OAT*	OAT deficiency
	*Alu*	*COL4A3*	Alport syndrome
	*Alu*	*GUSB*	Sly syndrome
	*LTR*	*BAAT*	Breast cancer/ovarian cancer
*TEs*	*LTR*	*MSLN*	Cancer
	*LTR*	*ADH1C*	Role in alcoholism
	*LTR*	*HSD17B1*	Breast cancer
	*L1*	*FKTN*	Fukuyama-type congenital muscular dystrophy
	*L1*	*DMD*	Duchenne muscular dystrophy
	*L1*	*CYBB*	Chronic granulomatous disease
	*L1*	*RP2*	X-linked retinitis pigmentosa
	*L1*	*CYBB*	Chronic granulomatous disease
	*L1*	*PDHX*	Pyruvate dehydrogenase complex deficiency
	*L1*	*RPS6KA3*	Coffin-Lowry syndrome
	*(CAG)n*	*Androgen Receptor (AR) gene*	Prostate cancer
	*(AT)n*	*Adenomatous Polyposis Coli (APC) gene*	Sporadic colorectal cancers
	*(ATTCT)n*	*the intron 4 of the gene SPATA31*	hepatocellular carcinoma (HCC)
	*(CGG)n*	*FMR1 gene*	Autism spectrum disorder (ASD)
	*(CAG)n*	*HTT exon*	Huntington disease
*TRs*	*(GCN)n*	*HOXD13 exon*	Synpolydactyly, type 1
	*(CTG)n*	*DMPK 3’UTR*	Myotonic dystrophy type 1 (DM1)
	*(CGG)n*	*FRAXA 5’UTR*	Fragile X syndrome
	*(GAA)n*	*FRDA exon*	Friedreich ataxia
	*(CCTG)n*	*ZNF9 intron*	Myotonic dystrophy (DM2)
	*(ATTCT)n*	*ATXN10 intron*	Spinocerebellar ataxia, type 10
	*(TGGAA)n*	*TK2/BEAN intron*	Spinocerebellar ataxia, type 31
	*(GGCCTG)n*	*NOP56 intron*	Spinocerebellar ataxia, type 36
	*(GGGGCC)n*	*C9orf72 intron*	Amyotrophic lateral sclerosis, frontotemporal dementia (FTD)

The relationships between TEs and diseases were summarized from refs. ^55,58,78,221^. Similarly, the associations between TRs and diseases were summarized from refs. ^222–224^.

##### Disruption of coding sequences

When a TE inserts within a coding region of a gene, it can disrupt the reading frame, introduce premature stop codons, or cause other structural changes. This disruption can lead to the loss of gene function or the production of truncated and non-functional proteins. In germline cells, such mutations can be inherited and contribute to genetic variation in subsequent generations.

##### Alteration of regulatory elements

TEs can insert near regulatory elements, such as promoters, enhancers, or insulators, and disrupt their function. This can result in the misregulation or aberrant expression of genes. Changes in the regulation of critical genes can have profound effects on cellular processes, development, and disease susceptibility.

For instance, a study has revealed that over 120 independent TE insertions are essential contributors to human diseases, including hemophilia, Dent disease, neurofibromatosis and cancers^102^. The germline transposition rate for the *Alu* element in humans is about 1 in 21 births^103^, while the corresponding value for the *L1* element is about 1 in 95 births^104^. Historically, TEs have generally been considered transcriptional silencing in somatic cells. However, evidence indicates that active TEs are also present in the somatic cells of various organisms. As an illustration, the expression and transposition of the *L1* element have been identified in several somatic contexts, such as early embryos and specific stem cells^105^. Furthermore, *HERV-K* elements have been implicated in insertional mutagenesis. Recent studies have identified *HERV-K* insertions with potential mutagenic effects on nearby genes, including cancer-related genes^106^ (Supplementary Fig. S7(b)). Human cancers have also exhibited somatic activity, with tumors able to pick up hundreds of additional *L1* insertions. For instance, recent research has highlighted the impact of *L1* insertions in diseases such as cancer, neurological disorders, and genetic syndromes^107^.

#### Transposable elements can drive key coding and non-coding RNAs

According to mounting evidence, TE insertions may serve as the building blocks for forming protein-coding genes and non-coding RNAs that can carry out the crucial physiological functions of cells^108^. For example, *Rag1* and *Rag2* are spectacular examples of deeply conserved TE-derived genes that activate V(D)J somatic recombination in the immune system of vertebrates^109^. As another example, based on a mixed lncRNA annotation from RNA sequencing and GENCODE (a scientific project in genome research and part of the ENCODE scale-up project), a study estimated that 41% of lncRNA nucleotides are derived from TEs, and the majority of lncRNAs (about 83%) contain at least one TE fragment^110^. The primary mechanisms by which TEs drive key coding and non-coding RNAs are described subsequently:

##### Retrotransposition

TEs, particularly retrotransposons, can undergo a process called retrotransposition where they are transcribed into RNA and then reverse transcribed back into DNA, leading to their insertion into new genomic locations. If these retrotransposed elements land within or near functional genes, they can act as alternative promoters, enhancers, or splice sites, giving rise to new coding and non-coding RNA transcripts. This process can generate novel RNA molecules with potentially functional roles in cellular processes.

##### Co-option of regulatory elements

TEs often contain regulatory sequences such as promoters, enhancers, and insulators. These sequences can be co-opted by the host genome to regulate the expression of nearby genes or to shape the expression patterns of non-coding RNAs. By providing alternative regulatory elements, TEs can impact gene expression networks and contribute to the production of key coding and non-coding RNAs.

The presence of TEs that drive key coding and noncoding RNAs in the human genome may be associated with certain diseases (Table 3). For instance, *HERVs* affect human health and cause disease by encoding proteins, acting as promoters/enhancers or lncRNAs, accounting for about 9% of the human genome^111^. *HERVs* can also have a direct effect via their proteins in the development of cancers. For example, by inducing cell-cell fusion or epithelial-to-mesenchymal transition, *HERV* envelope proteins play a critical role in tumorigenesis and development in melanoma, endometrial carcinoma, and breast cancer^112^. Furthermore, *HERVs* can generate lncRNAs that promote cancer proliferation, motility, and invasion. For example, in the study^113^, researchers have found that several *HERVs*-derived lncRNAs, such as *UCA1*, *SAMSON*, and *BANCR*, are involved in the processes of proliferation, motility, and invasion in bladder cancer and melanoma. The relationship between transcriptional activation of *HERV* retrotransposons and human cancer is summarized in Supplementary Note 6.7.

#### Transposable elements can alter transcriptional networks and conduce to *cis*-regulatory DNA elements

*Cis*-regulatory DNA elements (CREs) are regions of non-coding DNA that regulate the transcription of neighboring genes. In addition, CREs are vital components of genetic regulatory networks. Some TEs have evolved into CREs, whose function is to mimic host promoters, enabling them to recruit host-encoded factors driving their selfish transcription^114^. For instance, due to innate and adaptive immune responses, the immune system can protect organisms from pathogens and foreign substances. During evolution, some TE families, including many endogenous retroviruses (*ERVs*), have the capacity to influence and shape transcriptional networks. They can function as signaling molecules that regulate DNA elements and the immune system^115^. The primary mechanisms by which TEs alter transcriptional networks and conduce to *cis*-regulatory DNA elements are described subsequently:

##### Enhancer hijacking

TEs can integrate near enhancer regions, affecting the binding of transcription factors and changing the regulation of nearby genes.

##### Promoter modulation

TEs can also insert near gene promoters, influencing the recruitment of transcriptional machinery and impacting gene expression levels.

In the human genome, *L1* elements have the potential to influence transcriptional networks. Recent research has demonstrated that *L1* retrotransposition can introduce novel regulatory elements, alter gene expression patterns, and contribute to cellular diversity^116^. Furthermore, *Alu* elements can also impact transcriptional networks. Recent studies have highlighted their role in shaping tissue-specific gene expression, alternative splicing, and influencing the expression of neighboring genes through enhancer or promoter activities^117^. The diverse mechanisms through which TEs influence host gene-regulatory networks can be broadly categorized into five classes: (1) introduction of transcription factor binding sites, promoters, and enhancers, (2) modification of 3D chromatin architecture, (3) production of regulatory non-coding RNAs, (4) usage of TE-derived coding sequences as new transcriptional effector proteins, and (5) secondary effects of TE silencing mechanisms^118^.

### Biological functions of tandem repeats

**Fig. 4:**
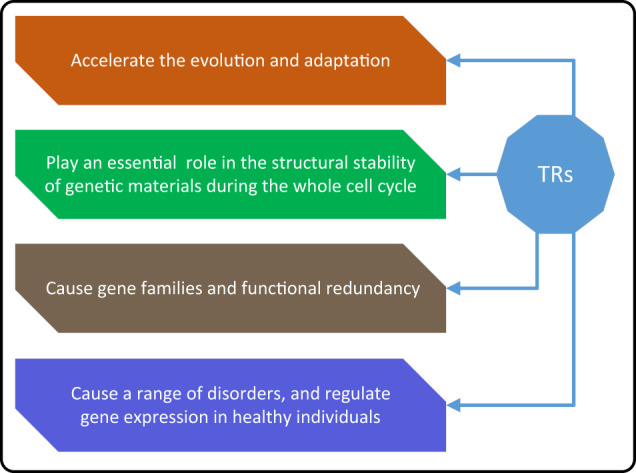
How TRs affect the genome. Similar to TEs, TRs can also affect the genome in specific ways.

TRs are common features of both prokaryote and eukaryote genomes. For example, more than one million distinct TRs are contained in the human genome, many of which are highly polymorphic in sequence composition and copy number. TRs can be found in intergenic regions and in both the non-coding and coding regions of a variety of genes^119–121^. Moreover, TRs occur near or between a series of genes and can affect the structure and function of DNA, RNA, and proteins through specific mechanisms and produce a series of molecular and cellular consequences^122^. As an illustration, many TRs are involved in biological functions in a copy number-dependent manner, and there is evidence that TRs may regulate the expression of nearby genes by altering their copy number^123^. In general, TRs are highly mutable and can be located in exons, introns, or intergenic regions, providing opportunities for the modulation of gene expression, as well as the structure and function of RNAs and proteins^124^. Expanded TRs usually cause various disorders, including autism spectrum disorder (ASD) and cancers (Table 3 and Supplementary Table S5). The illustrations in Fig. 4 and Supplementary Fig. S5 highlight how TR can directly or indirectly affect the genome.

#### Tandem repeats can accelerate evolution and adaptation

TRs are often referred to as satellite DNA, which can be further classified into microsatellites or short tandem repeats (STRs) (motif length: 1–4 bp), minisatellites (motif length: 5–64 bp), and macrosatellites (motif length: several kp), according to the size of the repeated motifs^125^. For example, slipped strand mispairing is a mutation process that occurs during DNA replication, which is one explanation for the origin and evolution of repetitive DNA sequences^126^. TRs, especially STRs, are extremely unstable in terms of length, sequence composition, and copy number, with mutation rates typically 10–100,000 times higher than in other parts of the genome^127^. These unstable repeats are found in up to 20% of eukaryotic genes and promoters, where they confer phenotypic or functional variability on the cell surface and extracellular proteins and have pathological consequences. The primary mechanisms by which TRs accelerate evolution and adaptation are described subsequently:

##### Rapid genetic variation

TRs undergo rapid changes in copy numbers and lengths, creating genetic diversity that can drive the emergence of new traits.

##### Gene regulation

TRs located in regulatory regions can influence gene expression, allowing for adaptive changes to occur in response to environmental pressures.

In the human genome, TRs are also frequently found in genes that control body morphology^128,129^. For example, compared with synteny blocks, evolutionary breakpoint regions in the human genome contain more base pairs associated with TRs, with AAAT being the most frequent motif^130^. These TRs within evolutionary breakpoint regions have the potential to facilitate and accelerate gene expression evolution and generate sufficient variability to drive the rapid evolution and adaptation of organisms^131^. Furthermore, recent studies have shown that STR variations in immune genes, such as HLA loci, can shape immune responses and contribute to adaptation to diverse environments^132^. In addition, TRs located in regulatory regions can facilitate evolutionary adaptations. Recent research has suggested that expansion or contraction of STRs within regulatory regions can modulate gene expression and contribute to phenotypic variation and adaptive responses^133^.

#### Tandem repeats can play a critical role in the structural stability of genetic materials during the cell cycle

Within or around certain specialized chromosomal regions (e.g., centromeres, telomeres, and subtelomeres), TRs may play crucial roles in the structural stability of genetic materials during the cell cycle^134^. The primary mechanisms by which TRs play a critical role in the structural stability of genetic materials during the cell cycle are described subsequently:

##### Replication fork stabilization

TRs, consisting of repeated DNA sequences adjacent to each other, can stabilize the replication forks during DNA replication. The repetitive nature of TRs provides a stable template for DNA polymerases to bind and initiate replication. This stability prevents replication forks from stalling or collapsing, ensuring accurate and complete DNA replication. TRs act as essential structural elements that contribute to the stability of genomic regions during the cell cycle.

##### Telomere maintenance

Telomeres, specialized TRs located at the ends of chromosomes, play a crucial role in maintaining genomic stability. Telomeres protect the ends of chromosomes from degradation, fusion, and recognition as DNA breaks. During each round of DNA replication, the conventional DNA replication machinery has difficulty fully replicating the ends of linear chromosomes. Telomeres, with their repeated sequences and associated proteins, form a protective cap that allows complete replication of chromosome ends and prevents the loss of genetic information. Telomeric TRs, in conjunction with telomerase enzyme activity, ensure the integrity and stability of the genome during successive cell divisions.

For instance, centromeres are chromosomal domains responsible for the faithful transmission of genetic material during cell division. They are characterized by highly repetitive DNA regions and bound kinetochore proteins, and they are required for the attachment of microtubules to the chromosomes during mitosis^135^. An array of tandem repeats known as *alpha*-satellites is one of the crucial components of centromeres, and it plays a vital role in maintaining the stability of human chromosomes. Variations in *alpha*-satellites can impact the function of the centromere^136^. In addition, telomeres consist of repeat sequences and are bound by multiple telomeric interacting proteins. In mammalian cells, telomere DNA is composed of double-stranded tandem repeats of *TTAGGG*, with terminal 3′ G-rich single-stranded overhangs. Telomeres are protected by protein complexes, such as shelterin, which includes *TRF1*, *TRF2*, *POT1*, and other proteins that interact with telomeres indirectly^137^. This protection distinguishes natural chromosome ends from accidental DNA breaks and prevents unwanted repair machinery activity on telomeres.

Furthermore, the 5′ and 3′ UTRs of genes are transcribed but usually not translated into proteins. However, they contain various regulatory elements involved in post-transcriptional gene regulation, such as mRNA stability, localization, and translation efficiency^138^. STRs within UTRs can contribute to gene regulation in the following ways: (1) Modulation of mRNA stability: STRs in the UTRs can impact the stability of mRNA molecules. Changes in STR length may affect the folding of UTRs, leading to altered interactions with RNA-binding proteins and subsequent degradation or stabilization of mRNA. (2) Regulation of translation efficiency: UTRs can also influence translation initiation and efficiency. STRs located in the 5′ UTRs can affect ribosome binding and start codon recognition, leading to changes in translation rates and protein production. STR variations in UTRs have been associated with complex traits and diseases. For instance, a recent study identified UTR STR expansions associated with the risk of neurodevelopmental disorders^139^.

In addition, TRs can be transcribed into RNA molecules through the process of transcription, which is carried out by RNA polymerases^140^. When these TRs are transcribed into RNA, the resulting RNA molecules can exhibit structural features and functional implications. The structure of TRs in terms of transcribed RNA are as follows: (1) Transcribed RNA molecules derived from TRs retain the repetitive nature of the underlying DNA sequence. (2) TR RNA can fold into various secondary structures due to intra-molecular base pairing within the repetitive sequence. (3) TR-derived RNA molecules can serve diverse non-coding RNA functions. For example, some TR RNAs act as scaffolds for the assembly of ribonucleoprotein complexes or regulate gene expression through interactions with RNA-binding proteins or microRNAs. (4) TR-derived RNA can engage in regulatory mechanisms such as RNA interference, where complementary TR RNA pairs with target mRNA to modulate its stability or translation. TR RNA molecules can also influence cellular processes by sequestering RNA-binding proteins or acting as decoys for regulatory factors. (5) Expansions or contractions of TRs in transcribed RNA have been linked to various genetic diseases. Abnormal TR RNA structures and interactions can result in functional consequences, including the sequestration of RNA-binding proteins, disruption of cellular processes, or induction of toxic effects. These factors contribute to the pathogenesis of diseases^141,142^.

#### Tandem repeats can result in redundancy of gene families and functions

A gene family is a collection of many related genes that typically perform comparable biological tasks. Individual members of clustered gene families are often responsible for achieving specific phenotypes or functions in the overall mission^143^. Tandem gene duplication is thought to have significantly contributed to the evolution of large gene families, genetic and morphological diversity, and speciation in eukaryotes^144,145^. The primary mechanisms by which TRs result in redundancy of gene families and functions are described subsequently:

##### Gene duplication

TRs can undergo replication slippage during DNA replication, leading to the expansion of the repeat region and subsequent gene duplication. This process can result in the creation of additional copies of genes within the same genomic region. The duplicated genes are often subject to variations, such as point mutations or insertions/deletions, that accumulate over time, leading to divergence in their sequences and functions. This duplication and subsequent diversification of gene copies can result in redundancy within gene families, where multiple genes have similar or overlapping functions.

##### Divergent evolution

Over time, duplicated genes arising from TRs can undergo divergent evolution. Mutations and genetic changes accumulate in each gene copy, resulting in alterations to their coding sequences and regulatory elements. These changes can lead to functional divergence, where duplicated genes acquire different functions or have differential expression patterns. As a result, redundant gene copies can contribute to the expansion and diversity of gene families, providing evolutionary opportunities for gene innovation and adaptation to new environmental or physiological contexts.

For example, the genes responsible for coding ribosomal RNA (rRNA) are present in the human genome as numerous tandemly arrayed copies. These ribosomal DNA (rDNA) repeats facilitate the production of abundant amounts of rRNA to satisfy the cell’s constant requirement for ribosome production^146^. In mammals, rDNA repeats are present in two types of tandem arrays, termed the 5S and 47S (or 45S) arrays. The 5S rDNA repeats are located in one large tandem repeat array on chromosome 1 in humans. The 47S arrays are located on the short arms of five acrocentric chromosomes in humans (chr. 13, 14, 15, 21, 22)^147^. Research conducted by the Chinese Academy of Sciences investigated the impact of TR-mediated expansions and variations within the mucin gene family. These TR expansions and variations contribute to the redundancy and functional diversification of mucins, which play important roles in various cellular processes^148^.

#### Tandem repeats can regulate gene expression, and their expansion can cause a range of disorders

TR instabilities, especially microsatellite instability, contribute significantly to causing gene expression variation in humans^149^, and numerous disorders such as cancer, ASD, Huntington’s disease, various ataxias, motor neuron disease, frontotemporal dementia, and fragile X syndrome, are associated with the expansion of TRs, particularly STRs^150–154^ (Table 3). The primary mechanisms by which TRs regulate gene expression, and their expansion can cause a range of disorders are described subsequently:

##### Transcriptional modulation

TRs located within gene regulatory regions, such as promoters and enhancers, can influence gene expression by affecting the binding of transcription factors. The presence of TRs can alter the three-dimensional chromatin structure, leading to changes in the accessibility of regulatory elements and the recruitment of transcriptional machinery. The variability in TR length and sequence can impact the affinity of transcription factors for binding sites, resulting in differential gene expression levels.

##### Epigenetic regulation

TRs can act as susceptible targets for epigenetic modifications, such as DNA methylation and histone modifications. The length and sequence composition of TRs can influence the degree of epigenetic regulation. Methylation of TRs, for example, can lead to the formation of repressive chromatin and transcriptional silencing. These epigenetic modifications can have a profound impact on gene expression patterns and contribute to the regulation of various cellular processes.

##### Alternative splicing

TRs within exons or introns can affect alternative splicing, a process that generates multiple mRNA isoforms from a single gene. Variation in TR length can influence the splicing process by altering the stability of RNA secondary structures or serving as binding sites for splicing factors. This can result in the inclusion or exclusion of specific exons, leading to the production of different protein isoforms with distinct functions or regulatory properties.

##### Expansion

The expansion of TRs can also cause a range of disorders, known as trinucleotide repeat expansion disorders. When the size of certain TRs exceeds a threshold, it can lead to genomic instability and pathological consequences. The expanded TRs can exhibit a tendency for further expansion and accumulation in subsequent generations, resulting in a dynamic and progressive increase in repeat length. The expanded TRs can interfere with gene function, leading to impaired protein production, altered protein structure, or disrupted cellular processes. Trinucleotide repeat expansion disorders include conditions like Huntington’s disease, Fragile X syndrome, and several forms of spinocerebellar ataxia, among others. These disorders often display a correlation between the size of the repetitive expansion and the severity of the disease phenotype.

For example, Lynch syndrome is an autosomal dominant disorder that increases the risk of developing colorectal cancer, endometrial adenocarcinoma, and tumors of the small intestine, stomach, ureter, renal pelvis, ovary, brain, and prostate. Research in study^155^ has demonstrated that most (90%) colorectal cancer due to Lynch syndrome have microsatellite instability. In addition, researchers in study^156^ have revealed that one neurodegenerative disease in which microsatellite instability contributes to a substantial number of cases is amyotrophic lateral sclerosis (ALS), a rapidly progressive and uniformly fatal motor neuron disease. Recent research indicates that TR polymorphisms can also regulate gene expression in healthy individuals^133^. Furthermore, TR instability can lead to reduced gene expression, increased disease incidence, and enhanced tumor aggression (Supplementary Fig. S7(c) and (d)). The association between tandem repeat instabilities and cancer, autism, as well as neurological disorders, is discussed in Supplementary Note 6.8 and Note 6.9.

## Repeat detection

Numerous computational methods have been proposed for identifying repeats in genomes, which can be divided into homology-based, structure-based, de novo methods, and hybrid frameworks, as shown in Table 4 and Supplementary Fig. S8.

**Table 4:** Introduction of typical repeats detection methods.

Method type	Method name	Description/Characteristic	Advantages/Disadvantages	References
	Censor^a^	Censor consists of RepBase, Perl and C++ modules. It detects interspersed and tandem repeats through sequence similarity comparisons and analyzes repetitive sequences using RepBase Update.	**Advantages:** (1) Censor can automatically classify all known repeats and generate reports. (2) It has a high detection accuracy. (3) It offers online identification services (www.girinst.org/censor/help.html). **Disadvantages:** (1) Highly reliant on homologous databases (RepBase, Dfam, etc.), and cannot discover novel repeats that have not been collected in homology databases. (2) Using BLAST as the alignment algorithm often results in a long run time. (3) The integrity of detection results often depends on the integrity of the homology databases.	^163,225^
Homology-based	RepeatMasker^b^	RepeatMasker is a well-known program that scans DNA sequences for interspersed repeats and low-complexity DNA sequences. It has introduced a new feature that allows the identification of repetitive elements within protein sequences.	**Advantages:** (1) Less false positives and highly accurate and sensitive detection. (2) It does not impose restrictions on the number or length of input sequences. (3) It is versatile and can be utilized to identify repetitive elements in both nucleotide sequences and protein sequences. (4) It can be used to predict genes from masked sequences. **Disadvantages:** (1) Long running times are required when analyzing large-scale genomics. (2) Highly reliant on homologous databases (RepBase, Dfam, etc.), and the integrity of detection results often depends on the integrity of the homology databases.	^226,227^
	LTRharvest^c^	LTRharvest is a de novo detection algorithm used to detect full-length *LTR* elements in large sequence sets based on known features, such as length, distance, and sequence motifs of *LTR* transposons.	**Advantages:** (1) Allows users to make flexible parameter settings. (2) High efficiency, low memory and disk-space consumption. (3) It effectively annotates de novo high-quality, and nearly-full-length *LTR* retrotransposons. **Disadvantages:** (1) It cannot detect partial short *LTR* retrotransposon copies, solo LTRs, and certain nested elements. (2) It is unable to verify the presence of *LTR* retrotransposon-specific open reading frames (ORFs), primer binding sites, or polypurine tracts.	^168,228^
Structure-based	SINE_scan^d^	SINE_scan is a highly efficient structure-based algorithm for predicting *SINEs* in genomic DNA sequences by combining the hallmarks of *SINE* transposition, copy number, and structural signals.	**Advantages:** (1) It is flexible and robust for various purposes of SINE annotation and verification. (2) It provides a more comprehensive detection of *SINEs* in genomes and identifies a substantial number of new *SINEs*. **Disadvantages:** (1) The sensitivity of identification is much lower than other similar tools, such as SINE-Finder. (2) High rates of false discovery.	^173,174^
	RepeatScout^e^	RepeatScout is a de novo identification algorithm that finds repeat families by extending consensus seeds, allowing for a precise determination of repeat boundaries.	**Advantages:** (1) The algorithm runs efficiently. (2) The detection results of the algorithm are pure and accurate. **Disadvantages:** (1) The integrity of the detection results is usually unsatisfactory. (2) The algorithm cannot process more than 1 Gb of the genome at a time. (3) The size change of *l-mer* has a greater effect on the detection results.	^187,229^
De novo	RepLong^f^	RepLong is a de novo method specifically designed for accurately identifying repeats in genomes by constructing overlap networks based on third-generation sequencing (TGS) long reads.	**Advantages:** (1) It can directly obtain repeats only by relying on TGS long reads. (2) Compared with existing de novo detection methods (e.g., RepARK and REPdenovo), it tends to obtain repeats more completely. **Disadvantages:** (1) This algorithm usually consumes vast computing resources (CPU, memory, and disk space) and has a long run time. (2) The detection accuracy of the algorithm is usually unsatisfactory.	^193,230^
	EDTA^g^	The EDTA package is specifically designed to minimize false discoveries in raw TE candidates, enabling the creation of a high-quality, non-redundant TE library for comprehensive whole-genome TE annotations. These annotations contribute to a deeper comprehension of TE diversity and evolution at both intra- and inter-species levels.	**Advantages:** (1) It demonstrates robustness across plant and animal species based on empirical evidence. (2) It is capable of deconvoluting nested TE insertions, which are commonly observed in highly repetitive genomic regions. **Disadvantages:** (1) It can be computationally intensive, requiring significant computational resources and time to process large genome datasets. (2) While it is designed to filter out false discoveries, there is always a risk of false positive or false negative TE annotations. (3) Certain species or specific TE families may pose challenges or have limited support due to variations in TE sequence characteristics and complexities.	^205,231^
Hybrid framework	RepeatMod2^h^	RepeatModeler2 is a package designed to create reference TE libraries applicable to any eukaryotic species. Its capability includes generating libraries that accurately represent the known TE composition of three model species with highly intricate TE landscapes.	**Advantages:** (1) It can create TE libraries that effectively represent the known TE composition of model species with complex TE landscapes. (2) It offers a user-friendly interface, making it accessible to researchers without extensive bioinformatics expertize. **Disadvantages:** (1) It demands substantial computational resources, such as memory and processing power, especially when dealing with large genomes. (2) It heavily relies on existing databases of known TEs, which may limit its effectiveness for species with poorly characterized TE landscapes or novel TE families.	^206,232^

‘Hybrid frameworks’ refer to detection tools that adopt multiple detection strategies, and they usually cannot be clearly distinguished into the above three typical types. ‘EDTA’ is the abbreviation of the extensive de novo TE annotator. ‘RepeatMod2’ is the abbreviation of RepeatModeler2.

^a^https://www.girinst.org/censor.

^b^https://github.com/mmcco/RepeatScout.

^c^https://github.com/oushujun/LTR_retriever.

^d^https://github.com/oushujun/LTR_retriever.

^e^https://github.com/maohlzj/SINEScan.

^f^https://github.com/ruiguo-bio/replong.

^g^https://github.com/oushujun/EDTA.

^h^https://github.com/Dfam-consortium/RepeatModeler.

### Homology-based identification methods

Homology-based methods identify repeats by finding subsequences similar to known repeats, which must rely on algorithms for comparing similarity between sequences, such as the hidden markov model (HMM)-based comparison algorithm, and specific databases, such as RepBase^157^, Dfam^158^, msRepDB^159^, REXdb^160^, and Pfam^161^. RepeatMasker (https://www.repeatmasker.org) is a representation of such tools, which uses Dfam or RepBase as the backend library and RMBLAST (http://www.repeatmasker.org/RMBlast.html) as the aligner. RMBLAST and Dfam are a new aligner and database specially developed by RepeatMasker team for repeat detection based on the existing aligner BLAST^162^ (https://blast.ncbi.nlm.nih.gov/Blast.cgi) and database RepBase (https://www.girinst.org/repbase/). Both RMBLAST and Dfam have become gold standards in the field of repeat annotation. Typical homology-based detection methods also include Censor^163^, TESeeker^164^, Greedier^165^, and T-lex^166^ (Supplementary Table S6). The advantages of homology-based methods lie in their accuracy and the ability to discover families with a small number of copies. Their disadvantage is that they cannot be used to discover new repetitive sequences that are not collected in homology databases. A detailed introduction to homology-based methods can be found in Supplementary Note 7.1.1.

### Structure-based identification methods

Repeats, especially TEs, have specific structures, such as the structure of a protein, or non-coding domains, and differ in the presence and size of the TSD, a short, direct repeat generated on both flanks of a TE upon insertion^167^. Structure-based methods rely on prior knowledge of structural features of known repeats collected in the library and employ a heuristic algorithm to identify repeats in genomes. Typical structure-based identification methods include LTRharvest^168^, MASiVE^169^, MGEScan-LTR^170^, TE-greedy-nester^171^, SINE-Finder^172^, SINE_scan^173^, AnnoSINE^174^, FINDMITE^175^, MUST^176^, detectMITE^177^, MITE-Hunter^34^, MITE-Digger^178^ and, MITE Tracker^179^ (Supplementary Table S7). The advantages of structure-based methods include high detection efficiency and lower false-positive rate, and the detected repeats are easier to verify and classify. Their disadvantages are that they cannot be used to identify repeats whose structural features are unknown or whose structural features cannot be obtained accurately and completely due to the insufficient precision and completeness of the input sequences. Thus, the detection integrity of such methods is often unsatisfactory. Besides, structure-based methods are often designed for a particular class of transposons (e.g., *LTRs*, *SINEs*, and *MITEs*). Therefore their versatility is limited. A detailed introduction to structure-based detection methods is shown in Supplementary Note 7.1.2.

### De novo identification methods

The de novo methods are more flexible than the other two classes of methods because they do not require prior knowledge about the structure or similarity to known repeats^180^, which can also be classified into three categories based on the core technology that each method depends on. The first class of methods includes Repeat Pattern Toolkit^181^, RECON^182^, PILER^183^, LTRdigest^184^, and LongRepMarker^185^, identifying repeats through MSA. The strategy of high-frequency *k-mers* and space seed extension is used in the second category of methods to identify repeats. The sequences to be detected are converted into *k-mers* of a certain length, and *k-mers* whose frequency exceeds a certain threshold are chosen as seeds. Then, the locations of these seeds in the genome are recorded, and the repeats are obtained by performing sequence extensions at both ends of the genome. During the extension process, the detection algorithm always judges whether the extended arrangements are consistent across multiple genome locations. If yes, continue; otherwise, terminate. RepeatFinder^186^, RepeatScout^187^, ReAS^188^, and Generic Repeat Finder (GRF)^189^ are representative of this class of approaches. The third class of methods includes RepARK^190^, REPdenovo^191^, RepAHR^192^, and RepLong^193^, which rely on de novo sequence assembly and community detection in sequence similarity network to identify repeats (Supplementary Table S8). Among these four tools, the first three obtain repeats by performing assembly of high-frequency reads or *k-mers* (Supplementary Fig. S9(a),(b),(c),(d) and (e)). The last method constructs the similarity network by getting the overlaps between long reads, and then use the community discovery algorithm to get the repeats (Supplementary Fig. S9(f)). A detailed introduction to the de novo identification methods is shown in Supplementary Note 7.1.3.

### Tandem repeat and their expansion identification methods

Several tools are available for detecting TRs and their expansions, such as mreps^194^, Tandem Repeats Finder (TRF)^195^, T-REKS^196^, TRASH^197^, EnsembleTR^198,199^, RExPRT^200^, GangSTR^201^, ExpansionHunter^200^, ExpansionHunter De novo^202^, Straglr^203^, and STRling^204^. Among them, mreps excels by detecting all types of tandem repeats in an entire genomic sequence simultaneously. It incorporates a resolution parameter to identify fuzzy repeats with variations within the repeated units. TRF uses sequence alignment and statistics to detect consecutive repetitive motifs. It gives detailed information about identified repeats, including positions, consensus sequence, length, and alignment scores. This information is valuable for genome analysis, gene mapping, investigating structural variations, and understanding repetitive elements in biology and evolution. T-REKS operates by dividing the input sequence into overlapping *k-mer* segments, where k is a user-defined parameter. Then, it employs the k-means clustering algorithm to group similar *k-mers* together, identifying potential TRs. EnsembleTR and GangSTR, developed by the Gymrek Lab, are powerful tools in computational genomics and human genetics. EnsembleTR takes VCF files with TR genotypes for multiple samples and generates a consensus set of genotypes. RExPRT is a machine learning tool used to differentiate pathogenic from benign TR expansions. GangSTR is a tool used for profiling TRs across the genome using short reads. One notable advantage of GangSTR is its ability to handle repeats that exceed the read length. ExpansionHunter and ExpansionHunter De novo are two computational methods developed by Illumina Inc. to locate both known and novel repeat expansions in short-read sequencing data. Straglr is a specialized tool designed to identify and genotype TR expansions using whole genome long-read sequences. STRling is a method for detecting new short TR (STR) expansions from short-read sequencing data, even when no corresponding STR is present in the reference genome.

### Hybrid frameworks

The classification of methods mentioned above is based on the core technology utilized in each method. However, there are certain detection tools like Extensive de novo TE Annotator (EDTA)^205^ and RepeatModeler2^206^, which employ multiple existing detection algorithms or strategies to perform repeat annotation. These tools cannot be easily classified into the above-mentioned three categories due to their unique approach that incorporates multiple existing methods for repeat annotation. For example, EDTA incorporates various tools, such as RepeatModeler and RepeatMasker, which employ homology-based methods, as well as TransposonPSI. In addition, it incorporates structure-based methods like LTRharvest and LTR_retriever. RepeatModeler2 is another hybrid framework, that utilizes the de novo methods RECON and RepeatScout, along with the Dfam database and the alignment search tool RMBLAST, to identify and model repetitive elements in DNA sequences. Performance comparisons between different repeat detection methods are shown in Supplementary Tables S9–S32 of the Supplementary Note 7.2.

## Automated classification and masking of repeats

**Fig. 5:**
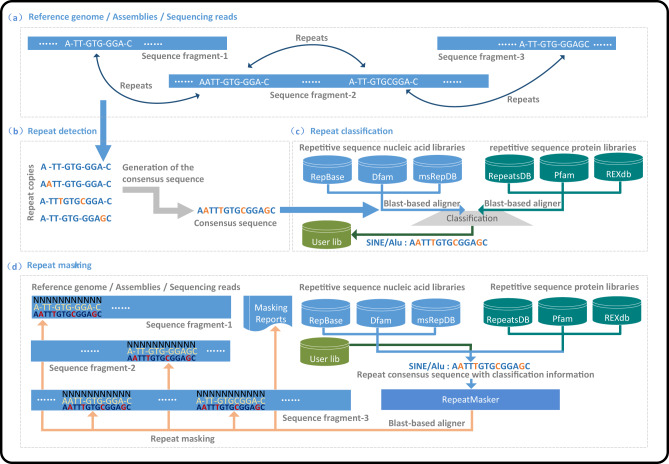
The principle of automatic repeat classification and masking. Sub-graph (**a**): A simple example of the distribution characteristics of repeats in the reference genome, where the black blocks represent chromosomes. Sub-graph (**b**): Principle of repeat detection, where the final sequence composed of colored bases represents the consensus repeat sequence. Sub-graph (**c**): Principle of automatic repeat classification, where black and dark green cylinders represent nucleic acid and protein libraries, respectively. Sub-graph (**d**): Principle of automatic repeat masking. Light green cylinders in Sub-graphs (**c**) and (**d**) represent the user-defined repeat library, and black blocks in Sub-graph (**d**) indicate the sequencing reads from various samples of the same or similar species.

Classification and masking are two necessary steps after the detection stage in the workflow of repetitive DNA sequence analysis. Precise classification and comprehensive masking of repeats are essential for analyzing their critical roles in genomes. The output of the detection stage consists of raw repeat consensus sequences without any information about the type, structure, and function. The purpose of classification is to classify unknown repeats into their main taxonomic branches (e.g., *LTR*, *LINEs*, *SINEs*, *DIRS*, *PLEs*, *MITEs*, *Cryptons*, *Helitrons*, *Mavericks*, Satellites, low complexity sequences, etc.), and to distinguish their structures and functions. The purpose of repeat masking is to mask the repeats in the genome of a specific sequencing sample with the well-classified elements collected in the repeat database using pairwise sequence alignment algorithms, such as nhmmer, cross_match, AB-BLAST/WU-BLAST, RMBLAST, and Decypher, and to report all locations, specific classifications and copy number information of the hit sequences. The principle of repetitive DNA sequence classification and masking is presented in Fig. 5.

### Databases that support automated repeat classification and masking

An accurate and comprehensive repeat database is essential for the automated classification and masking of repeats in genomes. Three well-known nucleic acid libraries, RepBase, Dfam, msRepDB, and three famous protein libraries, RepeatsDB, REXdb, and Pfam, have been proposed to support the automated classification and masking of repeats. RepBase (https://www.girinst.org/repbase/) is a database of prototypic sequences representing repetitive DNA from different eukaryotic species, which currently contains more than 38,000 sequences of different families. Dfam (https://www.dfam.org/releases/Dfam_3.5/) database is an open collection of TEs and genome annotations, which currently houses 285,542 TE models across 595 species and incorporated into the new version of RepeatMasker. msRepDB (https://msrepdb.cbrc.kaust.edu.sa/pages/msRepDB/index.html) is the most comprehensive multi-species repeat database, which currently contains TEs of more than 84,000 species. RepeatsDB (https://repeatsdb.bio.unipd.it/) collects protein structures of annotated TRs, which provides users with the possibility to access and download high-quality datasets either interactively or programmatically through web services. Pfam (http://pfam.xfam.org/) is a database of protein families, which contains many protein families, each of which is represented by MSAs and HMMs. REXdb (http://repeatexplorer.org/?page_id=918) is a reference database of TE protein domains employed in the repeat analysis tools RepeatExplorer2^17^ and DANTE^207^, which are available on the Galaxy server (https://repeatexplorer-elixir.cerit-sc.cz/). A detailed introduction to repeat databases is shown in Supplementary Note 7.3.2. A performance comparison of the databases is presented in Supplementary Tables S33–S41.

### Automated repeat classification methods based on homology searching

The goal of classification is to classify unknown repeats into their main taxonomic branches, which usually refers to the classification of TEs (Fig. 5(a), (b) and (c)). Some methods are proposed based on manually predefined features for automatically classifying TEs, such as TEclass^208^, RepeatClassifier^206^, PASTEC^209^, and REPCLASS^210^. Homology-based searching and structural features of TEs (e.g., TSD, TRs, tRNA, poly-A signals, SSR, and protein-coding domains) are used in these tools to perform classification (Table 5).

**Table 5:** Introduction of typical repeats classification methods.

Method type	Method name	Description/Characteristic	Advantages/Disadvantages	References
Homology-searching based	PASTEC^a^, REPCLASS^b^, TEclass^c^	These methods utilize a homology search approach, such as BLAST, to compare the input sequences with established repeat databases (e.g., Dfam, Pfam, RepBase), in order to identify similar sequences for repeat classification.	**Advantages:** (1) They can accurately compare and classify repetitive elements according to known families and superfamilies. (2) These methods often include repeat masking, which helps reduce the impact of repetitive regions on downstream processes such as genome assembly or gene expression analysis. **Disadvantages:** (1) These methods heavily rely on the availability and quality of reference databases. (2) Balancing sensitivity and specificity can be challenging. (3) The time and computational resources required can limit their practicality for some projects.	^208–210^
Deep Learning-based	DeepTE^d^, TERL^e^	These methods are capable of learning complex patterns and features directly from the data, without relying on predefined rules or databases. This allows them to capture subtle and non-linear relationships, potentially enabling the identification of novel repeat elements.	**Advantages:** (1) Deep-learning models excel in detecting and classifying divergent repeat elements with low sequence similarity by capturing high-level abstract representations from input features. Thus, they have the potential to uncover previously uncharacterized repeat families or variants. (2) Deep-learning models can generalize features and patterns from various genomic data, potentially allowing their transferability across species or genomic contexts. This broadens their applicability to a wider range of organisms. **Disadvantages:** (1) Deep-learning models require substantial amounts of high-quality annotated training data to effectively learn and generalize patterns. (2) Training and deploying deep-learning models can be computationally intensive and require substantial computational resources.	^212,213^

^a^http://urgi.versailles.inra.fr/Tools/PASTEClassifier.

^b^https://sourceforge.net/projects/repclass.

^c^https://www.bioinformatics.uni-muenster.de/tools/teclass.

^d^https://github.com/LiLabAtVT/DeepTE.

^e^https://github.com/muriloHoracio/TERL.

For instance, TEclass (http://www.compgen.uni-muenster.de/teclass) uses support vector machine (SVM) and oligomer frequencies to classify TE consensus repeat sequences into DNA transposons and retrotransposons, including LTRs, LINEs, and SINEs. RepeatClassifier (https://github.com/Dfam-consortium/RepeatModeler) is a homology-based classification module designed in the hybrid TE family discovery framework RepeatModeler2, which compares TE families to RepeatMasker repeat protein databases (e.g., Pfam, REXdb) and RepeatMasker repeat nucleic acid libraries (e.g., RepBase and Dfam) using the homology-based aligner BLAST. PASTEC (http://urgi.versailles.inra.fr/Tools/PASTEClassifier) obtains the similarities and structural features of TEs using profile HMMs^211^ and homology-based search algorithms (e.g., tblastx, blastx, and blastn) and then classifies TEs into their respective order. REPCLASS (http://sourceforge.net/projects/repclass/) is a tool that automates the classification of TE sequences using control repeat libraries and structural and homology characterization modules, which can classify accurately virtually any known TR types.

### Automatic repeat classification methods based on machine and deep learning

Convolutional neural networks (CNNs) are automatic and adaptive representation learning and feature extraction algorithms that can be applied to predict unknown sequence profiles or motifs and functional activity discovery without pre-defining sequence features. Some TE classification algorithms are proposed based on CNNs, among which DeepTE^212^ and TERL^213^ are representatives (Table 5).

DeepTE (https://github.com/LiLabAtVT/DeepTE) tra- nsforms sequences into input vectors through a *k-mer* counting strategy, and classifies TEs into superfamilies and orders based on a tree-structured classification process and eight trained models (class model, classI model, LTR model, nLTR model, SINE model, LINE model, classII_sub1 model and domain model). Among these models, class model is responsible for classifying TEs into Class I, Class II_sub1 and Class II_sub2 transposons, and “ClassI model” is to classify TEs into LTR and non-LTR transposons. Moreover, the false classification correction model and distinction algorithm for distinguishing non-TEs and TEs are also integrated into DeepTE. TERL (https://github.com/muriloHoracio/TERL) is a fast and flexible deep CNN-based approach for classifying TEs and other biological sequences, which employs deep CNNs to preprocess and translate one-dimensional nucleic acid sequences (i.e., image-like data of nucleic acid sequences) into two-dimensional space data. TEclass is an automated classification algorithm based on machine learning support vector machine (SVM). The classification obtained using TEclass is very sparse relative to the overall TE classes, usually only including DNA transposons, LTRs, LINEs, and SINEs. Besides, TEclass can only roughly distinguish non-TE sequences, but cannot accurately classify them. Compared with TEclass, TERL can distinguish non-TE sequences and label numerously of unknown types of repetitive sequences in the detection results as corresponding non-TE types, which greatly improves the accuracy of non-TE sequence identification. In addition, TERL has excellent scalability and can be executed seamlessly in GPUs, greatly improving the efficiency of data processing.

### Automated masking of repeats

Repeat masking is also a vital step in the pipeline of genome repeat analysis (Fig. 5(D)). Three steps of detection, classification, and masking are integrated into some hybrid repeat detection frameworks, such as RepeatMasker, RepeatModeler, and LongRepMarker, to obtain classified TEs (e.g., *LTRs*, *LINEs*, *SINEs*, etc.) and masking reports (e.g., the length occupied, coverage ratio, and location of each TE in the genome). As described, RepeatMasker (https://www.repeatmasker.org/) is a robust detection and masking framework based on homology searching. The input of RepeatMasker are the genome to be annotated and a standard repeat library, such as the RepBase or Dfam. During the masking process, RepeatMasker aligns the well-classified TEs collected in the repeat library to the sequences of the genome one by one, records the length occupied, coverage ratio, and location of each TE in the genome, and generates a masking report. Performance analyzes of automated repeat sequence classification and masking methods are shown in Supplementary Tables S42–S46.

## Discussion

In this section, we summarize the challenges and solutions in the research field of genomic repeat detection and annotation, as well as future development trends.

Since not requiring prior knowledge, the de novo methods are more flexible and valuable than the homology-based and structure-based methods. However, developing advanced de novo algorithms for comprehensive repetitive DNA sequence detection is challenging due to the short length of NGS reads and the high rate of sequencing errors in TGS (Third-generation sequencing) reads. A hybrid strategy combining short and long reads is currently the most effective way to achieve the above goals. However, before implementing the hybrid strategy, we need to obtain multiple sequencing data, such as NGS reads, TGS reads, and even 10× genomic reads, of the same sample in advance, resulting high detection costs and difficult algorithm design. Therefore, successfully overcoming the impact of sequencing errors in TGS reads and directly carrying out high-precision and ultra-complete repeat detection using the increasing number of high-quality TGS reads will become a research focus in the future. Furthermore, the variation of TRs is closely related to the emergence of complex diseases, such as cancers, neurological disorders, and autism. However, there has not been much progress in the development of algorithms for the detection of TRs and their expansions. Databases containing TRs of multiple species are also very scarce. Therefore, researching superior identification methods for TRs and complete TR databases is of great significance in exploring their biological functions in genomes, which is another important research focus in the future.

Several automatic repeat classification methods have been proposed based on machine and deep Learning. These methods all benefit from SVM and CNNs and perform better than traditional methods in some aspects. However, the completeness of the classification is very limited. For example, TEclass can only classify TEs into the following four classes: DNA transposons, *LTR*, *LINE*, and *SINE*, and its classification results tend to have high false-positive rates. Moreover, DeepTE uses CNNs to classify unknown TEs by converting sequences into input vectors based on *k-mer* counting, which can be used to distinguish TEs and non-TEs with relatively low false-positive rates. Both TEclass and REPCLASS cannot distinguish between TEs and other non-TEs, so DeepTE is superior to them. Nevertheless, DeepTE is also not perfect. First, the completeness of its classification remains unsatisfactory. Second, DeepTE is not specifically designed to classify nested TE, and the databases it depends on do not include annotations for nested TEs. Deep neural networks (DNNs) have great application potential in automated repeat classification. However, current methods did not maximize the advantages of DNNs. Therefore, developing superior DNNs and models for more comprehensive and accurate repeat classification is one of the main research focuses for the future.

TEs carry *cis*-regulatory sequences that can alter gene regulatory networks through redistributing transcription factor binding sites and developing novel enhancer activities. Its abnormal expression is closely related to many complex diseases, such as cancers. However, the role of TEs in cell-type heterogeneity and biological processes has not been fully revealed, and research in this field is still in its infancy. With the rapid development of single-cell technologies, scRNA-seq has become an efficient method for observing cell activity, which can be used to analyze gene-centric and TE expression accurately. Therefore, a future research focus is to quantify TE expression and explore the role of TEs in the pathway and mechanism of complex diseases at the single-cell level.

## Conclusion

Repetitive DNA sequences play an indispensable role in the physiological activities of organisms, and they comprise almost half of the human genome. Repeats in genomes can be divided into TEs and TRs. TEs can result in mutations, altered gene expression, chromosome rearrangement. etc., which are related to many diseases, such as cancers, genetic disorders, autoimmune diseases, and metabolic disorders. TRs, especially STRs, are highly variable, which can accelerate the gene expression evolution and generate sufficient variability that allows a rapid evolution and adaptation of organisms, and play a vital role in the structural stability of genetic materials and regulate gene expression, causing various disorders. Due to a lack of sufficiently advanced detection technologies, the role and effect of repeats in genomes, especially the human genome, have been underestimated. We believe that this review will be helpful in the understanding of repeats in genomes and provide guidance for repeat annotation (detection, classification, and masking) and in-depth exploration of its association with human diseases.

### Reporting summary

Further information on research design is available in the Nature Portfolio Reporting Summary linked to this article.

### Supplementary information

Supplementary Information

Reporting Summary

## Data Availability

The reference genomes of six species: Homo sapiens (GCF_000001405.39), Gallus (GCF_016699485.2), Mouse (GCF_000001635.27), Drosophila melanogaster (GCA_018903765.1), Glycine max (GCA_000004515.5) and Leafcutter ant (GCA_000204515.1) are downloaded from the NCBI website (https://www.ncbi.nlm.nih.gov/). Five groups of NGS short reads: Leafcutter Ant (ERR034186, https://www.ncbi.nlm.nih.gov/), D.melanogaster (SRR350 908, https://www.ncbi.nlm.nih.gov/), Mouse (ERR2894257, https://www.ncbi.nlm.nih.gov/), Human-chr14(https://gage.cbcb.umd.edu/) and HG003_24149_father (D2 S2 L001 R1 001, ftp://ftp-trace.ncbi.nlm.nih.gov/giab/ftp/data), three groups of barcode linked reads (HG003_24149_father, HG004_NA24143, and HG002_NA24385_son, ftp://ftp-trace.ncbi.nlm.nih.gov/giab/ftp/data), three groups of CCS long reads (HG003_24149_father, HG004_NA24143_mother and HG002_NA24385_son, ftp://ftp-trace.ncbi.nlm.nih.gov/giab/ftp/data), and four groups of PacBio long reads (dro_100k, human_100k, dmel_filtered and human_polished, https://github.com/ruiguo-bio/replong) are used to evaluate the performance of each tool in this study.
