# Supplementary material for: Repetitive DNA sequence detection and its role in the human genome
Source: Commun Biol. 2023 Sep 19;6:954. doi: 10.1038/s42003-023-05322-y (PMC10509279; doi:10.1038/s42003-023-05322-y)
Supplement: Supplementary file 1 — Supplementary Information [file 42003_2023_5322_MOESM1_ESM.pdf]

Supplementary Materials of “Repetitive DNA sequence detection and its role in the human genome”

Xingyu Liao[1], Wufei Zhu[2], Juexiao Zhou[1], Haoyang Li[1], Xiaopeng Xu[1], Bin Zhang[1] and Xin Gao[1](✉)

1. Computational Bioscience Research Center (CBRC), Computer, Electrical and Mathematical Sciences and Engineering Division, King Abdullah University of Science and Technology(KAUST), Thuwal 23955, Saudi Arabia.
2. Department of Endocrinology, Yichang Central People’s Hospital, The First College of Clinical Medical Science, China Three Gorges University, Yichang 443000, P.R. China.

*Corresponding author: Xin Gao (Email: xin.gao@kaust.edu.sa)

Supplementary Note 1 Glossary table for acronyms/terminologies used in this paper

To improve reader comprehension, we have included a glossary table (**Supplementary Table S1**), which provides detailed explanations for all acronyms and terminologies used in the manuscript.

Supplementary Table S1. Glossary table for acronyms/terminologies used in this paper.

Abbreviation	The corresponding full name of the abbreviation
Repeats	Repetitive DNA sequences
TEs	Transposable Elements
TRs	Tandem Repeats
LTRs	Long Terminal Repeats
LINEs	Long Interspersed Nuclear Elements
L1	LINE-1
L2	LINE-2
L3	LINE-3
SINEs	Short Interspersed Nuclear Elements
Alu	Arthrobacter luteus
HERV	Human Endogenous Retroviruses
VNTR	A variable number tandem repeat
SVA	SINE-VNTR-Alu
DIRS	Dictyostelium Intermediate Repeat Sequence
PLEs	Penelope-Like Elements
MITEs	Miniature Inverted-repeat TEs
MaLRs	Mammalian apparent LTR retrotransposons
ARMDs	Alu recombination-mediated deletions
TERTs	Telomerase Reverse Transcriptases
RTs	Reverse Transcriptases
TIRs	Terminal Inverted Repeat sequences
TSD	Target Site Duplication
YR	Tyrosine Recombinase
CREs	Cis-regulatory DNA elements
ORFs	Open Reading Frames
SNPs	Single Nucleotide Polymorphisms
STRs	Short Tandem Repeats
lncRNAs	long noncoding RNAs
siRNAs	small interfering RNAs
mRNA	messenger RNA
rRNA	ribosomal RNA
rDNA	ribosomal DNA
ASD	autism spectrum disorder
HLA loci	human leukocyte antigen (HLA) super-locus
UTRs	Untranslated regions
ALS	amyotrophic lateral sclerosis
MSA	Multiple Sequence Alignment
HMM	hidden markov model
GRF	Generic Repeat Finder
TRF	Tandem Repeats Finder
EDTA	Extensive de novo TE Annotator
TGS	Third-generation sequencing
NGS	Next-generation sequencing
CNNs	Convolutional neural networks
SVM	support vector machine
GPUs	graphics processing units

Supplementary Note 2 Types, structures and distributions of repeats in eukaryotic genomes

The classes and length distribution of tandem repeats in the human genome are observed in **Supplementary Table S2**. The proportions for the most abundant repetitive element classes in the genomes of *Human*, *Rice*, and *Drosophila* can be found in **Supplementary Fig. S1**. In **Supplementary Table S3**, the focus is on the presentation of types of repetitive sequences, along with their typical families, length distribution, and a brief introduction. The typical structures of retrotransposons, transposons, and tandem repeats are illustrated in **Supplementary Fig. S2**.

Supplementary Table S2. Classes of tandem repeats in the human genome.

Class of TRs in the human genome	Length of TR unit	Length of TR array
Telomeres	∼6 bp	∼10-15 kb
Tandem paralogous		
rDNA	∼43 kb	∼3-6 Mb
Segmental duplications	∼1-400 kb	∼1kb-5Mb
Microsatellites	∼2-6 bp	∼10-100bp
Minisatellites	∼10-100bp	∼100bp-20kb
Satellites		
Alpha satellite	∼171bp	∼0.2-8Mb
Beta satellite	∼68 bp	∼60-80kb
Gamma satellite	∼48-220bp	∼11-121kb
Satellite I	∼17-25bp	∼2.5kb
Satellite II	∼23-200bp	∼11-70kb
Satellite III	∼5bp	∼3.6kb
Satellite IV	∼35bp	∼25-530kb
Macrosatellites	∼100bp-5kb	∼300kb
Megasatellites	∼1-5kb	∼400kb

Supplementary Note 2.1 TEs in the human genome

As described in the introduction, the repetitive sequences in the eukaryotic genome can be classified into two types: interspersed repeats and TRs [1, 2], and the human genome is no exception. The interspersed repeats in the human genome can be divided into three major groups: DNA transposons, non-LTR retrotransposons, and retrovirus-like LTR retrotransposons [3–5] (**Table 1 in manuscript, Supplementary Table S3, and Supplementary Fig. S2 (a), (b) and (c)**).

Supplementary Table S3. Types of repetitive sequences and their families, length distribution and brief introduction.

Type		Order / Superfamily	Length	Description
Scattered repeats	Retrotransposons	**LTR**<br>–*Copia*<br>–*DIRS*<br>–*ERV*<br>–*ERV1*<br>–*ERVK*<br>–*ERVL*<br>–*Gypsy*<br>–*Ngaro*<br>–*Ty1*<br>–*Ty3*<br>–*Bel-Pao*<br>–*Retrovirus*<br>–*Ngaro*	100bp∼25kb	A LTR is a pair of identical sequences of DNA, which occur in eukaryotic genomes on either end of a series of genes or pseudogenes that form a retrotransposon or an endogenous retrovirus or a retroviral provirus. The LTRs are generally 100bp to 25kb long and are involved in all aspects of their life cycle that includes providing promoter sequences and transcription termination signals. All retroviral genomes are flanked by LTRs, while there are some retrotransposons without LTRs. Typically, an element flanked by a pair of LTRs will encode a reverse transcriptase and an integrase, allowing the element to be copied and inserted at a different location of the genome. Copies of such an LTR-flanked element can often be found hundreds or thousands of times in a genome. LTR retrotransposons comprise about 8% of the human genome. The typical structure of LTR is shown in detail in **Fig. S2 (a)**.
		**LINE**<br>–*CR1*<br>–*I*<br>–*RTE*<br>–*I-Nimb*<br>–*Jockey*<br>–*L1-Tx1*<br>–*L2*<br>–*LOA*<br>–*CRE*<br>–*R2*<br>–*L1*<br>–*Penelope*	500bp∼7kb	LINEs are a group of non-LTR retrotransposons that are widespread in the genome of many eukaryotes. They make up around 21.1% of the human genome. LINEs make up a family of transposons, where each LINE is about 7,000 base pairs long. LINEs are transcribed into mRNA and translated into protein that acts as a reverse transcriptase. The reverse transcriptase makes a DNA copy of the LINE RNA that can be integrated into the genome at a new site. The only abundant LINE in humans is LINE1. The human genome contains an estimated 100,000 truncated and 4,000 full-length LINE-1 elements. Due to the accumulation of random mutations, the sequence of many LINEs has degenerated to the extent that they are no longer transcribed or translated. Comparisons of LINE DNA sequences can be used to date transposon insertion in the genome. The typical structure of LINE is shown in detail in **Fig. S2 (a)**.
		**SINE**<br>–*5S*<br>–*tRNA*<br>–*Alu*<br>–*U*<br>–*ID*<br>–*MIR*<br>–*B1*<br>–*B2*<br>–*7SL*<br>–*B4*	100bp∼700bp	SINEs are non-autonomous, non-coding transposable elements (TEs) that are about 100 to 700 base pairs in length. They are a class of retrotransposons, DNA elements that amplify themselves throughout eukaryotic genomes, often through RNA intermediates. SINEs compose about 13% of the mammalian genome. SINEs are present in many species of vertebrates and invertebrates, SINEs are often lineage specific, making them useful markers of divergent evolution between species. Copy number variation and mutations in the SINE sequence make it possible to construct phylogenies based on differences in SINEs between species. SINEs are also implicated in certain types of genetic disease in humans and other eukaryotes. The typical structure of SINE is shown in detail in **Fig. S2 (a)**.
		**DIRS**<br>–*DIRS*<br>–*Ngaro*<br>–*VIPER*	100bp∼700bp	The DIRS order represents a structurally diverse group of retrotransposons that contain a tyrosine recombinase (YR) gene instead of an INT and do not produce TSDs. DIRSs can be further classified into superfamilies like DIRS, Ngaro, and VIPER. The typical structure of DIRS is shown in detail in **Fig. S2 (a)**.
		**PLE**<br>–*Penelope*<br>–*Neptune*<br>–*Athena*	100bp∼700bp	PLEs are widely distributed from amoebae and fungi to vertebrates, but not in mammals. Very few of them have been detected in plants so far. PLEs are composed of a single ORF that codes for some domains, including the reverse transcriptase (RT) and endonuclease (EN). The typical structure of PLE is shown in detail in **Fig. S2 (a)**.
	Transposons	**MITE**<br>–*hAT*<br>–*Mutator*<br>–*PIF*<br>–*Tc1/Mar*<br>–*PIF/Har*<br>–*CACTA*	50bp∼500bp	MITEs are generally short elements (50 to 500 bp) with terminal inverted repeats (TIRs; 10–15 bp) and two flanking target site duplications (TSDs), which exist within the genomes of animals, plants, fungi and bacteria. Like other transposons, MITEs are inserted predominantly in gene-rich regions and this can be a reason that they affect gene expression and play important roles in accelerating eukaryotic evolution. The typical structure of MITE is shown in detail in **Fig. S2 (b)**.
		**Helitron**<br>–*Aie*<br>–*AthE1*<br>–*Atrep*<br>–*Basho*	< 500bp	Helitrons are the eukaryotic rolling-circle transposable elements which are hypothesized to transpose by a rolling circle replication mechanism via a single-stranded DNA intermediate. Helitrons seem to have a major role in the evolution of host genomes. The typical structure of Helitron is shown in detail in **Fig. S2 (b)**.
		**Crypton**	< 500bp	Cryptons represent a unique class of DNA transposons using tyrosine recombinase (YR) to cut and rejoin the recombining DNA molecules. The typical structure of Crypton is shown in detail in **Fig. S2 (b)**.
		**Maverick**	< 500bp	Mavericks, a novel class of giant transposable elements widespread in eukaryotes and related to DNA viruses. The typical structure of Helitron is shown in detail in **Fig. S2 (b)**.
Tandem repeats		**Satellite**<br>–*macro*<br>–*telomeric*<br>–*5S*	150bp∼500bp	Satellite DNA consists of very large arrays of tandemly repeating, non-coding DNA. Satellite DNA is the main component of functional centromeres, and form the main structural constituent of heterochromatin. The typical structure of Satellite is shown in detail in **Fig. S2 (c)**.
		**Minisatellite**	10bp∼100bp	Minisatellites consist of repetitive, generally GC-rich, motifs that range in length from 10 to over 100 base pairs, which occur at more than 1,000 locations in the human genome and they are notable for their high mutation rate and high diversity in the population. The typical structure of Minisatellite is shown in detail in **Fig. S2 (c)**.
		**Microsatellite**	2bp∼10bp	A microsatellite is a tract of repetitive DNA in which certain DNA motifs (ranging in length from one to six or more base pairs) are repeated, typically 5–50 times. The typical structure of Microsatellite is shown in detail in **Fig. S2 (c)**.

′LTR′ is the abbreviation of Long Terminal Repeat, ′LINE′ is the abbreviation of Long Interspersed Nuclear Element, ′SINE′ is the abbreviation of Short Interspersed Nuclear Element, ′DIRS′ is the abbreviation of Dictyostelium Intermediate Repeat Sequence, ′PLE′ is the abbreviation of Penelope-Like Elements and ′MITE′ is the abbreviation of Miniature Inverted-repeat Transposable Element.

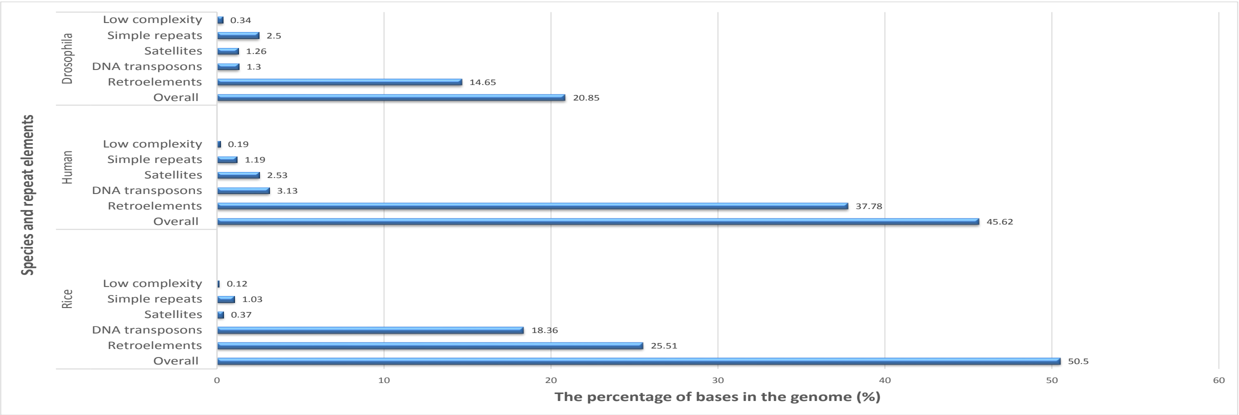

Supplementary Figure S1. The proportions of the most abundant repetitive element classes in the genomes of Human, Rice, and Drosophila are depicted. The X-axis represents the percentage of masked bases in the genome, while the Y-axis represents the species and repetitive elements. The category ′Overall′ represents all types of repetitive sequences, ′Retroelements′ represents the retroposon elements, ′DNA transposons′ represents the DNA transposon elements, ′Satellites′ represents the satellite DNA, ′Simple repeats′ represents the trinucleotide repeats, microsatellites, and minisatellites, and ′Low complexity′ represents the amino acid sequences that contain repeats of single amino acids or short amino acid motifs.

Supplementary Note 2.1.1 DNA transposons Also known as autonomous and Class II transposons, DNA transposons can move autonomously across the genome through the ′cut and paste′ mechanism without the involvement of RNA intermediaries [6]. The presence of TIRs characterizes DNA transposons, which means that TIR sequences are complementary to each other at the left and right ends of the DNA transposon. The general structure of DNA transposons is presented in **Supplementary Fig. S2 (b)**.

The proportion of DNA transposons in the human genome is only about 3% [7] (**Supplementary Fig. S1**), so the interspersed repeats in the genome comprise retrotransposons (RNA transposons). For example, the proportion of retrotransposons in the human genome exceeds 37%. Additionally, DNA transposons are considered DNA fossils, as no family of them currently remains active in most mammals [6, 8]. With no active family, DNA transposons no longer affect the function of the human genome, so they are usually not

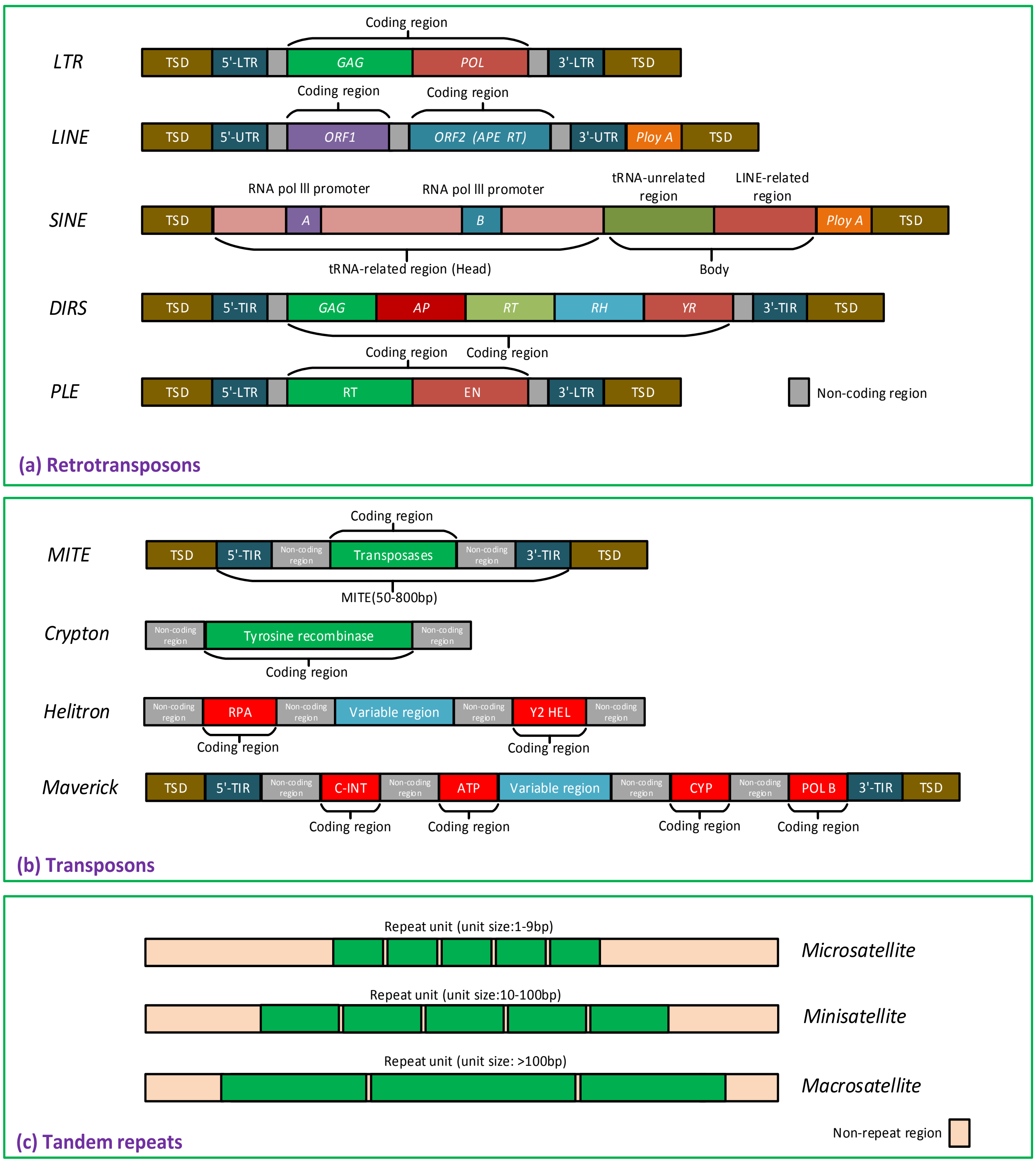

Supplementary Figure S2. Typical structures of Retrotransposons, transposons, and tandem repeats. ′LTR′ is the abbreviation of Long Terminal Repeat, ′LINE′ is the abbreviation of Long Interspersed Nuclear Element, ′SINE′ is the abbreviation of Short Interspersed Nuclear Element, ′DIRS′ is the abbreviation of Dictyostelium Intermediate Repeat Sequence, ′PLE′ is the abbreviation of Penelope-Like Elements and ′MITE′ is the abbreviation of Miniature Inverted-repeat Transposable Element. Sub-graph(a) shows the typical structure of retrotransposons, sub-graph(b) shows the typical structure of transposons, and sub-graph(c) shows the typical structure of tandem repeats. The types of repetitive sequences are summarized in Table S3.

the focus of researchers. Although they are no longer functional, they exist objectively in the human genome. Fossil sequences may contribute to the study of human genome evolution [9], so we describe them in this section.

Supplementary Note 2.1.2 Non-LTR retrotransposons Non-LTR retrotransposons lack LTRs, but contain genes for reverse transcriptases, RNA-binding proteins, nucleases, and sometimes the Ribonuclease H domain [10]. The common structures of non-LTR retrotransposons are presented in **Supplementary Fig. S2 (a)**. In addition, LINE and SINE are the two remaining active super families contained in non-LTR retrotransposons of the human genome, consisting of LINE1, Alu, and SVA, three active subfamilies. Detailed descriptions of the three active subfamilies are presented in the following sections.

As summarized above, the non-LTR retrotransposon families still active in the human genome include LINE-1 (L1), Alu, and SVA. They have all been shown to cause diseases by integrating into human genes. Many studies have suggested that L1 may contribute to human cancers by mutating specific oncogenes or tumor suppressor genes in somatic cells [11]. For example, there is evidence that APC tumor suppressor gene failure is caused by the L1 insertions, which may be an important factor in the development of colorectal cancer [12]. In addition, Alu elements are retrotransposons specifically present in primate genomes that can regulate gene function by providing canonical polyadenylation signals and play a critical role in the primate genomic diversity, causing complex diseases [13]. For instance, many complex human diseases, such as meningococcal disease, venous thromboembolism, obesity, and breast cancer, etc., are related to the structural variants caused by Alu insertions [14]. Currently, SVA is more active than high-copy pseudogenes (e.g., processed ribosomal pseudogenes), and SVA insertions may alter gene expression and cause several human diseases [15]. For example, SVA regulates the expression of related genes whose insertions have been identified as a significant contributor to diseases such as X-linked dystonia-parkinsonism (XDP), Neurofibromatosis type 1, and hemophilia B [16], through mechanisms, such as loss of function mutation, modulation of splicing, and deletions at the site of insertion.

Supplementary Note 2.1.3 Retrovirus-like LTR retrotransposons The common structural organization of retroviruses and LTR retrotransposons is similar [17]. Several LTR retrotransposons have similar open reading frames (ORFs) to those of retroviruses, consisting of the gag and pol (pro) genes and, in some cases, env and other accessory genes. The main difference between retroviruses and LTR is the presence of a functional envelope (env) gene in retroviruses, which is absent or nonfunctional in LTR retrotransposons [18]. The common structures of the retrovirus and LTR are illustrated in **Supplementary Fig. S2 (a)**. No retrotransposable LTR retrotransposons have been identified in the human genome, and no LTR retrotransposon insertions have been collected in the database of human mutations. However, many elements belonging to the young human endogenous retroviruses (HERV) family, such as HERV-K (K denotes a lysine-tRNA-specific primer binding site to initiate reverse transcription), have an individual ORF domain in their structure capable of translation and production of functional proteins [19]. In addition, HERVs are only one type of TE or retroelement found in the human genome. Retroelements and isolated LTRs, as part of molecular evolution, may benefit the host by promoting plasticity and gene expression regulation (i.e., via promoters and cis-regulatory sequences) [20]. The expression of HERV-K envelope transcripts is typically undetectable in normal human breast tissues but is detectable in most breast cancer tissues [21]. Therefore, this expression pattern can be used as a new disease biomarker in clinical diagnosis.

Supplementary Note 2.2 TRs in the human genome

In the human genome, TRs can be divided into four subcategories: microsatellites, minisatellites, centromeric satellites, and telomeric and subtelomeric repeats (**Fig. 1 (f) in manuscript**). The difference between microsatellites and minisatellites is represented in their length and frequency of occurrence. Microsatellites are DNA sequences of less than 10bp units repeated in tandem and are most frequent in the human genome [22]. Minisatellites are tandem repetitions of more than 10 bp units, and their frequency in the human genome is relatively rarer than that of the former [23]. In the human genome, centromeric satellites can be classified into the alpha satellite and Satellite II/III. Among them, the alpha satellite is a high-order TRs, consisting of basic repeat units (A-T rich motifs) of 171 bp in length linked end-to-end [24]. In contrast, Satellite II/III comprises various variations on the ATTCC motif [25]. Telomeric repeats (satellites) are located at the telomeres, consisting of 300 to 8,000 precise CCCTAA/TTAGGG motifs and covering a range of 2 to 50 kb on the end of the chromosomes [26]. Subtelomeric repeats are located in the boundary of 100 to 300 kb between the telomere and the remaining part of the chromosome, consisting of satellite-like sequences [27].

Supplementary Note 2.2.1 Microsatellites Each microsatellite comprises a series of motifs (1 to 5 bp) linked end to end. The common structure of microsatellites is illustrated in **Supplementary Fig. S2 (c)**. Approximately 3% of the human genome comprises of microsatellites [22]. Microsatellites are enriched in the human genome, with more than 600,000 distinct microsatellites. The high mutation rates of microsatellites often cause several neurological diseases and cancers. [28].

Supplementary Note 2.2.2 Minisatellites Each minisatellite is typically repeated 5 to 50 times in the genome and consists of motifs with 5 to 64 bp linked end-to-end. Microsatellites can be found in more than 1,000 locations in the human genome, and their high mutation rate is a significant factor in generating population diversity [29]. Minisatellites have been found in association with essential features of the human genome biology, such as gene regulation, fragile chromosomal sites, and imprinting [30].

Supplementary Note 2.2.3 Centromeric satellites Centromeric satellites are TRs distributed around centrioles, primarily consisting of alpha satellites, as shown in **Fig. 1 (f) in manuscript**. Alpha satellites belong to the AT-rich repeat family comprising of 171 bp monomers [31], which are the most abundant higher-order structures comprising the centrioles of the human genome [32]. Alpha satellites are essential for chromosome segregation and centromere formation and function during cell division in the human genome [33].

Supplementary Note 2.2.4 Telomeric and subtelomeric repeats Telomeric repeats consist of STRs formed by conserved CCCTAA/TTAGGG hexamers spanning 2 to 50 kb [34], located at telomeres, a special region at the ends of human chromosomes. Subtelomeric repeats are also composed of TRs (satellite-like sequences) formed by telomere-derived TTAGGG hexamers. These hexamers are considerably less conserved than telomeres and display differences across chromosomes. Subtelomeric repeats are distributed in the boundary of 100 to 300 kb from telomeres to the remaining part of the chromosomes [27]. The role of telomeric repeats is to keep chromosomes from being degraded and maintain their ability to conduct repair activities that prevent chromosome shortening due to replicating the end of the linear chromosomes [35]. In addition, telomeric and subtelomeric repeats play a key role in meiosis. At the beginning of meiosis, they can assist in the identification and pairing of specific chromosomes [36] that are critical in the later stages of chromosomal recombination between homologs (identical chromosomes in the same genome).

Supplementary Note 3 Challenges of repeats in sequence analysis

Repetitive DNA sequences (repeats) have always presented technical challenges for sequence analysis, such as multiple sequence alignment (MSA), sequencing error correction, SNP and variation detection, and *de novo* sequence assembly. For instance, the assembly of short or long reads is usually affected by the repeats, leading to ambiguous paths in assembly graphs (*de Bruijn*/string/overlap graphs) and eventually forming misassemblies or gaps in generated contigs (**Supplementary Fig. S3 (a) and (b)**), restricting the downstream applications based on complete sequence assembly [37]. Besides, repeats usually cause ambiguity in MSA, interfering with downstream single-nucleotide polymorphism (SNP) identification, variation detection, and gene expression abundance analysis.

There are two strategies to address the problem of ambiguous paths in assembly graphs caused by repeats: 1) paired-end reads with large insertsizes, and 2) third-generation sequencing (TGS) long reads [38]. Among

them, the paired-end reads with large insertsizes can only be used to resolve ambiguous paths whose sizes are equal to or smaller than the insertsizes (usually only a few kilobase pairs in length) [39]. In contrast, TGS long reads can be used to resolve ambiguous paths of a larger size, as they typically span tens to hundreds of kilobase pairs [40]. Although the TGS long reads have the potential to resolve more extensive ambiguous paths in assembly graphs, their efficacy is also limited. For instance, when the size of ambiguous paths is much larger than the maximum length of the TGS long reads (e.g., more than ten megabase pairs), the TGS long reads are also powerless [41]. In addition, telomeres, subtelomeres, and centrioles, composed of highly complex tandem repeats (TRs), pose significant challenges to *de novo* sequence assembly, and the accurate composition of these particular regions has yet to be obtained so far [42].

Repeats complicate determining where reads are aligned by introducing ambiguity during MSA, potentially reducing the sensitivity of detecting SNPs, indels, and other mutations [43] (**Supplementary Fig. S3 (c)**). Repeats in the genome comprise many highly similar copies, some of which may contain specific variations. Due to the high similarity between copies, they can be aligned with each other during the fault-tolerant MSA. Variations belonging to one copy are aligned with other copies, causing confusion in the alignment and reducing the sensitivity of detecting SNPs, indels, and other structural variations [44].

Furthermore, repeats can also interface with the performance of sequencing error correction. Due to the high similarity between copies, when any one of them to be corrected, all the remaining copies will be aligned with it, causing substantial consumption of computing resources. The error correction process erases the specific subsequences between various copies as sequencing errors, losing some significant SNP and variation information, which is primarily why sequence error correction is not performed in structural variation detection. In general, repeats of the genome negatively affect the downstream applications based on sequence assembly and MSA.

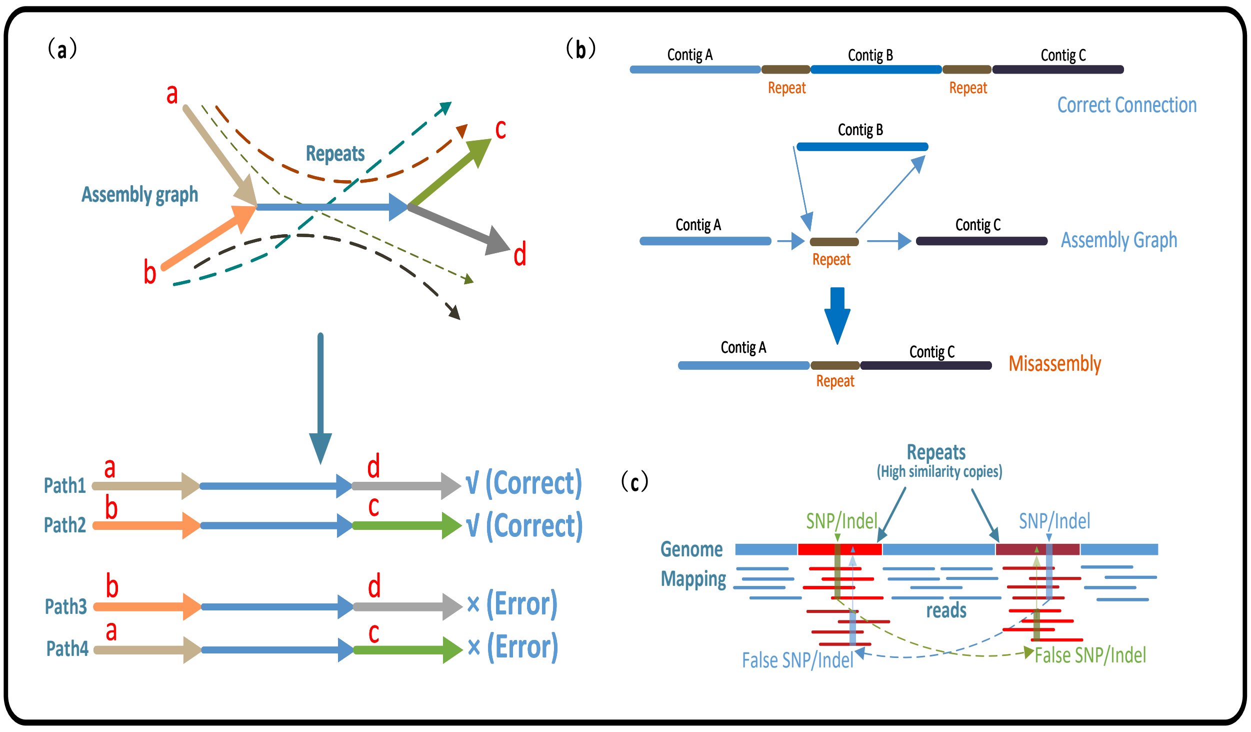

Supplementary Figure S3. Schematic illustration of the computational challenges and negative impact of repeats on sequence assembly and single-nucleotide polymorphism (SNP)/indel detection, respectively.

Supplementary Note 4 Biological Functions of Repeats

Supplementary Note 4.1 Biological functions of transposable elements

The DNA sequences that can move from one location in the genome to another are TEs, which are present in almost all prokaryotic and eukaryotic genomes. The movement of TEs may result in mutations, alter gene expression, induce chromosome rearrangements, and enlarge genome sizes due to increased copy numbers [45]. Thus, they are considered an essential contributor to gene and genome evolution. In addition, TEs have also been recognized as promising candidates for stimulating gene adaptation through their ability to regulate the expression levels of nearby genes. Furthermore, combined with their mobility, TEs can relocate adjacent to their targeted genes and control the expression levels of those genes, depending on the circumstances [46]. Overall, TEs can affect the genome in direct or indirect ways (**Supplementary Fig. S4**).

Supplementary Note 4.1.1 Transposable elements can cause mutations and genetic polymorphisms Many TE families are still active and undergoing constant transposition. Variations are induced when TEs transpose nearby genes and regulatory regions, and these are often rare mutations under purifying selection. For example, an experimental study revealed that the spontaneous insertion of multiple TEs causes more than 50% of all known phenotypic mutants in *D. melanogaster* [47]. Another experimental study found that approximately 10% to 15% of inherited mutant phenotypes in the mouse genome are caused by the autonomous activity of a family of persistently active LTR retransposons [48]. Furthermore, in another study [49], the researchers found that the average difference between any two human haploid genomes is caused by approximately 1,000 TE-dominated insertions, primarily from the L1 (LINE-1) or Alu families.

Supplementary Note 4.1.2 Transposable elements can regulate gene expression and activity repression The TE transposition is an essential factor in gene expression variation, often resulting in extreme gene expression changes much more significantly than those produced by rare SNPs [50]. Involvement in gene expression regulation is another crucial function of TEs in the human genome. There are two primary mechanisms by which TEs regulate gene expression. First, they provide cis-regulatory sequences in the genome with intrinsic regulatory properties for their expression, making them potential regulators of host gene expression. Second, TEs can encode regulatory RNAs. A growing number of studies have demonstrated that their sequences are found in most miRNAs and long noncoding RNAs (lncRNAs), implying that these RNAs are derived from TEs [51]. Moreover, TEs can be activated or repressed under stress conditions. In some cases, the repression of TEs occurs after the initial activation [52]. For instance, to suppress TEs activity, host cells have developed a variety of mechanisms, including epigenetic pathways, such as DNA methylation and histone modifications.

Supplementary Note 4.1.3 Transposable elements can associate with genome rearrangement In reality, TEs can be associated with genome rearrangement through various mechanisms, such as *de novo* TE insertion, TE insertion-mediated deletion, and homologous recombination between them [53]. These rearrangements increase the genomic difference between genomes, and some specific rearrangements may lead to complex diseases [54]. For instance, the expression of retrotransposition-competent TEs may result in additional insertions, which may affect the expression or function of genes [4] and trigger chromosome rearrangements through an ectopic recombination between repeated copies of a TE, causing mutations [55], resulting in several complex diseases, such as cancer [56], Alzheimer's disease [57], and autoimmune and neurological disorders [58]. The specific relationship between TEs and complex disease is discussed in **Section 6**.

Supplementary Note 4.1.4 Transposable elements can act as insertional mutagens in germline and somatic cells Mobile elements, such as L1, Alu, and SVA, are in charge of novel germline insertions, which may lead to genetic illness. For instance, a study has revealed that over 120 independent TE insertions are essential contributors to human diseases [59]. The germline transposition rate for the Alu element in humans is about 1 in 21 births [60], while the corresponding value for the L1 element is about 1 in 95 births [61]. Historically, TEs have generally been considered transcriptional silencing in somatic cells. However, evidence indicates that active TEs are also present in the somatic cells of various organisms. As an illustration, the expression and transposition of the L1 element have been identified in several somatic contexts, such as early embryos and specific stem cells [62]. Human cancers have also exhibited somatic activity, with tumors able to pick up hundreds of additional L1 insertions.

Supplementary Note 4.1.5 Transposable elements can drive key coding and non-coding RNAs According to mounting evidence, TE insertions may serve as the building blocks for forming protein-coding genes and non-coding RNAs that can carry out the crucial physiological functions of cells [63]. For example, Rag1 and Rag2 are spectacular examples of deeply conserved TE-derived genes that activate V(D)J somatic recombination in the immune system of vertebrates [64]. As another example, based on a mixed lncRNA annotation from RNA sequencing and GENCODE (a scientific project in genome research and part of the ENCODE scale-up project), a study estimated that 41% of lncRNA nucleotides are derived from TEs, and the majority of lncRNAs (about 83%) contain at least one TE fragment [65].

Supplementary Note 4.1.6 Transposable elements can alter transcriptional networks and conduce to cis-regulatory DNA elements Cis-regulatory DNA elements (CREs) are regions of non-coding DNA that regulate the transcription of neighboring genes. In addition, CREs are vital components of genetic regulatory networks. Some TEs have evolved into CREs, whose function is to mimic host promoters, enabling them to recruit host-encoded factors driving their selfish transcription [66]. For instance, due to innate and adaptive immune responses, the immune system can protect organisms from pathogens and foreign substances. During evolution, TEs can establish or modify transcriptional networks as signaling molecules that regulate DNA elements and the immune system [67].

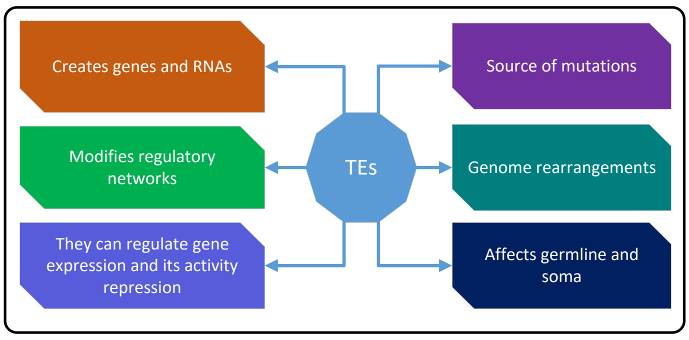

Supplementary Figure S4. How TEs affect the genome. TEs can directly or indirectly affect the genome through some specific mechanisms.

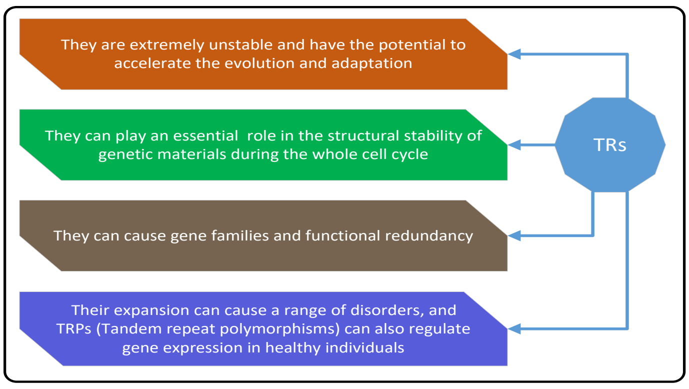

Supplementary Figure S5. How TRs affect the genome. Similar to TEs, TRs can also affect the genome in specific ways.

Supplementary Note 4.2 Biological functions of tandem repeats

Tandem repeats (TRs) are common features of both prokaryote and eukaryote genomes. For example, more than one million distinct TRs are contained in the human genome, many of which are highly polymorphic in sequence composition and copy number. TRs can be found in intergenic regions and in both the non-coding and coding regions of a variety of genes [68–70]. Moreover, TRs occur near or between a series of genes and can affect the structure and function of DNA, RNA, and proteins through specific mechanisms and produce a series of molecular and cellular consequences [71]. As an illustration, many TRs are involved in biological functions in a copy number-dependent manner, and there is evidence that TRs may regulate the expression of nearby genes by altering their copy number [72]. In general, TRs are highly mutable and can be located in exons, introns, or intergenic regions, providing opportunities for the modulation of gene expression, as well as the structure and function of RNAs and proteins [73]. Expanded TRs usually cause various disorders, including autism spectrum disorder (ASD) and cancers (**Supplementary Fig. S5**).

Supplementary Note 4.2.1 Tandem repeats can accelerate evolution and adaptation TR is a sequence of one or more nucleotides that are repeated, and the repetitions are directly adjacent to each other. TRs are also called satellites, which can be further classified into microsatellites or STRs (motif length: 2-6bp), and minisatellites (motif length: 10-60bp), according to the size of the repeated motifs [74]. TRs can occur through various mechanisms. For example, slipped strand mispairing is a mutation process that occurs during DNA replication, which is one explanation for the origin and evolution of repetitive DNA sequences [75]. TRs, especially STRs, are extremely unstable in terms of length, sequence composition, and copy number, with mutation rates typically 10 to 100,000 times higher than in other parts of the genome [76]. These unstable repeats are found in up to 20% of eukaryotic genes and promoters, where they confer phenotypic or functional variability on the cell surface and extracellular proteins and have pathological consequences. Moreover, TRs are also frequently found in genes that control body morphology [77]. For example, compared with synteny blocks, evolutionary breakpoint regions in the human genome contain more base pairs associated with TRs, with AAAT being the most frequent motif [78]. These TRs within evolutionary breakpoint regions have the potential to facilitate and accelerate gene expression evolution and generate sufficient variability to drive the rapid evolution and adaptation of organisms [79].

Supplementary Note 4.2.2 Tandem repeats can play a critical role in the structural stability of genetic materials during the cell cycle Within or around certain specialized chromosomal regions (e.g., centromeres, telomeres, and subtelomeres), TRs may play crucial roles in the structural stability of genetic materials during the cell cycle [36]. For instance, centromeres are the chromosomal domains responsible for the faithful transmission of genetic materials during cell division. An array of tandem repeats, called *alpha*-satellites, is one of the most vital components of centromeres [80]. Nearly all centromeres include *alpha*-satellites, which are necessary for human chromosomal stability. The function of the centromere may be affected by variations in *alpha*-satellites [24].

Supplementary Note 4.2.3 Tandem repeats can result in redundancy of gene families and functions The rRNA-coding genes are tandemly duplicated many times, which are numerous to ensure sufficient DNA templates for the significant buildup of ribosomes needed throughout development [81, 82]. A gene family is a collection of many related genes that typically perform comparable biological tasks. Individual members of clustered gene families are often responsible for achieving specific phenotypes or functions in the overall mission [83].

Supplementary Note 4.2.4 Tandem repeats can regulate gene expression, and their expansion can cause a range of disorders TRs have generous contributions to causing gene expression variation in humans [84], and numerous disorders, such as cancer, ASD, Huntington's disease, various ataxias, motor neuron disease, frontotemporal dementia, and fragile X syndrome, are associated with the expansion of TRs, especially STRs [85, 135, 87]. Recent research indicates that TR polymorphisms can also control gene expression in healthy individuals [88].

Supplementary Note 5 Examples of functionally important repeats in the human genome

Most repeats in the human genome are derived from TEs, which can move within the genome and act as regulatory elements controlling gene transcription, splicing, and genome architecture, potentially causing mutations or altering genome size and structure [89]. In addition, TRs can alter the chromatin structure and affect transcription, leading to gene expression and protein abundance changes, although they represent only a tiny fraction (e.g., microsatellites accounted for only $\sim$3%) of the human genome. In this section, we analyze the role of repeats in the human genome and list several typical examples of their influence on the genome.

Supplementary Note 5.1 Relationship between the hypomethylation of *Alu* and *HERV-K* elements and human aging

There are about 10^6 Alu elements in the human genome, accounting for about 11% of nuclear DNA [90]. These Alu elements occur in about 5% of human alternative exons, interfering with the mechanism of gene splicing [91]. Epigenetic changes and altered gene expression levels may be caused by inserting Alu elements into genes. For instance, the absence of exon 19 during splicing results from the insertion of an Alu element into intron 18 of the human factor VIII gene, which causes severe hemophilia [92]. In addition, HERV-K, which is a family of HERVs associated with malignant tumors of the tests, was inherited million years ago by the genome of the human ancestors that comprise about 8% of the human genome [93].

Alu elements are intrinsic factors leading to DNA damage and instability of the human genome. Further, the hypomethylation of Alu and HERV-K elements also have the potential to cause aging and significantly contribute to the lifespan variation of organisms [94]. The specific mechanism should be that the reduced 5mC content of Alu and HERV-K elements may lead to the reduced efficiency of gene regulation and inappropriate silencing of particular genes, contributing significantly to human aging. For example, experiments designed in one study [95] revealed that a trend of significant reductions in Alu methylation (**Supplementary Fig. S6 (a)**) is observed in centenarians and the offspring of both non-long-lived parents (both $p<0.05$). No change in Alu methylation occurred when the offspring of centenarians are compared with younger controls. When comparing centenarians' offspring to the offspring of non-long-lived parents, the Alu methylation of the former is significantly higher than that of the latter. Another study [96] analyzed the minimum, median, and maximum of the methylation (5mC) levels of Alu and HERV-K elements in different age intervals. Analysis results revealed that, between the ages of 34 and 68, the methylation of the age-associated Alu is significantly lost (r=-0.477, $p<10^{-3}$), and the methylation of HERV-K is lost twice during the 40 to 63 and 64 to 83 age intervals (**Supplementary Fig. S6 (b) and (c)**). These results confirm that age-associated hypomethylation of Alu and HERV-K elements contribute significantly to human aging.

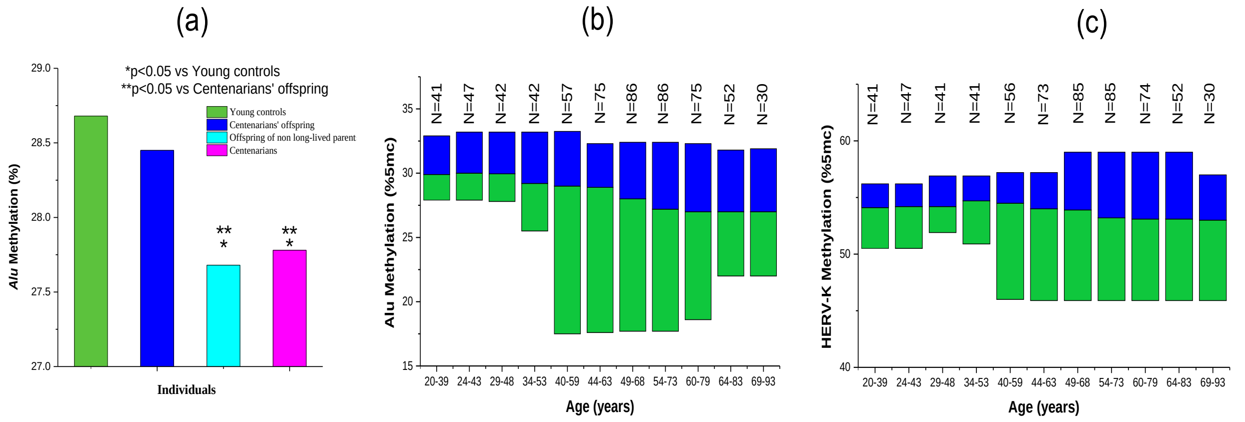

Supplementary Figure S6. Hypomethylation of *Alu* and *HERV-K* elements in relation to human aging. Sub-graph (a): *Alu* methylation statistics of DNA extracted from peripheral leukocytes of young females, female offspring of female centenarians, female offspring of non-long-lived parents, and female centenarians, respectively. In the statistics, the sample number of each group is 21 (n=21) [95]. The asterisks in the figure indicate the degree of significance of the p-value. For example, ″*″ means $p < 0.05$, ″**″ means $p < 0.01$, and ″***″ means $p < 0.001$. Sub-graphs (b) and (c): Minimum, median, and maximum of the methylation (%5 mC) levels of *Alu* and *HERV-K* elements in different age intervals, respectively. In each group, N represents the number of samples tested. The lower and upper boundaries represent the minimum and maximum values after removing outliers. The color boundary in the middle represents the median after removing outliers [96].

Supplementary Note 5.2 Relationship between LINE-1 and gene mutations producing malignant tumors

LINEs are a group of non-LTR retrotransposons and are widespread in the genome of many eukaryotes. LINE-1 (L1) is the only abundant and active LINE in the human genome, and the human genome contains an estimated 100,000 truncated and 4,000 full-length L1 elements accounting for about 17% of the entire genome [97]. Since L1 correlations with disease and immunity, it has become a significant hallmark of several cancers (e.g., ovarian, endometrial, breast, colon, kidney, etc.) and other disorders. The associations between L1 and some complex diseases and its regulatory mechanism are presented in **Fig. 3 in manuscript**. In addition, L1 promotes the occurrence of malignant tumors through three main mechanisms: hypomethylation, aberrant integrations, and high expression of its internal ORF1 and ORF2 domains.

Supplementary Note 5.2.1 Hypomethylation of LINE-1 DNA hypomethylation may lead to chromosomal and genome instability, resulting in genetic heterogeneity. L1 promoter hypomethylation is

an essential biomarker for judging genome-wide DNA hypomethylation. Several studies have demonstrated that L1 promoter hypomethylation is closely associated with the development of gastric, breast, lung, liver, esophageal, prostate, and endometrial cancers. Therefore, L1 promoter hypomethylation is also an essential cancer biomarker. For example, a study [98] has revealed that L1 promoter hypomethylation is significantly associated with low-grade breast cancer (p=0.023), and the median methylation level of L1 in high-grade breast cancer is 62.41%, whereas low grade is 59.08%. Moreover, this study also mentioned that hypomethylation levels of L1 ranged from 70% to 90% in normal tissues and 55% to 60% in tumor tissues of several carcinomas, such as breast and colon cancer [99]. Another related study [100] also revealed that cancer-associated genes are hypermethylated in 70% of colorectal cancer cases compared with normal epithelium, and the hypomethylation of L1 is observed in 90% of colorectal cancer cases. These studies suggest that patients with cancer could be characterized by L1 promoter hypomethylation.

Supplementary Note 5.2.2 Aberrant integration of LINE-1 Numerous studies have demonstrated that many tumor tissues have high levels of L1 activity, and the ′copy-paste′ mechanism of L1s is an essential pathway for the rapid rise of the oncogene copy number, because gene rearrangement mediated by L1s can trigger the rapid amplification of oncogenes. In addition, aberrant integration of L1s can mediate tumor suppressor gene deletion.

For example, the study [101] demonstrated that hypomethylation activates L1s, allowing L1s can insert into the oncogene MYC using a target-triggered reverse transcription pathway, resulting in a specific rearrangement and amplification of oncogenes in breast cancer. Another study [102] proved that L1 mRNA can lead to loss of tumor suppressor genes because it can form facultative heterochromatin in the inactive region or form a RISC complex with pre-mRNA and degrade complementary mRNA through the X inactivation mechanism. Moreover, a related study revealed that the tumor suppressor gene APC in colon cancer is destroyed by the insertion of L1, resulting in the inactivation of the gene [12]. The insertion of L1 into the tumor suppressor gene FGGY promotes cell proliferation and invasion and leads to the occurrence of squamous cell carcinoma of the lung [104]. In addition, a study of genome-wide pan-cancer analysis based on 2,954 cancer genomes across 38 histological subtypes suggested that aberrant integration of L1s may lead to gene rearrangements. Aberrant integration often also includes a breakage–fusion–bridge cycle mechanism. As mentioned in another study, amplification of the CCND1 oncogene in esophageal tumors can be induced by L1 generating a break-fusion-bridge cycle [105].

Supplementary Note 5.2.3 ORF1 and ORF2 domains are highly expressed in LINE-1s The ORF1 and ORF2 domains of L1 are highly expressed in most cancers and thus serve as markers for cancer diagnosis [102]. For example, researchers in the study [106] found that L1 ORF1p protein expression levels are significantly elevated in breast cancer. In addition, researchers in another study [107] have found that L1 ORF1p protein expression is positively correlated with the copy number alteration burden in breast cancer. In some studies of high-grade ovarian cancer, researchers have also detected the high expression of ORF1p and c-Met proteins. For instance, researchers in study [108] have revealed that expression of ORF1p and c-Met proteins is significantly increased in ovarian cancer cells compared to normal cells and peaked in the early stages of ovarian cancer. This phenomenon is related to the loss of TP53 mutation according to another study [109].

A high endonuclease expression causes double-strand DNA breakage, exacerbating DNA damage repair and increasing genomic instability [110], whereas ORF2 can encode a protein with RT and endonuclease activities required for L1 retrotransposition [102]. Therefore, the high expression of ORF2 can cause chromosomal and genomic instability. Furthermore, several studies have found that ORF2p expression is detectable in human colon, prostate, lung, and breast tumors but not in the corresponding normal tissues. For example, an experiment carried out in study [111] revealed that 30% to 100% of all examined cells are reactive in ORF2p-positive tumor biopsies, whereas no immunoreactivity is observed in any of the examined normal tissues (**Supplementary Table S4**). In this experiment, four classes with 74 human adenocarcinoma samples are selected, and the chA1-L1 antibody is used to compare the L1-ORF2p expression levels with those of their healthy untransformed counterparts. These 74 human adenocarcinoma samples comprised ten colon, 54 prostate, six lung, and four breast tissues. The experiment concluded that 96% of the tested samples are chA1-L1 immunoreactive, and in ORF2p-positive tumor biopsies, 30% to 100% of all examined cells are reactive, but immunoreactivity is not found in any normal tissues.

Supplementary Note 5.3 Relationship between SVA retrotransposons and gene expression in the human genome

The SVA (SINE-VNTR-Alu) element has approximately 2,700 to 3,000 copies in the human genome, accounting for 0.13% of the genome. It is the youngest retrotransposon in the human genome and the source of human identity. As modulators, the SVA retrotransposons can be involved in the regulation of gene expression, and the following arguments can support the regulatory function of SVA elements in the human genome, summarized in a previous study [112]. First, there is ample evidence that SVA retrotransposons can regulate gene expression in vitro and in vivo. Second, SVAs are complex high GC structures that affect gene expression in a way that alters the local chromatin structure, Third, polymorphisms of SVAs are essential in determining individual differences and disease risk, as they may lead to allele-specific expression.

More than 60% of SVAs in the human genome are within genes or located in their 10 kb flanking regions [15]. Moreover, SVAs could recruit transcription factors and influence the local chromatin structure, regulating the transcription and expression of nearby genes. As demonstrated by SVAs could make a region accessible or inaccessible to transcriptional machinery. Specifically, how it is regulated depends on the epigenetic marks spread throughout the element [114]. As described in the previous chapters, the hypomethylation of retrotransposable elements has become an epigenetic mark of several diseases, such as cancers. As demonstrated by the regulatory role of L1s in cancer, and changes in epigenetic marks of SVAs, such elements are inappropriately reactivated, possibly leading to the dysregulation of neighboring genes and their associated pathways.

As SVAs are always located in regions of high GC content and gene density, they can generate alternative DNA structures, such as G-quadruplexes (G4), to affect transcription [115]. The promoter regions of more

than 40% of human genes contain one or more G4 sequences [116]. The gene expression in vitro and in vivo can be altered by mutation or stability of the G4 structure [117]. For example, PARK7, a gene closely related to Parkinson's disease, has a full-length SVA called SVA-D, which is a human-specific SVA located approximately 8 kb from the transcription start site [15]. Experiments analyzing the PARK7 gene have demonstrated that the expression of the PARK7 gene in vitro is positively regulated by SVAs in reporter gene assays, and the truncation of SVAs lacking the SINE domain exhibits the strongest enhancer activity (**Supplementary Fig. S7 (a)**).

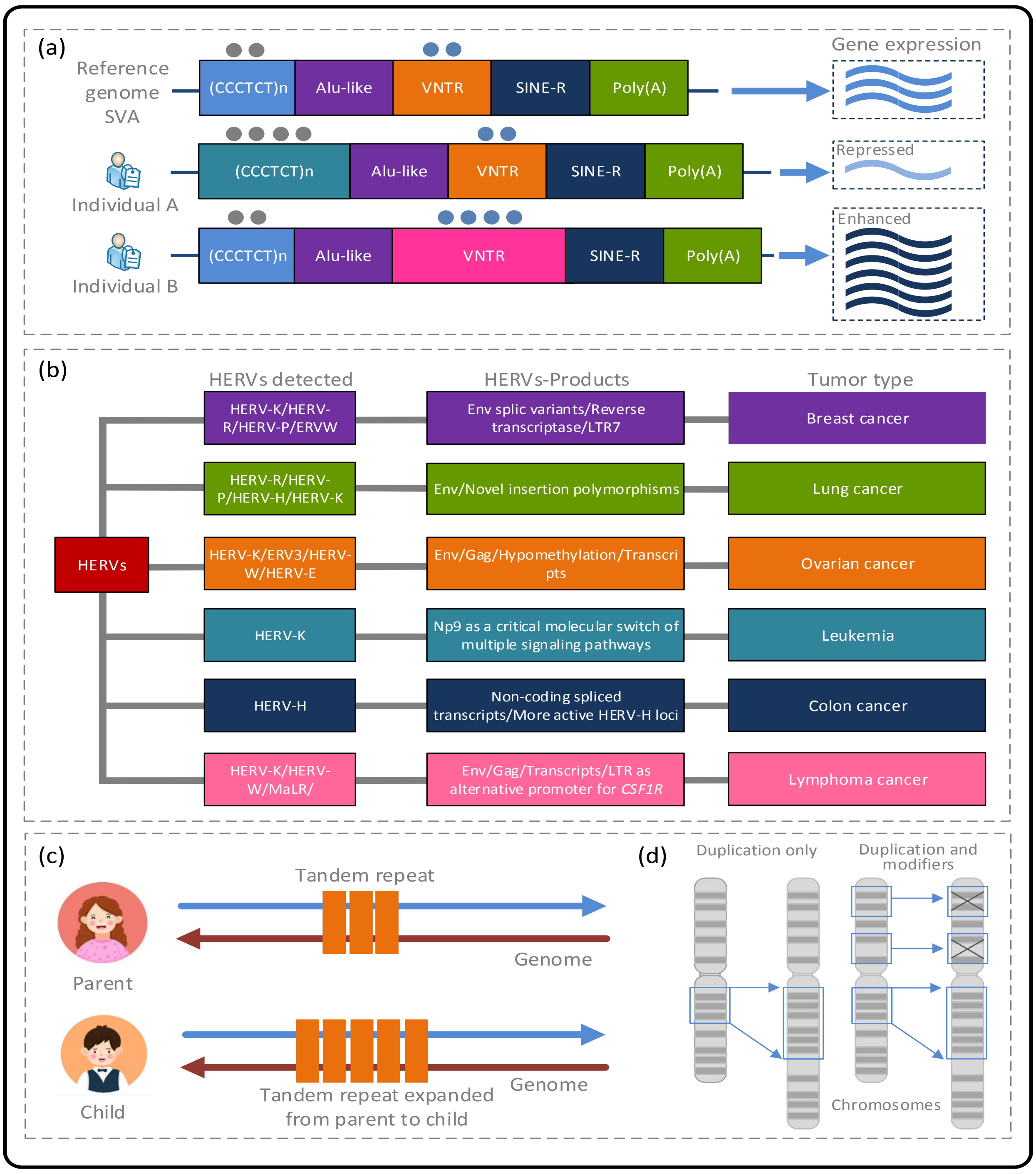

Supplementary Figure S7. The mechanisms of genomic repeats leading to some complex diseases. Sub-graph(a): *SVAs* that can function as regulatory elements and have an allele-like effect on the expression of neighboring genes. The *SVA* at the top has the same sequence composition as the *SVA* in the reference genome. The *SVA* in Individual A is a variant of *SVA* with a longer hexameric repeat domain. The *SVA* in Individual B is another *SVA* variant with a longer *VNTR* domain, acting as an enhancer. Sub-graph(b): Expression of *HERVs* in tumors, in which '*HERV-K* ', '*HERV-H* ', '*HERV-W* ', '*HERV-R* ', '*ERVW* ', '*ERV3* ' and '*MaLR* ' represent the actual *HERVs* detected in the tumor, respectively. Sub-graph(c): The principle of expansions of TRs, in which the TR expanded from parent to child, is suspected of contributing to the genetic etiology of autism spectrum disorder (ASD). Sub-graph(d): Morphology of pathogenic genetic variants. Left: The variation caused by duplication only. Right: The variation caused by duplication and modifiers (e.g., single nucleotide variants, copy number variants, structural variants, and tandem repeat expansions). Some complex diseases, such as cancers, ASD, and neurodegenerative disorders, are usually caused by the second manner.

Supplementary Note 5.4 Relationship between transcriptional activation of HERVs and human cancer

HERVs affect human health and cause disease by encoding proteins, acting as promoters/enhancers or lncRNAs, accounting for about 8% of the human genome [118]. According to their cis-regulatory element activities, HERVs and other types of TEs have been identified as regulatory sequences for many host genes in various cell types throughout mammalian evolution [46]. Several studies have demonstrated that HERV transcripts, proteins, and viral-like particles are present in multiple human cancers.

For instance, researchers found that dysregulation of proto-oncogenes or tumor suppressor genes may result from newly inserted HERVs acting as alternative promoters or enhancers, as revealed in the study [119] that pleiotrophin (PTN) has a HERV type C insertion between its 5' untranslated and coding regions. The

Supplementary Table S4. Immunohistochemical analysis of *L1-ORF2p* expression in healthy and staged cancer tissues using *mAb chA1-L1*.

Tissue[1]	Samples	Grade / Gleason score (pattern)	Num	L1-ORF2p positive cells (%)	Signal intensity
Colon	Normal mucosa		6	0	-
	Transitional mucosa		10	80	+++
	Adenoma	Low grade	8	50	+
		Intermediate	9	80	++
		High grade	6	90	+++
	Adenocarcinoma		1	30	+
			4	50-70	++
			5	80-100	+++
Prostate	Normal / Hyperplasia		20	0	−/±
	PIN		6	90	++
	Adenocarcinoma	6 (3+3)	14	30-90	+
		7 (3+4); (4+3)	23	30-90	+
		8-9 (4+4); (4+5); (5+4)	17	30-90	+
Lung	Normal		8	0	-
	Adenocarcinoma		2	40-60	+
			2	40-60	+
			4	70-95	++ / +++
Breast	Normal		7	0	-
	Invasive ductal carcinoma		4	50-95	++

The signal strength from low to high is: '-' (the signal is the same as the background), '±' (the signal is slightly higher than the background), '+' (the signal is medium), '++' (the signal is high), and '++' (the signal is very high). [1]Staged samples are enrolled from the repositories or biobanks indicated in the Materials and Methods section with their recorded histological information [111].

insertion results in an additional promoter with trophoblast-specific activity and produces HERV and PTN fusion transcripts (HERV-PTN) specifically expressed in human trophoblast cell cultures and trophoblast-derived choriocarcinoma cell lines.

HERVs can also have a direct effect via their proteins in the development of cancers. For example, by inducing cell-cell fusion or epithelial-to-mesenchymal transition, HERV envelope proteins play a critical role in tumorigenesis and development in melanoma, endometrial carcinoma, and breast cancer [120]. Furthermore, HERVs can generate lncRNAs that promote cancer proliferation, motility, and invasion (**Supplementary Fig. S7 (b)**). For example, in the study [121], researchers have found that several HERVs-derived lncRNAs, such as UCA1, SAMSON, and BANCR, are involved in the processes of proliferation, motility, and invasion in bladder cancer and melanoma.

Supplementary Note 5.5 Relationship between tandem repeats and gene expression evolution in the human genome

Due to their intrinsic instabilities, TRs can be mutational hotspots. These highly variable TRs in promoters and other regulatory sequences that control gene expression levels may accelerate gene expression evolution, creating variation in the population and allowing rapid Darwinian evolution and adaptation [77]. For example, single nucleotide (poly-T) polymorphism stretches in the promoter of the human heart disease-related gene MMP3.

A one-nt reduction in ductal size causes increased MMP3 expression and is related to myocardial infarction and aneurysms, whereas a one-nt increase in the allele reduces gene expression and is associated with coronary artery disease. That these sequences are evolving quickly in primates suggests that the MMP3 gene expression and related symptoms may also be evolving rapidly by the mutational hotspot. For another example, researchers in the study [122] explored the genome-wide diversity of TRs in six species, including 83 human and nonhuman great ape genomes, and investigated the influence of TRs on gene expression evolution. The experimental results show that genes containing TRs have higher expression divergence than genes without TRs in their promoters, 3' untranslated regions, introns, and exons. Furthermore, compared to genes with fixed or no TRs in the gene promoters, small polymorphic repeats (1 to 5 bp) have higher expression divergence [123]. This study also highlighted the potential contribution of TRs to the evolution of gene expression in the human genome.

Supplementary Note 5.6 Relationship between tandem repeats and the structural stability of the human genome

The centromeres, telomeres, sub-telomeres, and heterochromatic regions of chromosomes in the human genome comprise highly repetitive TRs, which play crucial roles in influencing the chromosome structure (e.g., alternative DNA structure and packaging) and the stability of genetic materials [124]. For example, telomeres are nucleoprotein structures at the end of each chromosome, and the nucleic acid sequence of telomeres is a highly conserved hexameric (TTAGGG) tandem repeat. The number of hexamer repeats can vary greatly from very few to thousands leading to the lengths of telomeres ranging between 4 to 11 kilobases in humans. Telomere shortening is closely related to the replicative potential of cells and their lifespan. When the telomere length approaches a certain critical level, the cells stop dividing and begin aging and are exposed to apoptosis upon reaching that level.

Homodimers of telomeric repeat-binding factor 1-2 (TRF1 and TRF2), with other components of the Shelterin complex, bind the 90 bp TRFH domain sequence to the spacer, leading to the 3' G-rich single strand forming the T-loop, regulating DNA termination and guarding against the processing of the DNA damage response of the integrity of telomeric repeats [125]. Another crucial chromosomal region is the centromere, comprising highly repetitive TRs. These TRs bind the spindle microtubules during cell division, which is necessary for chromosome segregation [126]. Specific sequence features within alpha satellite sequences are related to chromosomal aneuploidy and to regulating the overall centromeric domain size, implying that centromeres may withstand some size variation to ensure functional fidelity [127].

Supplementary Note 5.7 Relationship between tandem repeats and gene expression regulation in the human genome

Several studies have demonstrated the correlation between TRs and gene expression. For example, more than 700,000 STR loci have been collected in the study [128], but only around 4,500 of them (a relatively small proportion) overlap with coding regions. About 6.8% of these 4,500 loci are located within exons or direct regulators of transcription, such as promoters and enhancers, and the remaining 93.2% are found in intronic and intergenic regions [129]. When TRs are located within introns associated with differential,

Supplementary Table S5. Top candidate tandem repeat loci associated with the autism spectrum disorder.

Gene / region	Risk motifs and their locations (risk motifs)	NUM	PPC	ASDG	DLE
MBOAT7 / intronic	Chr. 19: 54187285–54188613 (AAAG; AAAGGAAG; AAGG)	10	0.006	Known	Novel
FXN / intronic	Chr. 9: 69036648–69037984 (AAG; AAGGAG)	8	0.1	Novel	Known
DMPK / 3′ UTR	Chr. 19: 45769551–45770697 (AGC)	7	0.1	Known	Known
FGF14 / intronic	Chr. 13: 102160822–102162469 (AAGGAG; AAGAGG; AAAGAAGAAG)	7	0	Novel	Novel
CACNB1 / intronic	Chr. 17: 39182673–39183931 (AAGGAGGAG; AAGAAGGAG)	7	0	Novel	Novel
CDON / upstream	Chr. 11: 126063945–126066092 (AAGAGGTGGCAGTATT)	6	0	Novel	Novel
MYOCD / intronic	Chr. 17: 12693129–12694105 (AAAAT)	6	0.1	Novel	Novel
IGF1 / intronic	Chr. 12: 102440998–102442508 (AAG; AAGGAG; AAGAGG)	6	0.1	Novel	Novel
FMR1 / 5′ UTR	Chr. X: 147911368–147912629 (CCG)	6	0	Known	Known
IGF1 / intronic	Chr. 12: 102440998–102442508 (AAG; AAGGAG; AAGAGG)	6	0.1	Novel	Novel
IL1RAPL1 / intronic	Chr. X: 29802527–29803810 (ACACATATGTATACATGTAT; ACACATATGTATATATGTAT)	6	0	Known	Novel

′NUM′: number of samples, ′PPC′: percentage of population controls, ′ASDG′: gene related to ASD, and ′DLE′: type of expansion. The X chromosome loci are excluded from the overall statistical comparisons for the functional analyses. The frequency for 1,612 additional population controls from GTEx consortium 32, and the Mayo Clinic Biobank is used to calculate the percentage of the population controls [135].

and deleterious splicing, a more direct disruption of gene expression may occur, such as disruption of genes by amplification [88, 130]. For example, the shorter length of GT-rich microsatellites in intron2 of the Bromodomain Containing 2 (BRD2) gene can influence alternative splicing and render the BRD2 protein non-functional, dysregulating approximately 1,450 genes under the control of BRD2 [79, 131].

Supplementary Note 5.8 Relationship between tandem repeat instabilities and cancers, autism, and neurological disorders

TR instabilities, especially microsatellite instability, are known to cause cancers, neurogenetic disorders, ASD, and other diseases in humans and are most often present with ataxia as a clinical feature [132]. In addition, TR instability can decrease gene expression and increase disease incidence and tumor aggression (**Supplementary Fig. S7 (c) and (d)**). For example, Lynch syndrome is an autosomal dominant disorder that increases the risk of developing colorectal cancer, endometrial adenocarcinoma, and tumors of the small intestine, stomach, ureter, renal pelvis, ovary, brain, and prostate. Research in study [133] has demonstrated that most (90%) colorectal cancer due to Lynch syndrome have microsatellite instability. In addition, researchers in study [134] have revealed that one neurodegenerative disease in which microsatellite instability contributes to a substantial number of cases is amyotrophic lateral sclerosis (ALS), a rapidly progressive and uniformly fatal motor neuron disease.

Expansion is a significant source of TR instabilities. A study published in the journal *Nature* [135, 136] indicates that TR expansions are rare in normal individuals but are common in patients with ASD, especially near exons and splice junctions and genes related to developing the nervous system, cardiovascular system, or muscle. The gene-associated expansions of TR in people with ASD are much higher than that in siblings without ASD (**Supplementary Table S5**). This study demonstrated that the genetic etiology and phenotypic complexity of ASD are closely related to TR expansions. Furthermore, we listed several essential studies that illustrate the role of repetitive sequences in the human genome in **Supplementary Note 6**.

Supplementary Note 6 Some essential studies illustrate the role of repeats in the human genome

In this section, we listed several essential studies that illustrate the role of repetitive sequences in the human genome. It is worth mentioning that the data, and conclusions given in this section are all citations from the corresponding published literature.

Supplementary Note 6.1 Relationship between LINE-1 and gene mutations

In the study [103], researchers found LINE-1 ORF1p expression to be about twice as high on average in p53 mutant endometrial cancers (Wilcoxon test P = 0.0014, Fig. 4A) and about 50% higher in p53 mutant breast cancers (Wilcoxon test P = 0.011). The correlation between LINE-1 ORF1p expression and CNA burden (average of the absolute value of GISTIC2 estimated CNA across the genome) is highest in endometrial cancer (Spearman $\rho = 0.44$, $P = 3.6 \times 10^{-5}$).

Supplementary Note 6.2 Relationship between LINE-1 methylation and cancers

In the study [113], researchers found LINE-1 methylation levels between the control and lung cancer groups are significantly different in the Mann-Whitney U test ($p < 0.01$). For breast cancer, a significant difference in the LINE-1 methylation in the independent samples are observed ($p < 0.01$)

Supplementary Note 6.3 Overexpression of LINE-1 retrotransposons in autism brain

In the study [137], researchers clearly show for the first time that L1 ORF 1 and 2 mRNA transcripts are significantly elevated in the autism cerebellum relative to carefully matched control samples. As shown in this study, there is a highly significant increase in total RNA and mRNA in both ORF1 and ORF2 in the autism cerebellum, although there is no significant difference in the overall L1 copy number. The remarkably high correlation ($r = 0.95$; $p = 0.0001$) between the expression of ORF1 and ORF2. For full-length insertion to occur, both ORF1 and ORF2 must be expressed. Thus, the coexpression of both ORF1 and ORF2 strongly suggests that the 5′UTR promoter is fully functional since 5′-truncated L1 insertions are transcriptionally incompetent.

Supplementary Note 6.4 Alu insertion variants alter gene transcript levels

In the study [138], researchers measured the effect of the polymorphic Alu on luciferase expression and determined the mechanism by which the Alu alters luciferase expression using a series of ectopic reporter constructs like previous experiments. For two loci, Alu-098 and Alu-103, the effect of the Alu in genomic context (increasing luciferase expression) is recapitulated when the Alu is evaluated independently (adjusted $P < 0.05$, t-test). Further, scrambling the Alu sequence within the genomic context did not increase luciferase expression. Together, this indicates that the effects of Alu-098 and Alu-103 on expression are intrinsic to the Alu.

Supplementary Note 6.5 Alu insertion variants alter mRNA splicing

In the study [139], researchers found that at one locus where detected an effect, a polymorphic Alu element maps 41 bp upstream of exon 33 of the NUP160 gene. NUP160 encodes Nucleoporin 160, a member of the 120-MD nuclear pore complex that mediates nucleoplasmic transport. Exon 33 of this gene is a near constitutive exon, but EST data (JD448821) suggest that it is skipped in a minor transcript isoform; skipping the 143 bp exon 33 would result in a frameshift in the mRNA open reading frame. The 262 bp AluYh3a3 element at NUP160 is oriented antisense with respect to the gene. To determine its effect on exon usage, they tested a 1,743 bp fragment of this locus, both with and without the Alu element present , in the minigene reporter assay. They detect two different splice events with both constructs. Sanger sequencing of the RT-PCR products confirmed that one event includes the NUP160 exon 33, and the other skips the NUP160 exon. Both spliced products are detected with and without the Alu insertion; however, when the Alu is present, the exon is skipped significantly more often, 45.2%, compared to only 20% when the Alu is not present ($P < 0.001$). This indicates that, at least in the reporter assay, this Alu polymorphism has an effect on exon usage; the presence of the Alu promotes exon skipping.

Supplementary Note 6.6 SVA insertion polymorphisms are associated with Parkinson's disease progression

In the study [140], researchers found that SVA_67 at the chromosomal locus 17q21.31 is associated with differential expression of multiple genes, including six in a 1.15 Mb region centered around the SVA RIP. At baseline, when comparing PP and AA genotypes three (PLEKHM1 (FDR $p = 2.38 \times 10^{-6}$), ARL17A (FDR $p = 8.72 \times 10^{-5}$) and CRHR1 (FDR $p = 7 \times 10^{-4}$)) out of the four significantly associated genes are located in this region as are five (PLEKHM1 (FDR $p = 2.40 \times 10^{-9}$), ARL17A (FDR $p = 1.21 \times 10^{-9}$), CRHR1 (FDR $p = 1.47 \times 10^{-8}$), MAPT (FDR $p = 0.001$) and LRRC37A (FDR $p = 0.03$)) out of seven genes whose expression is significantly different when comparing PP to PA genotypes. Extending the analysis to the expression data at 36 months, when comparing PP and AA genotypes of SVA_67, 22 genes are significantly different in expression, 4 of which are located in the 1.15 Mb region (PLEKHM1(FDR $p = 0.008$), ARL17A (FDR $p = 0.006$), CRHR1 (FDR $p = 2.5 \times 10^{-4}$) and MAPT (FDR $p = 0.002$)). These same four genes, as well as two others (LRCC37A (FDR $p = 0.002$) and KANSL1 (FDR $p = 0.06$)) in this genomic region, are also differentially expressed when comparing PP and PA genotypes at 36 months. Four of the genes in this region whose expression is associated with SVA_67 (PLEKHM1, ARL17A, CRHR1, and MAPT) all showed higher expression in individuals with PP genotypes, and the individuals with the lowest expression had an AA genotype. This is in contrast with the levels of expression of LRCC37A and KANSL1, where the opposite pattern is observed.

Supplementary Note 6.7 High Expression of human endogenous retrovirus (HERV)-K and HERV-R Env proteins in various cancers

In the study [141], researchers found that the expressions of HERV-K Env and HERV-R Env protein are significantly higher in tumor tissues compared with normal surrounding tissues in almost all types of tumors. The expression of HERV-K Env is specifically high in breast cancer, melanoma, kidney cancer, prostate cancer, cervical cancer, esophagus cancer, and colon cancer. The expression of HERV-R Env protein is specifically high in melanoma, liver cancer, stomach cancer, ovarian cancer, cervical cancer, esophagus cancer, and colon cancer. However, only osteosarcoma showed weak to moderate expressions of HERV-K Env and HERV-R Env proteins. The relative expression of HERV-K Env and HERV-R Env to the normal surrounding tissues (%Normal) is usually similar in different tumors except breast cancer and melanoma. HERV-K Env protein demonstrated much higher expression than HERV-R Env in breast cancer, whereas HERV-R demonstrated much higher expression than HERV-K Env in melanoma.

Supplementary Note 6.8 Somatic mutations in microsatellites in cancer

In the study [142], researchers compared mutational signatures found in single base substitution (SBS), doublet base substitution (DBS), as well as IDs between the MSI and MSS samples. The PACWG signature analysis detected 49 SBS, 11 DBS, and 17 ID signatures. They compared the fraction of each mutational signature between MSI and MSS samples in CR (colorectal cancer), ST (stomach cancer), and UT (uterine cancer) and found that six SBS signatures (SBS5, SBS15, SBS20, SBS21, SBS26, and SBS44), one ID signature (ID2), and four DBS signatures (DBS3, DBS7, DBS8, and DBS10) are significantly different among the MSI and MSS samples in at least one cancer type (Wilcoxon signed-rank test, q-value < 0.05). Except for DBS3 and DBS8, most of these mutational signatures have been reported to be associated with tumors having defective DNA MMR.

Supplementary Note 6.9 Rare tandem repeat expansions in ASD

In the study [143], researchers found a trend of rare tandem repeat expansions in the enriched gene sets more often in females than in males (odds ratio $= 1.3$; $P = 0.11$) , which may further support the differential genetic loading for males and females in ASD. Consistent with our previous findings for rare pathogenic SNVs (single nucleotide variants) and CNVs (copy number variations), individuals with rare tandem repeat expansions had lower IQ (Wilcoxon test, $P = 0.001$) and Vineland Adaptive Behavioural standard scores (Wilcoxon test, $P = 0.019$) . This provides compelling evidence for the role of rare tandem repeat expansions in ASD-related phenotypes.

Supplementary Note 7 Classification of repeat detection methods

Numerous computational methods for identifying repeats in the genomes have been proposed. They can be draftily divided into the following three categories: homology-based, structure-based, and *de novo* methods (**Supplementary Fig. S8**). Some *de novo* methods, such as EDTA, RepeatModeler2, and LongRepMarker, are hybrid detection frameworks that often integrate multiple detection approaches (e.g., LTRharvest, RepeatScout, RECON, etc.), classification and masking modules to identify various types of repeats (TEs, TRs, low complexity sequences, etc.) in the genome. Therefore, these methods cannot be accurately classified into the above three categories. The above classification of *de novo* methods is roughly performed based on the core technology they depend on.

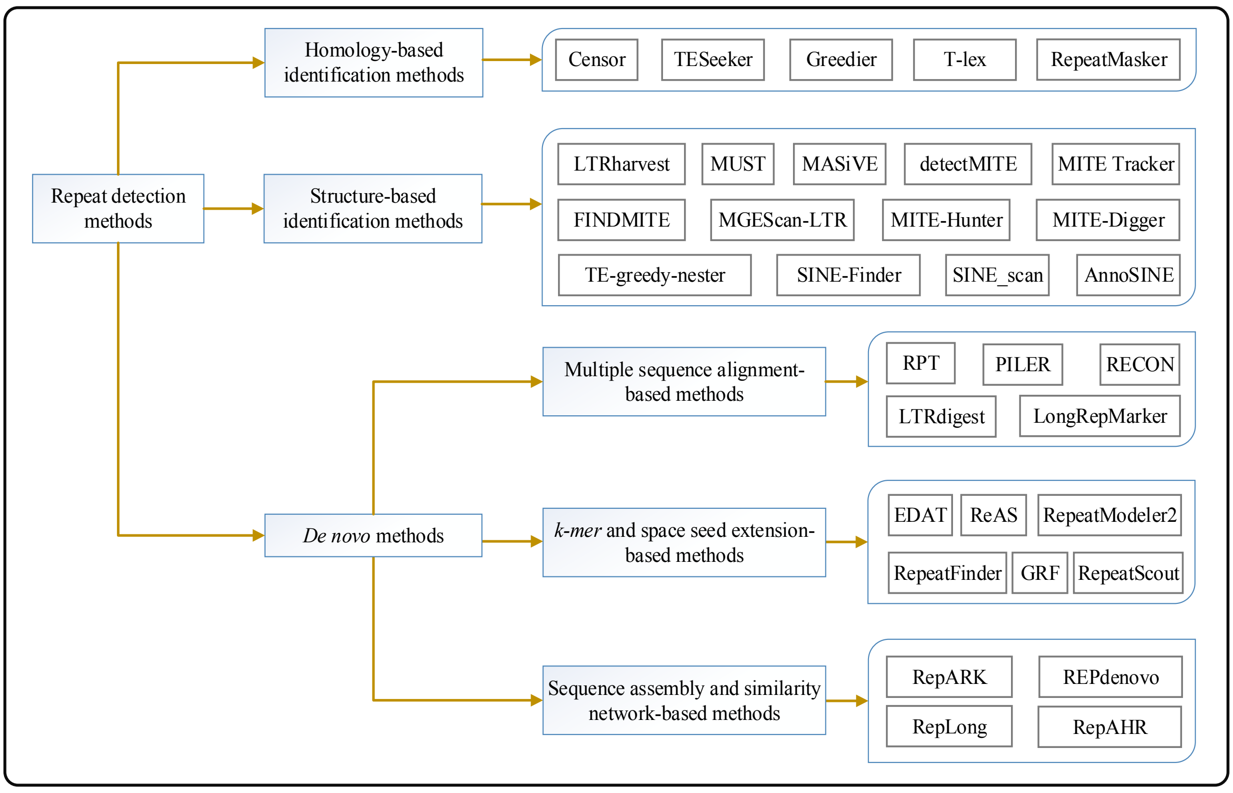

Supplementary Figure S8. Classification of detection methods. Some tools belong to hybrid detection frameworks, such as LongRepMarker, RepeatModeler2, EDTA, and GRF. These tools integrate various detection techniques; thus, accurately classifying them into a specific category is challenging. Therefore, they can only be roughly classified according to the main strategies. EDTA: Extensive *de novo* TE Annotation. GRF: Generic Repeat Finder.

Supplementary Note 7.1 Introduction of typical repeat detection methods

Supplementary Note 7.1.1 Homology-based identification methods Homology-based identification methods identify repeats by finding subsequences similar to known repeats, which must rely on algorithms for comparing homology similarity between sequences, such as the Hidden Markov Model (HMM)-based homology comparison algorithm, and specific databases (e.g., RepBase [144], Dfam [145], msRepDB [146], RepeatsDB [147], REXdb [148], and Pfam [149]). RepeatMasker (https://www.repeatmasker.org) is a representation of such tools, which is based on the Dfam or RepBase library and the alignment algorithm RMBLAST (http://www.repeatmasker.org/RMBlast.html) to perform homology-based similarity searching. Among them, RMBLAST and Dfam are the special alignment algorithm and database developed by the RepeatMasker team based on the existing Basic Local Alignment Search Tool (BLAST) [150] (https://blast.ncbi.nlm.nih.gov/Blast.cgi) and RepBase (https://www.girinst.org/repbase/), respectively. In terms of accuracy, both RMBLAST and Dfam have become gold standards in the field of repeat masking and are used in the background by several repeat identification frameworks for searching and masking. Typical homology-based detection methods also include Censor [151], TESeeker [152], Greedier [153], and T-lex [154].

Among these homology-based methods, Censor uses RepBase as a homologous database. The local alignment and greedy algorithms are used by Greedier to determine embedded repeats effectively. In addition, Dfam and RepBase are used as the homology databases in TESeeker. Further, T-lex is one of the few transposon identifications that can apply large-scale high-throughput strain data and quickly return population frequency estimates for individual TE insertions. The benefits of homology-based methods include their accuracy and the ability to discover families with a small number of copies. Their disadvantage is that they cannot be used to discover new repeats that have not been collected in homology databases. Typical homology-based detection methods are introduced in **Supplementary Table S6**.

Supplementary Note 7.1.2 Structure-based identification methods Repeats, especially TEs, usually have specific structures, such as the structure of a protein or non-coding domains. Furthermore, these repeats differ in the presence and size of the TSD, a short, direct repeat generated on both flanks of a TE upon insertion [155]. Structure-based identification methods rely on prior knowledge of structural features of known repetitive elements collected in the library and employ a heuristic algorithm to identify repeated sequences in genomes. Typical structure-based identification methods include LTRharvest [156], MASiVE [157], MGEScan-LTR [158], TE-greedy-nester [159], SINE-Finder [160], SINE_scan [161], AnnoSINE [162], FINDMITE [163], MUST [164], detectMITE [165], MITE-Hunter [166], MITE-Digger [167] and, MITE Tracker [168].

The LTR retrotransposons are Class I TEs characterized by the presence of long terminal repeats (LTRs) directly flanking an internal coding region, which comprises about 8% of the human genome [4]. Several LTR retrotransposons have similar open reading frameworks (ORFs) to those of retroviruses, consisting of the gag and pol (pro) genes and, in some cases, env and other accessory genes. There are already some tools, such as LTRharvest, MASiVE, MGEScan-LTR, and TE-greedy-nester, specifically designed for the *de novo* LTR retrotransposons detection based on the above structural features. For example, LTRharvest determines the boundary position of LTR by setting multiple filtering steps according to the sequence's structural characteristics (e.g., canonical features like LTRs, TSDs, and distance constraints). Besides, MASiVE is a program used to detect and analyze SireVirus elements that belong to specific LTR transposons in plant genomes based on the structural features of the polypurine tract and primer binding site domains of all LTR-RTNs. Moreover, MGEScan-LTR is also a structure-based LTR detection tool that can be used to identify all types of LTR retrotransposons using approximate string matching, protein domain analysis, and profile HMMs. In addition, TE-greedy-nester is another structure-based method that can be used to identify LTR retrotransposons and their nesting based on a greedy recursive algorithm to mine increasingly fragmented copies of full-length LTR retrotransposons in assembled genomes and other sequence data.

SINEs are non-coding retrotransposable elements amplified by RNA intermediates in copy-and-paste mode, which are small TEs ranging from 100 to 700 bp. SINEs can but do not necessarily have to possess a head, a body, and a tail. The head is at the 5' end of SINEs and is evolutionarily derived from an RNA synthesized by RNA Polymerase III, such as ribosomal RNAs and tRNAs. The body of SINEs possesses an unknown origin but often shares much homology with a corresponding LINE which thus allows SINEs to parasitically co-opt endonucleases coded by LINEs (which recognize certain sequence motifs). Lastly, the 3' tail of SINEs is composed of short simple repeats of varying lengths; these simple repeats are sites where two (or more) short-interspersed nuclear elements can combine to form a dimeric SINE. Several structure-based identification methods, such as SINE-Finder, SINE_scan and AnnoSINE, have been proposed for SINEs identification. Among them, SINE-Finder is a Python script developed to report the targeted identification and characterization of tRNA-derived SINEs from plant genomes based on the structural features of SINEs, such as the motif of 5' TSD, box B, and 3' TSD. SINE_scan is an efficient method to identify SINE elements in the genome based on the hallmark of the SINE transposition (special sequence pattern around the insertion site), copy number, and structural signals (e.g., classification and genome-wide annotation). In addition, AnnoSINE is another accurate and efficient SINE annotation tool, in which the homology search based on the profile HMM and the *de novo* SINE search employing structural features are used to maximize the range of SINE candidates.

MITEs are a special class of DNA transposons inserted predominantly in gene-rich regions, which could be why they affect gene expression and play essential roles in accelerating eukaryotic evolution. The six standard structure-based identification methods for MITE detection are MITE-Hunter, detectMITE, FINDMITE, MUST, MITE-Digger, and MITE-Tracker. Among them, FINDMITE requires the TSD sequence, and users must predefine the minimum and maximum distances between the terminal inverted repeats (TIRs). In addition, MITE-Hunter is a procedural pipeline for identifying MITEs than FINDMITE and MUST, and its output is easier to inspect and classify. In MITE detection, a combination strategy of *de novo* and structural-based approaches is used in the MITE-Hunter and MITE-Digger programs. Both methods cannot detect all MITEs concealed in the genomes, despite successfully reducing false-positive rates in MITE detection. The advantages of structure-based methods include high efficiency and lower false-positive rates of the detected repeats, and their detection results are easier to verify and classify. Their disadvantages are that they cannot be used to identify repeats whose structural features have not been collected in structure databases or whose structural features cannot be accurately and completely obtained due to the insufficient precision and completeness of the input sequences. Therefore, the detection integrity of such methods is often unsatisfactory. Additionally, structure-based methods are often designed for a certain class of transposons (e.g., LTR, SINEs, and MITE), and their versatility is limited. Typical structure-based detection methods are introduced in **Supplementary Table S7**.

Supplementary Note 7.1.3 De novo identification methods The *de novo* methods are more flexible than the other two classes of detection methods because they do not require prior knowledge about the repeat structure or similarity to known repeat sequences [169]. The methods can also be classified into three categories based on the core technology that each method depends on (**Supplementary Fig. S8**). The first class of methods identifies repeats through MSA, including the Repeat Pattern Toolkit (RPT) [170], RECON [171], PILER [172], LTRdigest [173], and LongRepMarker [174]. RPT is designed based on a sequence similarity scoring system, which uses BLAST as an aligner to perform MSAs between genomic sequences. In the processing of RPT , the sequences are grouped using a graph-based single-link clustering algorithm, and each one is considered a vertex in the graph, and two vertices are linked if they overlap by more than a certain threshold. RECON is designed based on extensions to the usual approach of single linkage clustering of local pairwise alignments between genomics sequences. PILER is a *de novo* repeat annotation method that exploits characteristic patterns of local alignments induced by certain classes of repeats, in which the searching procedures are designed to determine repeat elements with boundaries corresponding to individual biological events by finding instances that produce characteristic signatures. LTRdigest identifies and annotates characteristic sequence features of LTR retrotransposons in predicted candidates, which uses several algorithms to create annotations based on user-supplied constraints, and computes the boundaries and attributes of the features that fit the user-supplied model and output. LongRepMarker is a novel framework for repeat identification and classification, which is implemented based on the combination of unique *k-mers*-based MSA and the hybrid assembly of short and long reads. Multiple-alignment unique *k-mers* are used in LongRepMarker to locate repetitive regions accurately, and long sequencing fragments (TGS long reads) are introduced into the assembly process of short paired-end reads to fully restore repeats in the genome.

The methods in the second category are based on the strategies of high-frequency *k-mers* and space seed extension to identify repeats, which convert the sequences to be detected into *k-mers* of a certain length and choose *k-mers* whose frequency exceeds a certain threshold as a seed. During the extension process, these methods obtain the expanded sequences by searching for the locations of these seeds in the genome, performing sequence extensions at both ends of the genome, and always judging whether the extended arrangements are consistent across multiple genome locations. Representative of this class of approaches include the Extensive *de novo* TE Annotator (EDTA) [175], RepeatFinder [176], RepeatScout [177], ReAS [178],

Generic Repeat Finder (GRF) [179] and RepeatModeler2 [180]. EDTA (Extensive *de novo* TE Annotator) is a pipeline for comprehensive and high-quality TE annotation for newly assembled eukaryotic genomes or to expand curated TE libraries, which contains a set of scripts for filtering the output of each program to reduce the overall false discovery rate. In addition, EDTA also can be used to identify nested TE insertions frequently found in highly repetitive genomic regions. RepeatFinder is a new clustering method for analysis of the repeat data captured in suffix trees, which uses a set of *k-mer* tagged sub-strings, traditionally identified by the REPuter [181] search engine, to initialize its hierarchical clustering strategy. RepeatScout is a tool developed for identifying repeats in assembled genomic regions, which builds a library of high-frequency *k-mers* and identifies repeat family sequences by retrieving sub-strings of the input sequences that contain specific *k-mers*. ReAS is an algorithm that uses unassembled reads from a whole-genome shotgun to recover ancestral sequences for TEs. For a *k-mer* seed, ReAS aligns all hits and uses those with sequence similarity to produce a 100 bp initial consensus sequence centered at the *k-mer*, and uses a greedy search algorithm to identify other high-frequency *k-mers* in the initial consensus sequence and extend the alignment. GRF is a genome-wide *de novo* repeat detection tool developed based on a combination of efficient and accurate numerical calculation algorithms and optimized dynamic programming strategies, which can sensitively identify terminal inverted repeats and terminal direct repeats, and interspersed repeats with reverse and direct repeats. Repeatmodeler2 is a user-friendly package that automatically discovers TE families in the genome, generates reference TE libraries, and produces high-quality libraries that recapitulate the known composition of three model species with some of the most complex TE landscapes. RepeatModeler2 significantly enhances the discovery and annotation of TEs in genome sequences.

The third class of methods, including RepARK [182], REPdenovo [183], RepAHR [184], and RepLong [185], rely on sequence assembly and community detection in sequence similarity network to detect repeats (**Supplementary Fig. S9**). Of these four methods, the first three are suitable for NGS short reads, among which RepARK and REPdenovo obtain repeats based on high-frequency *k-mers* assembly, while RepAHR obtains repeats by assembling high-frequency paired-end reads. The last method, RepLong, is one of the few identification methods that only rely on TGS long reads, which constructs a sequence similarity network based on the overlaps between the PacBio long reads, and uses the community discovery strategy to obtain repeats in the similarity network. RepARK obtains repeats by the assembly of high-frequency *k-mers*, which avoids potential biases by using abundant *k-mers* of the whole-genome short reads without requiring a reference genome. REPdenovo aims to construct repeats with relatively high copy numbers and low sequence divergence with copies of the repeats. RepAHR is proposed to solve the problem that assembly of short *k-mers* may destroy the structure of the repeats in genomes, which generates repeats by the assembly of high-coverage reads that contain a certain proportion of high-frequency *k-mers*. RepLong fills a gap in the field of repeat detection based on TGS, which can handle data with low coverage, and the modularity optimization method is employed in it to perform community discovery [186–188].

The NGS reads, or *k-mers*, are too short of identifying the full-length repeats, and the TGS long reads are with a high rate of sequencing errors, making the *de novo* methods typically fail to achieve satisfactory performance in terms of accuracy and completeness. The typical *de novo* methods are introduced in **Supplementary Table S8**. Different detection methods have different advantages and disadvantages. The typical homology-based, structure-based, and *de novo* detection methods, their principle description, benefits, and weaknesses are introduced in **Supplementary Tables S9-S11**.

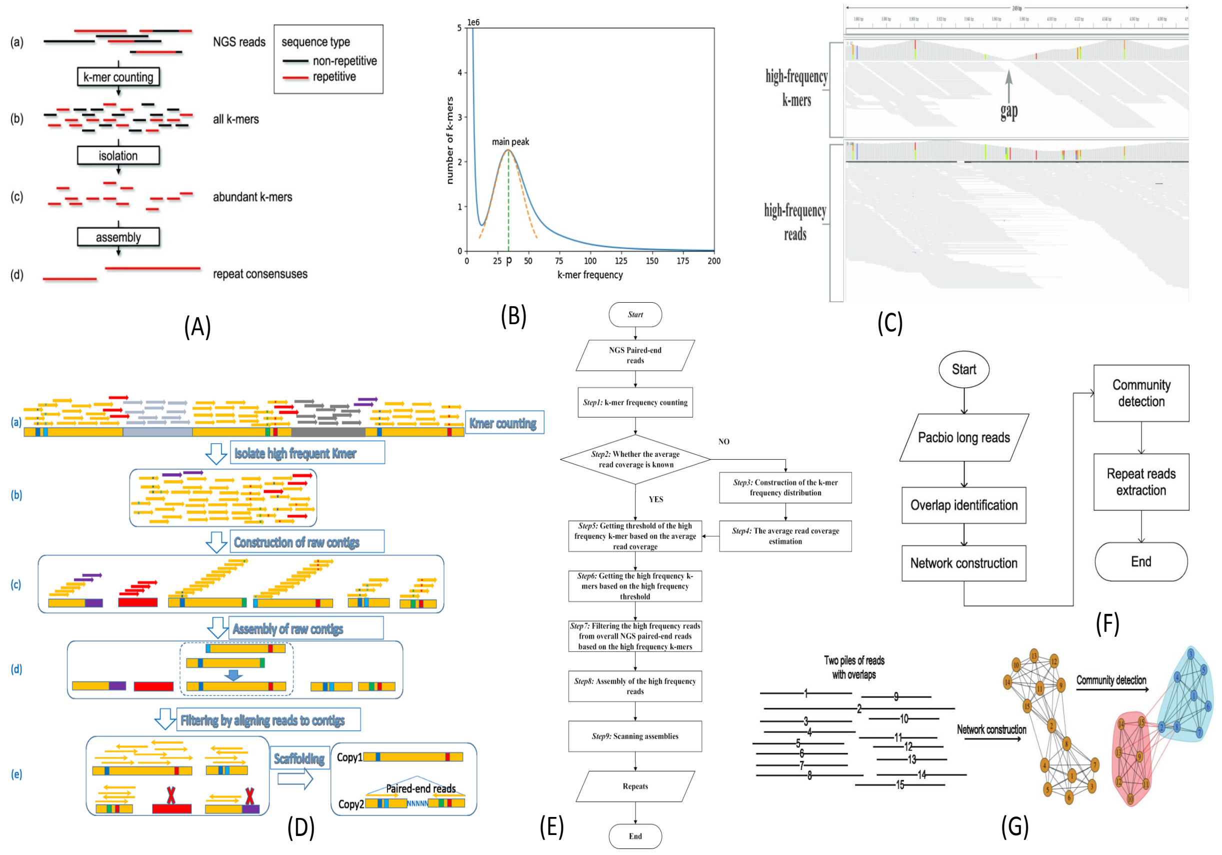

Supplementary Figure S9. The workflow of detection methods based on sequence assembly and community detection in sequence similarity network. Sub-graph(A): The workflow of RepARK. Sub-graph(B): The *k-mer* abundance histogram. Sub-graph(C): The advantage of assembling high-frequency reads compared to the assembly of high-frequency *k-mers*. Sub-graph(D): The workflow of REPdenovo. Sub-graph(E): The workflow of RepAHR. Sub-graph(F): The workflow of RepLong. Sub-graph(G): The principle of discovery community in sequence similarity network.

Supplementary Table S6. Introduction of typical homology-based detection methods.

Method type	Method name	Description	Advantages/Disadvantages
Homology-based	Censor	Censor (https://www.girinst.org/downloads/software/censor/) consists of an unaltered version of RepBase (it can also apply user-supplied libraries if desired), Perl and C++ modules. Censor identifies interspersed and tandem repeats based on sequence similarity comparisons. It analyzes repetitive sequences using repeat elements and their annotation information provided by RepBase Update. There are three main steps in Censor's pipeline.<br>▶ The first step is data pre-processing. In this step, long sequences are cut into smaller pieces to reduce the memory requirements of the aligner BLAST [150] and facilitate job splitting and scheduling tasks on multi-processor machines.<br>▶ The second step is similarity searching. In this step, BLAST is used as an aligner to compare the input sequence to annotated elements recorded in RepBase or a custom user-supplied library.<br>▶ The third step is post-processing and output. In this step, the program removes overlapping BLAST output, performs shard integration based on the detected repeats, and generates a report file with the '.map' suffix recording the repetitive elements and their locations.	**Advantages**:<br>▶ It can be used to classify all known repeats and generate reports automatically.<br>▶ High detection accuracy.<br>▶ It provides online identification services (https://www.girinst.org/censor/help.html).<br>**Disadvantages**:<br>▶ Highly reliant on homologous databases (RepBase, Dfam, etc.), and cannot discover novel repeats that have not been collected in homology databases.<br>▶ Using BLAST as the alignment algorithm often results in a long run time.<br>▶ The integrity of detection results often depends on the integrity of the homology databases.
	Greedier	Greedier is another homology-based detection algorithm for finding fragmented and nested repeats in a target genome based on a given repeat library. Greedier is implemented based on the idea of multiple iterations. Each iteration can be divided into the following two stages.<br>▶ In the first stage, Greedier determines the subsequence pairs that meet the requirements by the local alignment between the repeat library and target genome and constructs a graph according to the detected subsequence pairs, where each vertex represents a pair of subsequences similar to one another. Each edge denotes pairs of subsequences that can be connected to establish higher similarities.<br>▶ In the second stage, Greedier uses a greedy algorithm to traverse the graphs constructed in the first stage to determine matches to individual repeat units in the repeat library. For each match, it calculates a fitness value that indicates the matching similarity. After removing matches with fitness values over a threshold, the remaining genome is pieced together.	**Advantages**:<br>▶ Fewer false positives in detection results (From the experimental results of the paper).<br>▶ It can be used to report potential nested transposon structures (From the introduction of the method in the paper).<br>**Disadvantages**:<br>▶ Greedier is limited by the accuracy and completeness of the repeat library.<br>▶ The corresponding code of the method could not be found. The contribution of the method is primarily reflected in theory.
	RepeatMasker	RepeatMasker (https://www.repeatmasker.org/) is a program that screens DNA sequences for interspersed repeats and low-complexity DNA sequences. The new addition to the RepeatMasker package is a program that can also be used to identify the repetitive elements within protein sequences. Currently, over 56% of the human genomic sequence is identified and masked by the tool. The principle of RepeatMasker is to search for the occurrence of any reference sequence contained in a library (currently Dfam and RepBase, or a user-builtin library) in a query sequence using a sequence comparison approach based on popular search engines including nhmmer, cross_match, AB-BLAST/WU-BLAST, RM-BLAST, and Decypher. RepeatMasker provides users with viable options to meet the needs appropriate for various cases. The execution of RepeatMasker can be split into seven steps:<br>▶ Verify the hit point with a valid alignment tool (e.g., nhmmer, cross_match, AB-BLAST/WU-BLAST, and RM-BLAST).<br>▶ Read and check the input sequences.<br>▶ Check the RepeatMasker library (e.g., RepBase/Dfam) or the user TEs library.<br>▶ Split the sequences into fragments and prepare a list of executions on the fragments.<br>▶ Launch the alignment tool on the sequences.<br>▶ Change the search engine output to the RepeatMasker standard output.<br>▶ Merge the fragment sequences and merge the fragmented hits of TEs.	**Advantages**:<br>▶ Fewer false positives and highly accurate detection results.<br>▶ There is no restriction on the number of input sequences or the length of the sequences for RepeatMasker.<br>▶ RepeatMasker can also be used to identify the repetitive elements within protein sequences.<br>▶ RepeatMasker can also be accessed through the web (https://www.repeatmasker.org/cgi-bin/WEBRepeatMasker).<br>**Disadvantages**:<br>▶ Highly reliant on homologous databases (RepBase, Dfam, etc.), and cannot discover novel repeats that have not been collected in homology databases.<br>▶ The BLAST algorithm is the foundation of the four alignment tools (nhmmer, cross_match, AB-BLAST/WU-BLAST, and RM-BLAST), often resulting in a long run time.<br>▶ The integrity of detection results often depends on the integrity of the homology databases.

Supplementary Table S7. Typical structure-based detection methods.

Method type	Method name	Description	Advantages/Disadvantages
Structure-based	LTRharvest	The LTRharvest method (https://www.girinst.org/downloads/software/censor/) is a *de novo* detection algorithm used to detect full-length *LTR* elements in large sequence sets based on known features, such as length, distance, and sequence motifs of *LTR* transposons. The workflow of LTRharvest is summarized as follows:<br>▶ Constructing an improved suffix array for genomic chromosomes under consideration.<br>▶ Loading an enhanced suffix array into the main memory and conducting a subsequent search for the most extensive exact repeat based on this data structure.<br>▶ Testing candidate pairs against *LTR* retrotransposon-specific features (i.e., TSD and palindromic *LTR* motifs). The testing process is to search for TSDs with user-specified minimum and maximum lengths to the left and right of a candidate pair's $5'$ and $3'$ instance. The palindromic *LTR* motif consists of two pairs of two nucleotides and an allowed number of mismatches between these.<br>▶ Determining whether the user-specified *LTR* distance and length constraints are met for each remaining candidate pair. Additionally, *LTR* sequences containing TSDs and motifs (corresponding to the candidate pairs) are checked for a user-defined minimum sequence identity.	**Advantages**:<br>▶ Allows users to make flexible parameter settings.<br>▶ The algorithm has the characteristics of high efficiency, low memory, and disk-space consumption so that it can handle large species, such as vertebrates.<br>▶ The algorithm is powerful for *de novo* annotating high-quality, full-length, or nearly-full-length LTR retrotransposons.<br>**Disadvantages**:<br>▶ The LTRharvest method cannot detect partial short LTR retrotransposon copies, solo LTRs, and some nested elements.<br>▶ The LTRharvest method cannot check the presence of *LTR* retrotransposon-specific open reading frameworks (ORFs), primer binding sites, or polypurine tracts.
	SINE_scan	The SINE_scan method (https://github.com/maohlzj/SINE_scan) is a highly efficient structure-based algorithm for predicting *SINEs* in genomic DNA sequences by combining the hallmarks of *SINE* transposition, copy number, and structural signals. The SINE_scan program comprises the following three core modules.<br>▶ A collection of the *SINE* candidates by *de novo* identification method. An enhanced version of SINE-Finder is used in the SINE_scan program as the default detection tool for *SINE* candidate collection, which can identify all three types (*tRNA*, *7SLRNA*, and *5SRNA*) of *SINEs*.<br>▶ The validation of the *SINE* candidates using a copy number and transposition hallmark. Only candidates with a copy-number of full-length elements higher than a certain threshold (controlled by the parameter '-n'; default=5) are kept.<br>▶ Classification and genome-wide annotation. This module first classifies all verified *SINEs* into families according to the 80% identity rule using the CD-HIT suite, then compares them to known *SINEs* deposited publicly available repeat databases, such as the RepBase, SINEBase, and PGSB repeat databases.	**Advantages**:<br>▶ The SINE_scan method is designed to be flexible and robust for diverse purposes of *SINE* annotation and verification.<br>▶ The SINE_scan method can more comprehensively detect *SINEs* in genomes and discover numerous new *SINEs*.<br>**Disadvantages**:<br>▶ Highly reliant on structure databases, such as RepBase, SINEBase and PGSB repeat databases, and it is difficult to discover novel repeats whose structural features have not been collected in structure databases.<br>▶ The integrity of detection results often depends on the integrity of the database structure.
	MITE-Hunter	The MITE-Hunter method primarily comprises Perl scripts and a Unix program pipeline for discovering MITEs from genomes and produces outputs of consensus sequences classified into families. The workflow of MITE-Hunter comprises the following five main steps.<br>▶ Use a structure-based approach to identify TE candidates. Terminal inverted-repeat (TIR)-like structures (default 10 bp with at most 1 bp mismatch) flanked by putative TSDs (2 to 10 bp; default is TA if TSD length = 2) are used to identify TE candidates from each fragment sequence.<br>▶ Identify and filter false positives using a pair-wise sequence alignment-based approach. For candidate TEs and their flanking sequences, an all-by-all blastn comparison (default E-value = 1e-10) is performed. Single-copy candidates are identified and filtered from the blastn results.<br>▶ Generate exemplars. First, MITE-Hunter cluster candidate TEs based on the similarity between the TE sequences and selects the most representative sequence in each cluster as the category representative.<br>▶ Using an MSA approach, identify and filter false positives, generate consensus sequences, and predict TSDs.<br>▶ Group consensus sequences into the corresponding families.	**Advantages**:<br>▶ The fundamental drawback of the current *MITE* discovery programs, a significant false-positive rate, is effectively addressed by MITE-Hunter.<br>▶ Compared with existing programs, MITE-Hunter can more completely discover Class II non-autonomous TEs, particularly *MITEs*.<br>**Disadvantage**:<br>▶ The MITE-Hunter approach is implemented based on the pair-wise sequences alignment and false-positive filtering modules and is faster and more sensitive than MSA-based algorithms. Although MITE-Hunter has false-positive filtering modules, the false-positive rate of its results is still significantly higher than that of the methods based on MSA.

Supplementary Table S8. Introduction of typical *de novo* methods.

Method type	Method name	Description	Advantages/disadvantages
de novo	RepeatScout	The RepeatScout method (http://bix.ucsd.edu/repeatscout/) is a *de novo* identification algorithm that finds repeat families by extending consensus seeds, allowing for a precise determination of repeat boundaries.<br>▶ Builds a table of high-frequency *l-mers*.<br>▶ Extends the most frequent *l-mer* to a repeat family consensus sequence Q.<br>▶ Identifies occurrences of Q in the genome and adjusts *l-mer* frequency counts to exclude counts from occurrences of Q and proceeds to the most frequently remaining *l-mer*.<br>▶ The algorithm terminates when no *l-mers* with a frequency at least m, a fixed *l-mer* frequency threshold.	**Advantages**:<br>▶ The algorithm runs efficiently.<br>▶ The detection results of the algorithm are pure and accurate.<br>**Disadvantages**:<br>▶ The integrity of the detection results of the algorithm is usually unsatisfactory.<br>▶ The algorithm cannot process more than 1 Gb of the genome at a time.<br>▶ The size change of *l-mer* has a greater effect on the detection results.
	RepARK	RepARK (https://github.com/PhKoch/RepARK) is a *de novo* repetitive motif detection algorithm based on the assembly of high-frequency *k-mers*, which are orders of magnitude faster than the other methods and generate libraries that are (i) composed almost entirely of repetitive motifs, (ii) more comprehensive, and (iii) almost completely annotated by the TEclass tool [189]. The workflow of the RepARK program is summarized as follows:<br>▶ Converting the NGS short reads into *k-mers* of a certain length.<br>▶ Counting the frequency of *k-mers*.<br>▶ Separating high-frequency *k-mers* according to the high-frequency threshold.<br>▶ Assembling the high-frequency *k-mers* using a *de novo* genome assembly program, such as Velvet, into repeat consensus sequences.	**Advantages**:<br>▶ The algorithm runs efficiently.<br>▶ The algorithm consumes less computing resources (CPU, memory and disk space).<br>**Disadvantages**:<br>▶ The accuracy of detection results of the algorithm is general and the integrity is poor.<br>▶ The threshold of the high-frequency *k-mer* is challenging to determine, dramatically affecting the integrity and accuracy of the final detected repeats.
	RepLong	The RepLong method (https://github.com/ruiguo-bio/replong) is a *de novo* repeat identification method suitable for TGS long reads. The pipeline of RepLong consists of the following three stages : (i) identification of the overlaps between long reads, (ii) construction of a similarity network based on overlaps, and (iii) extraction of repeats from the network based on community detection. The workflow of RepLong is summarized in detail as follows:<br>▶ The pair-wise alignment of the reads is used to construct a read overlap similarity network. In this network, each vertex represents a read, and an edge represents the substantial overlap between the two corresponding reads.<br>▶ Network modularity optimization is used to locate communities with stronger internal than external connectivity.<br>▶ Representative reads from each community are collected to construct the repeat library.	**Advantages**:<br>▶ The RepLong approach can directly obtain repeats and only relies on TGS long reads.<br>▶ Compared with existing *de novo* detection methods (e.g., RepARK and REPdenovo), RepLong tends to obtain repeats more completely.<br>**Disadvantages**:<br>▶ This algorithm usually consumes vast computing resources (CPU, memory, and disk space) and has a long run time.<br>▶ The detection accuracy of the algorithm is usually unsatisfactory.
	LongRep Marker	The LongRepMarker method (https://github.com/Xingyu-Liao/LongRepMarker_v2.0) is a hybrid framework for sensitively detecting repeats based on short and long reads. It is designed based on strategies of a hybrid global *de novo* assembly of long and short reads and overlap detection based on multi-alignment unique *k-mers*, which can be used for precise identification and classification of comprehensive repeats in the genome. The LongRepMarker'workflow consists of the following steps:<br>▶ Identifying overlap sequences between chromosomes/contigs/long reads.<br>▶ Converting overlap sequences into unique *k-mers*.<br>▶ Generating coverage regions on overlap sequences that can be covered by multi-alignment unique *k-mers*.<br>▶ Classifying coverage regions on overlap sequences that can be aligned using multi-alignment unique *k-mers*.<br>▶ Calling genetic variants that exist in repetitive regions.<br>▶ Generating the TE consensus sequences by combining fragments with relationships of duplication or inclusion.<br>▶ Classifying TE consensus sequences and generating the final identification results with several detection reports.	**Advantages**:<br>▶ By assembling the overall NGS short and long sequencing fragments (barcode linked reads and TGS long reads) rather than the high-frequency *k-mers*, it can largely recover the repeats in the genome.<br>▶ By detecting the overlap sequences between assemblies/chromosomes/long reads, it can more quickly and accurately locate repetitive regions.<br>▶ Using the multi-alignment unique *k-mer*-based overlap detection strategy, it can more comprehensively and stably identify repeats.<br>▶ This algorithm can also be used to detect TRs.<br>**Disadvantage**:<br>▶ This algorithm may consumes substantial memory space and have a relatively long run time when dealing with large genomes.

Supplementary Note 7.2 Performance comparison between different detection methods

Supplementary Note 7.2.1 Datasets We evaluated the performance of Greedier, RepeatMasker, Corss_match, WindowMasker, LTR-STRUC, LTR-Seq, LTR-Rho, LTR-FINDER, LTRharvest, MITE-Hunter, FINDMITE, MUST, AnnoSINE, SINE-Finder, SINE_Scan, SINE_Base, RepeatModeler, RepMasker, RepeatScout, RepeatModeler2, RepARK, REPdenovo, RepLong, and LongRepMarker based on 20 datasets. The details of these datasets are shown in **Supplementary Tables S9-S11**.

Supplementary Table S9. Details of the experimental data

Test items	Species	Dataset Name	Datasize (KB)	Source
Reference	Leafcutter Ant	GCA_000204515.1_Aech_3.9 _genomic_Ant.fna	293,052	https://www.ncbi.nlm.nih.gov/
	D.melanogaster	dmel-all-chromosome- r5.43.fasta	168,080	https://www.ncbi.nlm.nih.gov/
	Soybean	Glycine_max_Soybean.fna	968,211	https://www.ncbi.nlm.nih.gov/
	Gallus	Gallus_gallus.fna	1,053,454	https://www.ncbi.nlm.nih.gov/
	Mouse	GCA_000001635.8_GRCm38.p6 _genomic_Mouse.fna	2,787,341	https://www.ncbi.nlm.nih.gov/
	Human(hg38)	GCF_000001405.39_genomic _Human.fna	3,196,759	https://www.ncbi.nlm.nih.gov/
	Arabidopsis	GCF_000004255.2_v.1.0_genomic.fna	204,585	https://www.ncbi.nlm.nih.gov
	Rice	GCF_002938485.1_Soryzae_2.0 _genomic.fna	762,197	https://www.ncbi.nlm.nih.gov/
	Maize	GCF_902167145.1_Zm-B73-REFERENCE-NAM-5.0_genomic.fna	2,158,363	https://www.ncbi.nlm.nih.gov/
	S.cerevisiae	GCF_000002945.1_ASM294v2.fna	168,080	https://www.ncbi.nlm.nih.gov/
Annotation	Arabidopsis	gene models	5,446	https://www.arabidopsis.org/
	Rice	gene models	61	https://www.arabidopsis.org/
NGS short reads	Leafcutter Ant	ERR034186_1.fastq	17,580,863	https://www.ncbi.nlm.nih.gov/
		ERR034186_2.fastq	17,580,863	https://www.ncbi.nlm.nih.gov/
	D.melanogaster	SRR350908_1.fastq	5,767,698	https://www.ncbi.nlm.nih.gov/
		SRR350908_2.fastq	5,767,698	https://www.ncbi.nlm.nih.gov/
	Mouse	ERR2894257_1.fastq	26,655,537	https://www.ncbi.nlm.nih.gov/
		ERR2894257_2.fastq	26,655,537	https://www.ncbi.nlm.nih.gov/
	Human-chr14	frag_1.fastq	4,913,897	http://gage.cbcb.umd.edu/
		frag_2.fastq	4,913,897	http://gage.cbcb.umd.edu/
	HG003_24149_father	D2_S2_L001_R1_001.fastq	23,534,426	ftp://ftp-trace.ncbi.nlm.nih.gov/giab/ftp/data
		D2_S2_L001_R2_001.fastq	23,534,426	ftp://ftp-trace.ncbi.nlm.nih.gov/giab/ftp/data
SMS long reads	D.melanogaster_100k	dro_100k.fa	919,162	https://github.com/ruiguo-bio/replong
	Homo sapiens_100K	human_100k.fa	507,871	https://github.com/ruiguo-bio/replong
	D.melanogaster_900k	dmel_filtered.fastq	30,885,716	https://github.com/ruiguo-bio/replong
	Homo sapiens_900k	human_polished.fastq	109,716,724	https://github.com/ruiguo-bio/replong

Supplementary Note 7.2.2 Evaluation metrics In order to comprehensively evaluate the performance of the compared methods, we used 19 evaluation metrics in this experiment, which are Num, Max(kb), N50(kb), N75(kb), N90(kb), 0 time, 1 times, >1 times, Mapping Rate(%), Reference(%), Repbase(%), Time (hour) and Memory(MB) (**Supplementary Table S12**). 'Num' denotes the number of segments; 'Max(kb)'

Supplementary Table S10. Detail of test genomes

Datasets	Genomes					
	H.sapiens (hg38)	Mouse	L.Ant	Gallus	D.melanogaster	Glycine max
Species type	Eukaryote	Eukaryote	Eukaryote	Eukaryote	Eukaryote	Eukaryote
Genome size(bp)	3,209,286,105	2,818,974,548	295,944,863	1,065,365,434	168,736,537	979,046,046
Number of chromosomes	455	239	4339	464	15	1192
Longest chromosome(bp)	248,956,422	195,471,971	5,247,136	197,608,386	29,004,656	58,018,742
Shortest chromosome(bp)	970	1,976	200	87	19,517	1002

'H.sapiens' represents the dataset of Homo sapiens; 'D.melanogaster' represents the dataset of Drosophila melanogaster; 'L.Ant' represents the dataset of Leafcutter Ant; 'G.gallus' represents the dataset of Gallus gallus(chicken); 'Glycine max' represents the dataset of Glycine max(Soybean).

Supplementary Table S11. Details of NGS short reads

Datasets	NGS short reads					
	Saccharomyces	Human14	Human_wgs	Drosophila melanogaster	Acromyrmex	Mouse
Species type	Eukaryote	Eukaryote	Eukaryote	Eukaryote	Eukaryote	Eukaryote
Genome size(Mbp)	12.157	106.332	3,209.286	168.080	295.944	2,818.974
Sequencing technology	Illumina	Illumina	Illumina	Illumina	Illumina	Illumina
Library type	Single-lib	Single-lib	Single-lib	Single-lib	Single-lib	Single-lib
Read length(bp)	301	101	108	100	100	100
Number of reads	5,504,000	145,778,752	60,007,256	39,468,243	106,748,982	106,748,982
Average Coverage	∼136.27	∼138.46	∼4.02	∼23.48	∼36.42	∼36.42
Insertsizes(bp)	400	155	388	358	500	500

'Human14' indicates the sequencing data of chromosome 14 in the human genome, 'Human_wgs' represents the whole genome sequencing data of the human genome.

denotes the length of the largest segment; ′N50(kb)′ is the length of the longest segment such that all the segments longer than this segment cover at least half (50%) of the total length of all segments; ′N75′ and ′N90′ are calculated in a similar way; ′0 time′ indicates the proportion of segments that cannot be aligned to the reference sequence in all segments; ′1 time′ indicates the proportion of segments that can be aligned to a unique location on the reference sequence in all segments; ′ >1 times' indicates the proportion of segments that can be aligned to multiple locations on the reference sequence in all segments; ′Mapping Rate(%)′ indicates the proportion of segments that can be aligned to the reference sequence in all segments; ′Reference(%)′ indicates the proportion of regions marked as repetitive regions in the reference sequence that can be covered with the segments; ′Repbase(%)′ indicates the proportion of fragments in Repbase that can be covered with segments; ′Annotations′ indicates the total number of annotation transposable elements in the dataset; ′Predictions′ indicates the number of transposable elements predicted by method; ′Sensitivity′ indicates the ability to predict the true positives of each available category; ′Specificity′ indicates the ability to predict the true negatives of each available category; ′PDR′ indicates the false discovery rate; ′F1-score′ indicates the precision and recall of a classifier into a single metric by taking their harmonic mean; ′Time (hour)′ indicates the time consumption of algorithms; ′Memory(MB)′ indicates the peak memory consumption of algorithms.

Supplementary Table S12. Evaluation metrics

Metrics	Meaning
Num	The number of segment
Max(kb)	The length of the largest segment
N50(kb)	The length of the longest segment such that all the segments longer than this segment cover at least 50% of the total length of all segments
N75(kb)	The length of the longest segment such that all the segments longer than this segment cover at least 75% of the total length of all segments
N90(kb)	The length of the longest segment such that all the segments longer than this segment cover at least 90% of the total length of all segments
0 time	The proportion of segments that cannot be aligned to the reference sequence in all segments
1 time	The proportion of segments that can be aligned to a unique location on the reference sequence in all segments
> 1 times	The proportion of segments that can be aligned to multiple locations on the reference sequence in all segments
Mapping Rate(%)	The proportion of segments that can be aligned to the reference sequence in all segments
Repbase(%)	The proportion of fragments in Repbase that can be covered with segments
Reference(%)	The proportion of regions marked as repetitive regions in the reference sequence that can be covered with the segments
Annotations	The total number of annotation transposable elements in the dataset
Accuracy	The ratio of correctly predicted observation to the total observations.
Predictions	The number of transposable elements predicted by method
Sensitivity	The metric that evaluates the ability of a method to predict the true positives of each available category
Specificity	The metric that evaluates the ability of a method to predict the true negatives of each available category
PDR	The false discovery rate is the ratio of the number of false positive results to the number of total positive test results. FDR = expected (# false predictions / # total predictions)
Recall	The measure of how many of the positive cases the classifier correctly predicted, over all the positive cases in the data
F1-score	The F1-score combines the precision and recall of a classifier into a single metric by taking their harmonic mean
Time (hour)	The run time consumption of algorithms
Memory(MB)	The peak memory consumption of algorithms

Supplementary Note 7.2.3 Performance comparison The comparison of the performance of homology-based methods, including Greedier, RepeatMasker, corss_match, and WindowMasker, in terms of bases masked in the genomes of Arabidopsis and Rice, is shown in **Supplementary Table S13**. The quality validation of structural-based methods LTR_STRUC, LTR_Seq, LTR_Rho, LTR_FINDER, and LTRharvest on the genomes of S.cerevisiae and D.melanogaster is shown in **Supplementary Table S14**. The performance comparison of structural-based methods MITE-Hunter, FINDMITE, detectMITE, GRF-mite_dft, MITE-Tracker, TIR-Learner, and MUST on the Rice genome is shown in **Supplementary Tables S15 to S16**. The comparison of the element-level performance of different structural-based SINE annotation methods AnnoSINE, SINE_FINDER, SINE_Scan, SINE_Base, RepeatModeler is shown in **Supplementary Table S17**. The comparison of the proportion and detailed classification of elements in the reference genomes and the corresponding RepBase libraries is covered by the detection results of *de novo* detection methods RepeatScout, RepeatModeler2, RepARK, REPdenovo, RepLong, LongRepMarker, and standard benchmark method RepeatMasker is shown in **Supplementary Tables S18-S32**.

Supplementary Table S13. Comparison of Greedier, RepeatMasker, cross_match, and WindowMasker in terms of bases masked in different regions of the Arabidopsis and Rice genomes.

		Number of bases masked				Percentage of bases masker			
Region	#bases annotated	Greedier	RepeatMasker	cross_match	WM	Greedier	RepeatMasker	cross_match	WM
Arabidopsis whole-genome	119,186,497	3,831,443	8,506,912	3,725,050	22,177,358	3.2	7.1	3.1	18.5
(TP)	5,905,785	924,076	2,087,175	385,889	344,000	16	35.68	6.8	5.8
(FP)	42,900,000	324,635	860,241	1,028,240	3,230,000	0.78	2	2.4	7.5
# 10 chromosome of the rice genome	22,876,596	3,973,477	6,839,111	4,594,861	4,315,506	17.4	29.9	20	18.9
(TP)	3,072,087	1,481,468	2,051,697	641,277	461,174	48.2	66.8	20.9	15
(FP)	3,297,203	101,616	181,082	535,830	293,923	3.1	5.5	16.3	8.9

'WM' represents the method WindowMasker, transposons (TP), and other exons (FP). The TP/FP rates of Greedier, RepeatMasker, cross_match, and WindowMasker are 2.85, 2.42, 0.37, and 0.10, respectively.

Supplementary Table S14. Quality validation of programs for LTR retrotransposon prediction on the genomes of S.cerevisiae and D.melanogaster.

Species	Program used	LTR_STRUC	LTR_Seq	LTR_Rho	LTR_FINDER	LTRharvest
S. cerevisiae	Run-time [s]	∼600	413	190	19	3
	Annotations	50	50	47	50	50
	Predictions	39	50	46	56	68
	Sensitivity	76%	80%	89.4%	100.0%	98.0%
	Specificity	97.4%	100.0%	91.3%	89.3%	72.1%
D. melanogaster	Run-time [s]	4380	24120	2286	1209	25
	Annotations	304	304	304	304	340
	Predictions	310	188	417	395	723
	Sensitivity	37.5%	36.8%	94.7%	74.3%	94.7%
	Specificity	36.8%	59.6%	69.1%	57.2%	40.4%

Supplementary Table S15. Comparison of MITE-Hunter with FINDMITE and MUST on the chromosome #12 of the Rice genome.

Program	Running time	Predicted TEs	False-positive(%)
MITE-Hunter	1.7h	114	4.4
FINDMITE	<1h	10,864	85.0
MUST	5.5h	5,485	86.0

Supplementary Table S16. Comparison of the new TIR candidates discovered by several MITE and TIR identification tools based on Rice genome.

	TIRs and MITEs				
Tools	New TE	New TE with known TIRs	Unique new TE with new TIRs	New TIRs with conserved domains	New TIRs with copy number > 3
detectMITE	15,654	10,947	1,341	159	1,018
GRF-mite_dft	1,489	687	354	159	311
MITE-Hunter	114	144	0	0	0
MITE-Tracker	836	137	668	126	577
TIR-Learner	13,317	4,104	6,461	252	2,893

Supplementary Table S17. Element-level performance of different SINE annotation tools on the Arabidopsis and Rice genomes.

Species	Metrics	AnnoSINE	SINE-Finder	SINE_Scan	SINE_Base	RepeatModeler
arabidopsis	F1-score	0.928	0.081	0.255	0.851	0.772
	Sensitivity	0.955	0.901	0.146	0.772	0.734
	PDR	0.097	0.958	0.024	0.052	0.186
	Precision	0.903	0.042	0.976	0.948	0.841
rice	F1-score	0.924	0.072	0.569	0.705	0.457
	Sensitivity	0.890	0.803	0.492	0.545	0.305
	PDR	0.040	0.963	0.327	0.002	0.092
	Precision	0.960	0.037	0.673	0.998	0.908

Supplementary Table S18. Comparison of LongRepMarker, RepeatScout, and RepeatMasker.

		Quast (length ≥ 5000bp)					Minimap2				RepeatMasker	
Species	Tool	Time(min)/Peak Mem(GB)	Max (kb)	N50 (kb)	N75 (kb)	N90 (kb)	0 time	1 time	>1 time	Mapping Rate (%)	Reference (%)	Repbase (%)
H.sapiens(hg38)	LongRepMarker	2863.539/46.688	1034.338	83.195	28.812	10.281	0.00%	11.75%	88.25%	100.0%	37.20%	81.61%
	RepeatScout	Error	Error	Error	Error	Error	Error	Error	Error	Error	Error	Error
	RepeatMasker	12696.500/71.808	1499.996	7.228	6.133	5.616	0.00%	92.63%	7.37%	100.0%	NA	80.01%
Mouse	LongRepMarker	2979.584/42.868	339.188	16.526	7.112	6.061	0.00%	24.49%	75.51%	100.0%	40.27%	68.36%
	RepeatScout	Error	Error	Error	Error	Error	Error	Error	Error	Error	Error	Error
	RepeatMasker	11734.183/65.234	78.144	6.409	6.092	5.391	0.01%	82.61%	17.39%	99.99%	NA	68.18%
Leafcutter Ant	LongRepMarker	9.954/18.800	17.329	12.961	12.961	9.639	0.00%	0.00%	100.0%	100.0%	4.48%	12.89%
	RepeatScout	49.866/6.068	5.740	5.695	5.058	5.058	0.00%	0.00%	100.0%	100.0%	3.63%	11.85%
Gallus	LongRepMarker	73.538/32.167	24.886	8.040	6.434	5.583	0.00%	0.26%	99.74%	100.0%	12.50%	NA
	RepeatScout	Error	Error	Error	Error	Error	Error	Error	Error	Error	Error	NA
D.melanogaster	LongRepMarker	36.166/24.021	41.224	9.225	9.115	9.092	0.00%	0.20%	99.80%	100.0%	13.83%	23.80%
	RepeatScout	31.933/3.086	20.015	9.511	6.423	5.377	0.00%	0.00%	100.0%	100.0%	10.55%	14.40%
Glycine max	LongRepMarker	248.059/36.567	34.756	20.023	17.264	15.974	0.00%	0.46%	99.54%	100.0%	34.16%	NA
	RepeatScout	214.183/22.990	16.383	8.267	6.313	5.310	0.00%	0.00%	100.0%	100.0%	33.40%	NA

The left sub-table shows the size statistics of detection results of each tool on various datasets, and the main evaluation indicators are Max(The longest contig), N50, N75, and N90. The middle sub-table shows the alignment ratio statistics of the detection results of LongRepMarker on various datasets, and the main evaluation indicators are '0 time (The proportion of fragments in detection results that can not be aligned to the reference genome)', '1 time (The proportion of fragments in detection results that can be aligned to the reference genome only one location)', '>1 times (The proportion of fragments in detection results that can be aligned to the reference genome many locations)' and 'Mapping rate(%) (The overall proportion of fragments in detection results that can be aligned to the reference genome)'. The right sub-table shows the proportion of repetitive fragments in the reference genome or repbase library that can be covered by the detection results. 'Time(min)/Peak Mem(GB)' represents the run time and peak memory consumption.

Supplementary Table S19. The proportion and detailed classification of elements in the RepBase library of Human is covered by the detection results of LongRepMarker, RepeatScout, and RepeatModeler2.

	LongRepMarker			RepeatScout			RepeatModeler2		
	**sequence: 1512<br>total length: 1647075bp<br>GC level: 45.30%<br>bases masked: 1213841 bp (82.45%))**			**sequence: 1512<br>total length: 1647075bp<br>GC level: 45.30%<br>bases masked: 1213841 bp (73.70%))**			**sequence: 1512<br>total length: 1647075bp<br>GC level: 45.30%<br>bases masked: 1213841 bp (63.33%))**		
**Repeat Types**	**Number of elements**	**Length occupied**	**Percentage of sequence**	**Number of elements**	**Length occupied**	**Percentage of sequence**	**Number of elements**	**Length occupied**	**Percentage of sequence**
SINEs:	709	255186 bp	15.49%	690	189225 bp	11.49%	87	26863 bp	1.63%
–ALUs:	690	251356 bp	15.26%	676	188001 bp	11.41%	74	25030 bp	1.52%
–MIRs:	17	3552 bp	0.22%	9	613 bp	0.04%	10	1566 bp	0.10%
LINEs:	1376	690975 bp	41.95%	624	298720 bp	18.14%	275	254509 bp	15.45%
–LINE1:	1337	682454 bp	41.43%	608	295084 bp	17.92%	244	242822 bp	14.74%
–LINE2:	11	1455 bp	0.09%	9	2517 bp	0.15%	9	5981 bp	0.36%
–L3/CR1:	5	708 bp	0.04%	4	805 bp	0.05%	18	4208 bp	0.26%
LTR elements:	566	327086 bp	19.86%	903	571647 bp	34.71%	1011	612530 bp	37.19%
–ERVL	98	39268 bp	2.38%	152	87126 bp	5.29%	188	119764 bp	7.27%
–ERVL-MaLRs	32	8118 bp	0.49%	47	13970 bp	0.85%	47	19783 bp	1.20%
–ERV_classI	370	220447 bp	13.38%	634	388908 bp	23.61%	709	402655 bp	24.45%
–ERV_classII	54	57466 bp	3.49%	56	79131 bp	4.80%	65	69756 bp	4.24%
DNA elements:	110	25838 bp	1.57%	223	64465 bp	3.91%	344	106865 bp	6.49%
–hAT-Charlie:	41	8781 bp	0.53%	48	11006 bp	0.67%	102	28766 bp	1.75%
–TcMar-Tigger:	35	11048 bp	0.67%	84	34543 bp	2.10%	107	36801 bp	2.23%
Unclassified:	185	57213 bp	3.47%	141	54603 bp	3.32%	8	2266 bp	0.14%
Total interspersed repeats:		1356298 bp	82.35%		1178660 bp	71.56%		1003033 bp	60.90%
Small RNA:	14	1276 bp	0.08%	51	11176 bp	0.68%	6	742 bp	0.05%
Satellites:	24	10205 bp	0.62%	31	12727 bp	0.77%	14	3414 bp	0.21%
Simple repeats:	216	31821 bp	1.93%	255	34087 bp	2.07%	279	34727 bp	2.11%
Low complexity:	11	483 bp	0.03%	18	851 bp	0.05%	27	1228 bp	0.07%

'sequence' (the number of fragments contained in the Human RepBase library). 'Base Masked (%)' (the ratio of the bases in RepBase that can be covered by the detected fragments). 'GC(%)' (the GC content). 'Length occupied'. (the length of the bases of the corresponding repetitive family in RepBase that can be covered by the detected fragments).

Supplementary Table S20. The proportion and detailed classification of elements in the RepBase library of Mouse is covered by the detection results of LongRepMarker, RepeatScout, and RepeatModeler2.

	LongRepMarker			RepeatScout			RepeatModeler2		
	**sequence: 1561**<br>**total length: 1680566bp**<br>**GC level: 44.70%**<br>**bases masked: 1044496 bp (62.15%))**			**sequence: 1561**<br>**total length: 1680566bp**<br>**GC level: 44.70%**<br>**bases masked: 987478 bp (58.76%))**			**sequence: 1561**<br>**total length: 1680566bp**<br>**GC level: 44.70%**<br>**bases masked: 907532 bp (54.00%))**		
**Repeat Types**	**Number of elements**	**Length occupied**	**Percentage of sequence**	**Number of elements**	**Length occupied**	**Percentage of sequence**	**Number of elements**	**Length occupied**	**Percentage of sequence**
SINEs:	325	62219 bp	3.70%	292	60826 bp	3.62%	77	10662 bp	0.63%
–ALUs:	272	52570 bp	3.13%	218	50691 bp	3.02%	44	6817 bp	0.41%
–MIRs:	8	1464 bp	0.09%	5	439 bp	0.03%	10	1220 bp	0.07%
LINEs:	822	581349 bp	34.59%	477	417481 bp	24.84%	276	357395 bp	21.27%
–LINE1:	820	579118 bp	34.46%	472	416637 bp	24.79%	275	357213 bp	21.26%
–LINE2:	1	94 bp	0.01%	4	205 bp	0.01%	1	182 bp	0.01%
–L3/CR1:	0	0 bp	0.00%	0	0 bp	0.00%	0	0 bp	0.00%
LTR elements:	493	343322 bp	20.43%	659	458817 bp	27.30%	848	483400 bp	28.76%
–ERVL	85	56252 bp	3.35%	83	54386 bp	3.24%	76	61939 bp	3.69%
–ERVL-MaLRs	57	10909 bp	0.65%	71	16563 bp	0.99%	59	16407 bp	0.98%
–ERV_classI	78	65804 bp	3.92%	117	86999 bp	5.18%	135	82306 bp	4.90%
–ERV_classII	265	207985 bp	12.38%	383	291835 bp	17.37%	575	31922 bp	18.99%
DNA elements:	57	7136 bp	0.42%	33	4009 bp	0.24%	36	9446 bp	0.56%
–hAT-Charlie:	32	3880 bp	0.23%	25	3117 bp	0.19%	24	5297 bp	0.32%
–TcMar-Tigger:	9	1410 bp	0.08%	1	107 bp	0.01%	9	3608 bp	0.21%
Unclassified:	53	20086 bp	1.20%	33	9440 bp	0.56%	18	6587 bp	0.39%
Total interspersed repeats:		1014112 bp	60.34%		950573 bp	56.56%		867490 bp	51.62%
Small RNA:	29	3693 bp	0.22%	55	4815 bp	0.29%	2	323 bp	0.02%
Satellites:	8	4208 bp	0.25%	9	4033 bp	0.24%	4	544 bp	0.03%
Simple repeats:	314	36351 bp	2.16%	327	36959 bp	2.20%	333	37141 bp	2.21%
Low complexity:	35	1618 bp	0.10%	40	1873 bp	0.11%	45	2227 bp	0.13%

′sequence′ (the number of fragments contained in the Mouse RepBase library). ′Base Masked (%)′ (the ratio of the bases in RepBase that can be covered by the detected fragments). ′GC(%)′ (the GC content). ′Length occupied′. (the length of the bases of the corresponding repetitive family in RepBase that can be covered by the detected fragments).

Supplementary Table S21. The proportion and detailed classification of elements in the RepBase library of Drosophila is covered by the detection results of LongRepMarker, RepeatScout, and RepeatModeler2.

	LongRepMarker			RepeatScout			RepeatModeler2		
	**sequence: 2489**<br>**total length: 7220516bp**<br>**GC level: 42.77%**<br>**bases masked: 3746452 bp (51.89%))**			**sequence: 2489**<br>**total length: 7220516bp**<br>**GC level: 42.77%**<br>**bases masked: 3491131 bp (48.35%))**			**sequence: 2489**<br>**total length: 7220516bp**<br>**GC level: 42.77%**<br>**bases masked: 3336440 bp (46.21%))**		
**Repeat Types**	**Number of elements**	**Length occupied**	**Percentage of sequence**	**Number of elements**	**Length occupied**	**Percentage of sequence**	**Number of elements**	**Length occupied**	**Percentage of sequence**
SINEs:	1	73 bp	0.00%	1	74 bp	0.00%	0	0 bp	0.00%
–ALUs:	0	0 bp	0.00%	0	0 bp	0.00%	0	0 bp	0.00%
–MIRs:	0	0 bp	0.00%	0	0 bp	0.00%	0	0 bp	0.00%
LINEs:	1317	1043230 bp	14.45%	1187	949761 bp	13.15%	1152	955570 bp	13.23%
–LINE1:	0	0 bp	0.00%	0	0 bp	0.00%	0	0 bp	0.00%
–LINE2:	0	0 bp	0.00%	0	0 bp	0.00%	0	0 bp	0.00%
–L3/CR1:	202	143230 bp	1.98%	147	106999 bp	1.48%	108	104755 bp	1.45%
LTR elements:	2515	2355715 bp	32.63%	2631	2194498 bp	30.39%	2254	2065761 bp	28.61%
–ERVL	0	0 bp	0.00%	0	0 bp	0.00%	0	0 bp	0.00%
–ERVL-MaLRs	0	0 bp	0.00%	0	0 bp	0.00%	0	0 bp	0.00%
–ERV_classI	0	0 bp	0.00%	0	0 bp	0.00%	0	0 bp	0.00%
–ERV_classII	0	0 bp	0.00%	0	0 bp	0.00%	0	0 bp	0.00%
DNA elements:	421	193452 bp	2.68%	524	177269 bp	2.46%	409	170180 bp	2.36%
–hAT-Charlie:	0	0 bp	0.00%	0	0 bp	0.00%	0	0 bp	0.00%
–TcMar-Tigger:	0	0 bp	0.00%	0	0 bp	0.00%	0	0 bp	0.00%
Unclassified:	166	53666 bp	0.74%	179	59066 bp	0.82%	139	32370 bp	0.45%
Total interspersed repeats:		3646136 bp	50.50%		3380668 bp	46.82%		3223881 bp	44.65%
Small RNA:	29	13271 bp	0.18%	27	13792 bp	0.19%	15	6003 bp	0.08%
Satellites:	17	6719 bp	0.09%	15	6065 bp	0.08%	4	544 bp	0.03%
Simple repeats:	1108	74336 bp	1.03%	1172	76644 bp	1.06%	1224	80026 bp	1.11%
Low complexity:	291	15714 bp	0.22%	295	16084 bp	0.22%	318	17088 bp	0.24%

′sequence′ (the number of fragments contained in the Drosophila RepBase library). ′Base Masked (%)′ (the ratio of the bases in RepBase that can be covered by the detected fragments). ′GC(%)′ (the GC content). ′Length occupied′. (the length of the bases of the corresponding repetitive family in RepBase that can be covered by the detected fragments).

Supplementary Table S22. The proportion and detailed classification of elements in the RepBase library of Soybean is covered by the detection results of LongRepMarker, RepeatScout, and RepeatModeler2.

	LongRepMarker			RepeatScout			RepeatModeler2		
	**sequence: 758**<br>**total length: 1646292bp**<br>**GC level: 42.57%**<br>**bases masked: 1536173 bp (93.31%))**			**sequence: 758**<br>**total length: 1646292bp**<br>**GC level: 42.57%**<br>**bases masked: 1535709 bp (93.28%))**			**sequence: 758**<br>**total length: 1646292bp**<br>**GC level: 42.57%**<br>**bases masked: 1375693 bp (83.56%))**		
**Repeat Types**	**Number of elements**	**Length occupied**	**Percentage of sequence**	**Number of elements**	**Length occupied**	**Percentage of sequence**	**Number of elements**	**Length occupied**	**Percentage of sequence**
SINEs:	1	18 bp	0.00%	0	0 bp	0.00%	2	145 bp	0.01%
–ALUs:	0	0 bp	0.00%	0	0 bp	0.00%	0	0 bp	0.00%
–MIRs:	0	0 bp	0.00%	0	0 bp	0.00%	0	0 bp	0.00%
LINEs:	45	80754 bp	4.91%	52	85283 bp	5.18%	67	72838 bp	4.42%
–LINE1:	44	77578 bp	4.71%	50	81968 bp	4.98%	65	69502 bp	4.22%
–LINE2:	0	0 bp	0.00%	0	0 bp	0.00%	0	0 bp	0.00%
–L3/CR1:	0	0 bp	0.00%	0	0 bp	0.00%	0	0 bp	0.00%
LTR elements:	881	1238562 bp	75.23%	1030	1238480 bp	75.23%	815	1114450 bp	67.69%
–ERVL	0	0 bp	0.00%	0	0 bp	0.00%	0	0 bp	0.00%
–ERVL-MaLRs	0	0 bp	0.00%	0	0 bp	0.00%	0	0 bp	0.00%
–ERV_classI	0	0 bp	0.00%	0	0 bp	0.00%	0	0 bp	0.00%
–ERV_classII	0	0 bp	0.00%	0	0 bp	0.00%	0	0 bp	0.00%
DNA elements:	130	154184 bp	9.37%	145	146753 bp	8.91%	139	123780 bp	7.52%
–hAT-Charlie:	0	0 bp	0.00%	0	0 bp	0.00%	0	0 bp	0.00%
–TcMar-Tigger:	0	0 bp	0.00%	0	0 bp	0.00%	0	0 bp	0.00%
Unclassified:	28	25065 bp	1.52%	34	31451 bp	1.91%	20	23872 bp	1.45%
Total interspersed repeats:		1498583 bp	91.03%		1501967 bp	91.23%		1335085 bp	81.10%
Small RNA:	22	6625 bp	0.40%	26	6988 bp	0.42%	2	5216 bp	0.32%
Satellites:	0	0 bp	0.00%	0	0 bp	0.00%	3	301 bp	0.02%
Simple repeats:	200	31493 bp	1.91%	215	32310 bp	1.96%	255	33830 bp	2.05%
Low complexity:	9	1018 bp	0.06%	11	824 bp	0.05%	21	1344 bp	0.08%

′sequence′ (the number of fragments contained in the Soybean RepBase library). ′Base Masked (%)′ (the ratio of the bases in RepBase that can be covered by the detected fragments). ′GC(%)′ (the GC content). ′Length occupied′. (the length of the bases of the corresponding repetitive family in RepBase that can be covered by the detected fragments).

Supplementary Table S23. The proportion and detailed classification of elements in the RepBase library of Gallus is covered by the detection results of LongRepMarker, RepeatScout, and RepeatModeler2.

	LongRepMarker			RepeatScout			RepeatModeler2		
	**sequence: 512**<br>**total length: 362626bp**<br>**GC level: 49.51%**<br>**bases masked: 255267 bp (70.39%))**			**sequence: 512**<br>**total length: 362626bp**<br>**GC level: 49.51%**<br>**bases masked: 246110 bp (67.87%))**			**sequence: 512**<br>**total length: 362626bp**<br>**GC level: 49.51%**<br>**bases masked: 225717 bp (62.25%))**		
**Repeat Types**	**Number of elements**	**Length occupied**	**Percentage of sequence**	**Number of elements**	**Length occupied**	**Percentage of sequence**	**Number of elements**	**Length occupied**	**Percentage of sequence**
SINEs:	9	784 bp	0.22%	15	1292 bp	0.36%	13	1935 bp	0.53%
–ALUs:	0	0 bp	0.00%	0	0 bp	0.00%	0	0 bp	0.00%
–MIRs:	0	0 bp	0.00%	0	0 bp	0.00%	5	839 bp	0.23%
LINEs:	163	119330 bp	32.91%	94	100795 bp	27.80%	84	88718 bp	24.47%
–LINE1:	0	0 bp	0.00%	0	0 bp	0.00%	0	0 bp	0.00%
–LINE2:	0	0 bp	0.00%	0	0 bp	0.00%	0	0 bp	0.00%
–L3/CR1:	163	119330 bp	32.91%	94	100795 bp	27.80%	84	88718 bp	24.47%
LTR elements:	68	78553 bp	21.94%	107	99403 bp	27.41%	109	85021 bp	23.45%
–ERVL	40	45157 bp	12.45%	55	52791 bp	14.56%	69	54457 bp	15.02%
–ERVL-MaLRs	0	0 bp	0.00%	0	0 bp	0.00%	0	0 bp	0.00%
–ERV_classI	7	9997 bp	2.76%	14	11837 bp	3.26%	16	14306 bp	3.95%
–ERV_classII	20	23453 bp	6.47%	38	34775 bp	9.59%	24	16258 bp	4.48%
DNA elements:	18	3974 bp	1.10%	21	7645 bp	2.11%	35	11014 bp	3.04%
–hAT-Charlie:	2	346 bp	0.10%	7	2249 bp	0.62%	5	4653 bp	1.28%
–TcMar-Tigger:	0	0 bp	0.00%	0	0 bp	0.00%	0	0 bp	0.00%
Unclassified:	8	8430 bp	2.32%	2	397 bp	0.11%	5	652 bp	0.18%
Total interspersed repeats:		212071 bp	58.48%		209532 bp	57.78%		187340 bp	51.66%
Small RNA:	39	6135 bp	1.69%	46	4770 bp	1.32%	2	380 bp	0.10%
Satellites:	5	5709 bp	1.57%	3	583 bp	0.16%	7	6199 bp	1.71%
Simple repeats:	197	31810 bp	8.77%	198	31808 bp	8.77%	200	32031 bp	8.83%
Low complexity:	3	147 bp	0.04%	3	147 bp	0.04%	3	147 bp	0.04%

′sequence′ (the number of fragments contained in the Gallus RepBase library). ′Base Masked (%)′ (the ratio of the bases in RepBase that can be covered by the detected fragments). ′GC(%)′ (the GC content). ′Length occupied′. (the length of the bases of the corresponding repetitive family in RepBase that can be covered by the detected fragments).

Supplementary Table S24. The proportion and detailed classification of elements in the RepBase library of Ant is covered by the detection results of LongRepMarker, RepeatScout, and RepeatModeler2.

	LongRepMarker			RepeatScout			RepeatModeler2		
sequence: 254				sequence: 254			sequence: 254		
total length: 214457bp				total length: 214457bp			total length: 214457bp		
GC level: 45.07%				GC level: 45.07%			GC level: 45.07%		
bases masked: 168915 bp (78.76%))				bases masked: 173383 bp (80.85%))			bases masked: 169070 bp (78.84%))		
Repeat Types	Number of elements	Length occupied	Percentage of sequence	Number of elements	Length occupied	Percentage of sequence	Number of elements	Length occupied	Percentage of sequence
SINEs:	1	69 bp	0.03%	0	0 bp	0.00%	0	0 bp	0.00%
–ALUs:	0	0 bp	0.00%	0	0 bp	0.00%	0	0 bp	0.00%
–MIRs:	0	0 bp	0.00%	0	0 bp	0.00%	0	0 bp	0.00%
LINEs:	19	8223 bp	3.83%	29	12137 bp	5.66%	16	13379 bp	6.24%
–LINE1:	0	0 bp	0.00%	0	0 bp	0.00%	0	0 bp	0.00%
–LINE2:	0	0 bp	0.00%	0	0 bp	0.00%	0	0 bp	0.00%
–L3/CR1:	0	0 bp	0.00%	0	0 bp	0.00%	0	0 bp	0.00%
LTR elements:	71	48344 bp	22.54%	43	49839 bp	23.24%	38	48684 bp	22.70%
–ERVL	0	0 bp	0.00%	0	0 bp	0.00%	0	0 bp	0.00%
–ERVL-MaLRs	0	0 bp	0.00%	0	0 bp	0.00%	0	0 bp	0.00%
–ERV_classI	0	0 bp	0.00%	0	0 bp	0.00%	0	0 bp	0.00%
–ERV_classII	0	0 bp	0.00%	0	0 bp	0.00%	0	0 bp	0.00%
DNA elements:	95	71724 bp	33.44%	111	72618 bp	33.86%	116	69591 bp	32.45%
–hAT-Charlie:	0	0 bp	0.00%	0	0 bp	0.00%	0	0 bp	0.00%
–TcMar-Tigger:	0	0 bp	0.00%	0	0 bp	0.00%	0	0 bp	0.00%
Unclassified:	39	9733 bp	4.54%	34	7607 bp	3.55%	13	7330 bp	3.42%
Total interspersed repeats:		138093 bp	64.39%		142201 bp	66.31%		138984 bp	64.81%
Small RNA:	6	566 bp	0.26%	7	746 bp	0.35%	0	0 bp	0.00%
Satellites:	0	0 bp	0.00%	0	0 bp	0.00%	0	0 bp	0.00%
Simple repeats:	184	30485 bp	14.21%	184	30449 bp	14.20%	181	30285 bp	14.12%
Low complexity:	2	110 bp	0.05%	2	110 bp	0.05%	2	110 bp	0.05%

′sequence′ (the number of fragments contained in the Ant RepBase library). ′Base Masked (%)′ (the ratio of the bases in RepBase that can be covered by the detected fragments). ′GC(%)′ (the GC content). ′Length occupied′. (the length of the bases of the corresponding repetitive family in RepBase that can be covered by the detected fragments).

Supplementary Table S25. The proportion and detailed classification of elements in the RepBase library of Human is covered by the detection results of *de novo* detection methods LongRepMarker, RepARK, and REPdenovo based on the NGS sequencing reads of the chromosome #14 of the human genome.

	LongRepMarker			RepARK			REPdenovo		
sequence: 1512				sequence: 1512			sequence: 1512		
total length: 1647075bp				total length: 1647075bp			total length: 1647075bp		
bases masked: 452080 bp (27.45%)				bases masked: 229636 bp (13.94%)			bases masked: 183245 bp (11.13%)		
Repeat Types	Num of elements	Length occupied	Percentage of sequence	Num of elements	Length occupied	Percentage of sequence	Num of elements	Length occupied	Percentage of sequence
**DNA elements:**	81	13426bp	0.82%	32	2828bp	0.17%	0	0bp	0.00%
–TcMar-Tigger:	40	8134bp	0.49%	23	2045bp	0.12%	0	0bp	0.00%
–hAT-Charlie:	23	3836bp	0.23%	5	461bp	0.03%	0	0bp	0.00%
**LINEs:**	345	191120bp	11.60%	544	118330bp	7.18%	126	116694bp	7.08%
–L3/CR1:	6	243bp	0.01%	1	75bp	0.00%	0	0bp	0.00%
–LINE1:	309	178143bp	10.82%	540	117752bp	7.15%	126	116694bp	7.08%
–LINE2:	10	2268bp	0.14%	3	503bp	0.03%	0	0bp	0.00%
**LTR elements:**	539	155596bp	9.45%	260	35630bp	2.16%	15	1427bp	0.09%
–ERVL:	119	36766bp	2.23%	30	3894bp	0.24%	0	0bp	0.00%
–ERVL-MaLRs:	70	12274bp	0.75%	49	8109bp	0.49%	15	1427bp	0.09%
–ERV-classI:	310	91588bp	5.56%	144	19917bp	1.21%	0	0bp	0.00%
–ERV-classII:	28	12635bp	0.77%	37	3710bp	0.23%	0	0bp	0.00%
**Low complexity:**	60	3018bp	0.18%	85	4170bp	0.25%	82	4030bp	0.24%
**SINEs:**	183	39215bp	2.38%	74	19956bp	1.21%	71	18201bp	1.11%
–ALUs:	173	38321bp	2.33%	70	19316bp	1.17%	71	18201bp	1.11%
–MIRs:	10	894bp	0.05%	4	640bp	0.04%	0	0bp	0.00%
**Satellites:**	14	3334bp	0.20%	19	1908bp	0.12%	6	524bp	0.03%
**Simple repeats:**	393	39165bp	2.38%	408	40077bp	2.43%	419	40550bp	2.46%
**Small RNA:**	0	0bp	0.00%	2	224bp	0.01%	0	0bp	0.00%
**Total interspersed repeats:**		407126bp	24.72%		183285bp	11.13%		138141bp	8.39%
**Unclassified:**	21	7769bp	0.47%	51	6541bp	0.40%	10	1819bp	0.11%

′sequence′ (the number of fragments contained in the Human RepBase library). ′Base Masked (%)′ (the ratio of the bases in RepBase that can be covered by the detected fragments). ′GC(%)′ (the GC content). ′Length occupied′. (the length of the bases of the corresponding repetitive family in RepBase that can be covered by the detected fragments).

Supplementary Table S26. The proportion and detailed classification of elements in the RepBase library of Human is covered by the detection results of *de novo* detection methods LongRepMarker, RepARK, and REPdenovo based on the NGS sequencing reads of the HG003_NA24149_father dataset.

	LongRepMarker			RepARK			REPdenovo		
sequence: 1512				sequence: 1512			sequence: 1512		
total length: 1647075bp				total length: 1647075bp			total length: 1647075bp		
bases masked: 1210784 bp (73.51%)				bases masked: 870210 bp (52.83%)			bases masked: 199536 bp (12.11%)		
Repeat Types	Num of elements	Length occupied	Percentage of sequence	Num of elements	Length occupied	Percentage of sequence	Num of elements	Length occupied	Percentage of sequence
**DNA elements:**	448	121106bp	7.35%	378	60164bp	3.65%	0	0bp	0.00%
–TcMar-Tigger:	126	37522bp	2.28%	129	23339bp	1.42%	0	0bp	0.00%
–hAT-Charlie:	143	39145bp	2.38%	121	17883bp	1.09%	0	0bp	0.00%
**LINEs:**	653	291629bp	17.71%	504	213163bp	12.94%	129	123854bp	7.52%
–L3/CR1:	26	4388bp	0.27%	5	1076bp	0.07%	0	0bp	0.00%
–LINE1:	586	278984bp	16.94%	481	209312bp	12.71%	129	123854bp	7.52%
–LINE2:	21	4123bp	0.25%	12	1992bp	0.12%	0	0bp	0.00%
**LTR elements:**	1082	620894bp	37.70%	2467	483315bp	29.34%	14	1927bp	0.12%
–ERVL:	219	92790bp	5.63%	336	68843bp	4.18%	0	0bp	0.00%
–ERVL-MaLRs:	102	23018bp	1.40%	93	26417bp	1.60%	14	1927bp	0.12%
–ERV-classI:	649	418886bp	25.43%	1713	320240bp	19.44%	0	0bp	0.00%
–ERV-classII:	63	76152bp	4.62%	309	65213bp	3.96%	0	0bp	0.00%
**Low complexity:**	16	652bp	0.04%	40	2039bp	0.12%	81	3926bp	0.24%
**SINEs:**	503	112306bp	6.82%	198	37531bp	2.28%	72	19991bp	1.21%
–ALUs:	469	108143bp	6.57%	162	34020bp	2.07%	72	19991bp	1.21%
–MIRs:	25	3428bp	0.21%	17	1893bp	0.11%	0	0bp	0.00%
**Satellites:**	39	9924bp	0.60%	102	16427bp	1.00%	11	1683bp	0.10%
**Simple repeats:**	234	32433bp	1.97%	318	36600bp	2.22%	414	40423bp	2.45%
**Small RNA:**	23	13124bp	0.80%	44	12839bp	0.78%	0	0bp	0.00%
**Total interspersed repeats:**		1165100bp	70.74%		806029bp	48.94%		153504bp	9.32%
**Unclassified:**	80	19165bp	1.16%	109	11856bp	0.72%	34	7732bp	0.47%

′sequence′ (the number of fragments contained in the Human RepBase library). ′Base Masked (%)′ (the ratio of the bases in RepBase that can be covered by the detected fragments). ′GC(%)′ (the GC content). ′Length occupied′. (the length of the bases of the corresponding repetitive family in RepBase that can be covered by the detected fragments).

Supplementary Table S27. The proportion and detailed classification of elements in the RepBase library of Human is covered by the detection results of *de novo* detection methods LongRepMarker, RepARK, and REPdenovo based on the NGS sequencing reads of the Mouse genome.

	LongRepMarker			RepARK			REPdenovo		
sequence: 1561				sequence: 1561			sequence: 1561		
total length: 1680566bp				total length: 1680566bp			total length: 1680566bp		
bases masked: 1167584 bp (69.48%)				bases masked: 867535 bp (51.62%)			bases masked: 381565 bp (22.70%)		
Repeat Types	Num of elements	Length occupied	Percentage of sequence	Num of elements	Length occupied	Percentage of sequence	Num of elements	Length occupied	Percentage of sequence
**DNA elements:**	395	69181bp	4.12%	40	8027bp	0.48%	0	0bp	0.00%
–TcMar-Tigger:	75	13659bp	0.81%	2	222bp	0.01%	0	0bp	0.00%
–hAT-Charlie:	145	28233bp	1.68%	27	6071bp	0.36%	0	0bp	0.00%
**LINEs:**	646	454590bp	27.05%	371	334128bp	19.88%	241	299948bp	17.85%
–L3/CR1:	21	2981bp	0.18%	0	0bp	0.00%	0	0bp	0.00%
–LINE1:	591	447010bp	26.60%	367	333804bp	19.86%	241	299948bp	17.85%
–LINE2:	22	3006bp	0.18%	4	324bp	0.02%	0	0bp	0.00%
**LTR elements:**	981	532020bp	31.66%	1795	450620bp	26.81%	118	31176bp	1.86%
–ERVL:	183	73304bp	4.36%	265	35675bp	2.12%	32	11265bp	0.67%
–ERVL-MaLRs:	156	31941bp	1.90%	102	23074bp	1.37%	43	10176bp	0.61%
–ERV-classI:	209	107341bp	6.39%	338	85642bp	5.10%	0	0bp	0.00%
–ERV-classII:	399	313254bp	18.64%	1086	305486bp	18.18%	43	9735bp	0.58%
**Low complexity:**	26	1069bp	0.06%	51	2566bp	0.15%	97	5116bp	0.30%
**SINEs:**	273	46075bp	2.74%	110	13784bp	0.82%	29	2553bp	0.15%
–ALUs:	164	31988bp	1.90%	56	7858bp	0.47%	24	1826bp	0.11%
–MIRs:	5	943bp	0.06%	4	589bp	0.04%	0	0bp	0.00%
**Satellites:**	10	3642bp	0.22%	22	4181bp	0.25%	2	734bp	0.04%
**Simple repeats:**	286	34991bp	2.08%	354	37830bp	2.25%	426	42038bp	2.50%
**Small RNA:**	41	14171bp	0.84%	46	12537bp	0.75%	0	0bp	0.00%
**Total interspersed repeats:**		1126788bp	67.05%		815147bp	48.50%		333677bp	19.86%
**Unclassified:**	172	24922bp	1.48%	63	8588bp	0.51%	0	0bp	0.00%

′sequence′ (the number of fragments contained in the Mouse RepBase library). ′Base Masked (%)′ (the ratio of the bases in RepBase that can be covered by the detected fragments). ′GC(%)′ (the GC content). ′Length occupied′. (the length of the bases of the corresponding repetitive family in RepBase that can be covered by the detected fragments).

Supplementary Table S28. The proportion and detailed classification of elements in the RepBase library of Human is covered by the detection results of *de novo* detection methods LongRepMarker, RepARK, and REPdenovo based on the NGS sequencing reads of the Ant genome.

	LongRepMarker			RepARK			REPdenovo		
**sequence: 254**				**sequence: 254**			**sequence: 254**		
**total length: 214457bp**				**total length: 214457bp**			**total length: 214457bp**		
**bases masked: 181755 bp (84.75%)**				**bases masked: 142209 bp (66.31%)**			**bases masked: 46235 bp (21.56%)**		
**Repeat Types**	Num of elements	Length occupied	Percentage of sequence	Num of elements	Length occupied	Percentage of sequence	Num of elements	Length occupied	Percentage of sequence
**DNA elements:**	108	73712bp	34.37%	261	62768bp	29.27%	21	14153bp	6.60%
–TcMar-Tigger:	0	0bp	0.00%	0	0bp	0.00%	0	0bp	0.00%
–hAT-Charlie:	0	0bp	0.00%	0	0bp	0.00%	0	0bp	0.00%
**LINEs:**	24	14161bp	6.60%	59	9312bp	4.34%	0	0bp	0.00%
–L3/CR1:	0	0bp	0.00%	0	0bp	0.00%	0	0bp	0.00%
–LINE1:	0	0bp	0.00%	0	0bp	0.00%	0	0bp	0.00%
–LINE2:	0	0bp	0.00%	0	0bp	0.00%	0	0bp	0.00%
**LTR elements:**	40	44578bp	20.79%	91	24272bp	11.32%	0	0bp	0.00%
–ERVL:	0	0bp	0.00%	0	0bp	0.00%	0	0bp	0.00%
–ERVL-MaLRs:	0	0bp	0.00%	0	0bp	0.00%	0	0bp	0.00%
–ERV-classI:	0	0bp	0.00%	0	0bp	0.00%	0	0bp	0.00%
–ERV-classII:	0	0bp	0.00%	0	0bp	0.00%	0	0bp	0.00%
**Low complexity:**	1	72bp	0.03%	2	137bp	0.06%	8	351bp	0.16%
**SINEs:**	0	0bp	0.00%	1	45bp	0.02%	0	0bp	0.00%
–ALUs:	0	0bp	0.00%	0	0bp	0.00%	0	0bp	0.00%
–MIRs:	0	0bp	0.00%	0	0bp	0.00%	0	0bp	0.00%
**Satellites:**	0	0bp	0.00%	0	0bp	0.00%	0	0bp	0.00%
**Simple repeats:**	185	30802bp	14.36%	194	30860bp	14.39%	208	31731bp	14.80%
**Small RNA:**	15	13646bp	6.36%	15	13826bp	6.45%	0	0bp	0.00%
**Total interspersed repeats:**		138052bp	64.37%		97695bp	45.55%		14153bp	6.60%
**Unclassified:**	11	5601bp	2.61%	17	1298bp	0.61%	0	0bp	0.00%

′sequence′ (the number of fragments contained in the Ant RepBase library). ′Base Masked (%)′ (the ratio of the bases in RepBase that can be covered by the detected fragments). ′GC(%)′ (the GC content). ′Length occupied′. (the length of the bases of the corresponding repetitive family in RepBase that can be covered by the detected fragments).

Supplementary Table S29. The proportion and detailed classification of elements in the RepBase library of Human is covered by the detection results of *de novo* detection methods LongRepMarker, RepARK, and REPdenovo based on the NGS sequencing reads of the Drosophila genome.

	LongRepMarker			RepARK			REPdenovo		
**sequence: 2489**				**sequence: 2489**			**sequence: 2489**		
**total length: 7220516bp**				**total length: 7220516bp**			**total length: 7220516bp**		
**bases masked: 3051295 bp (42.26%)**				**bases masked: 2820283 bp (39.06%)**			**bases masked: 199042 bp (2.76%)**		
**Repeat Types**	Num of elements	Length occupied	Percentage of sequence	Num of elements	Length occupied	Percentage of sequence	Num of elements	Length occupied	Percentage of sequence
**DNA elements:**	243	84091bp	1.16%	419	67480bp	0.93%	12	1758bp	0.02%
–TcMar-Tigger:	0	0bp	0.00%	0	0bp	0.00%	0	0bp	0.00%
–hAT-Charlie:	0	0bp	0.00%	0	0bp	0.00%	0	0bp	0.00%
**LINEs:**	1203	893842bp	12.38%	2304	790389bp	10.95%	189	74869bp	1.04%
–L3/CR1:	178	90613bp	1.25%	345	77798bp	1.08%	0	0bp	0.00%
–LINE1:	0	0bp	0.00%	0	0bp	0.00%	0	0bp	0.00%
–LINE2:	0	0bp	0.00%	0	0bp	0.00%	0	0bp	0.00%
**LTR elements:**	2328	1948634bp	26.99%	3444	1834947bp	25.41%	2	317bp	0.00%
–ERVL:	0	0bp	0.00%	0	0bp	0.00%	0	0bp	0.00%
–ERVL-MaLRs:	0	0bp	0.00%	0	0bp	0.00%	0	0bp	0.00%
–ERV-classI:	0	0bp	0.00%	0	0bp	0.00%	0	0bp	0.00%
–ERV-classII:	0	0bp	0.00%	0	0bp	0.00%	0	0bp	0.00%
**Low complexity:**	333	18139bp	0.25%	350	19015bp	0.26%	456	24145bp	0.33%
**SINEs:**	1	135bp	0.00%	0	0bp	0.00%	0	0bp	0.00%
–ALUs:	0	0bp	0.00%	0	0bp	0.00%	0	0bp	0.00%
–MIRs:	0	0bp	0.00%	0	0bp	0.00%	0	0bp	0.00%
**Satellites:**	20	4926bp	0.07%	36	4122bp	0.06%	6	1791bp	0.02%
**Simple repeats:**	1253	81143bp	1.12%	1288	82461bp	1.14%	1494	92425bp	1.28%
**Small RNA:**	15	13770bp	0.19%	20	13760bp	0.19%	5	593bp	0.01%
**Total interspersed repeats:**		2935431bp	40.65%		2702177bp	37.42%		80127bp	1.11%
**Unclassified:**	63	8729bp	0.12%	70	9361bp	0.13%	7	3183bp	0.04%

′sequence′ (the number of fragments contained in the Drosophila RepBase library). ′Base Masked (%)′ (the ratio of the bases in RepBase that can be covered by the detected fragments). ′GC(%)′ (the GC content). ′Length occupied′. (the length of the bases of the corresponding repetitive family in RepBase that can be covered by the detected fragments).

Supplementary Table S30. Detection results of *de novo* detection methods LongRepMarker and RepLong based on the SMS long reads of the Human and D.melanogaster genomes.

		Quast (length ≥ 5000bp)					Minimap2				RepeatMasker	
Species	Tool	Time(min)/Peak Mem(GB)	Max (kb)	N50 (kb)	N75 (kb)	N90 (kb)	0 time	1 time	>1 time	Mapping Rate (%)	Reference (%)	Repbase (%)
Human_wgs	LongRepMarker	97.155/20.613	28.880	10.919	8.232	6.342	0.03%	76.60%	23.37%	99.97%	NA	82.20%
	RepLong	1421.909/22.568	14.500	13.000	9.700	7.200	0.00%	91.11%	8.89%	100.0%	NA	17.51%
D.melanogaster	LongRepMarker	79.264/42.868	31.242	13.703	9.488	6.972	0.06%	69.88%	30.06%	99.94%	40.74%	44.32%
	RepLong	12696.500/71.808	14.600	8.000	5.600	4.300	0.00%	11.21%	88.79%	100.0%	21.31%	16.66%

The left sub-table shows the size statistics of detection results of each tool on various datasets, and the main evaluation indicators are Max(The longest contig), N50, N75, and N90. The middle sub-table shows the alignment ratio statistics of the detection results of LongRepMarker on various datasets, and the main evaluation indicators are ′0 time (The proportion of fragments in detection results that can not be aligned to the reference genome)′, ′1 time (The proportion of fragments in detection results that can be aligned to the reference genome only one location)′, ′ >1 time (The proportion of fragments in detection results that can be aligned to the reference genome many locations)′ and ′Mapping rate(%) (The overall proportion of fragments in detection results that can be aligned to the reference genome)′. The right sub-table shows the proportion of repetitive fragments in reference genome or repbase library that can be covered by the detection results. ′Time(min)/Peak Mem(GB)′ represents the run time and peak memory consumption.

Supplementary Table S31. Detection results of *de novo* detection methods LongRepMarker and RepLong based on the SMS long reads of the Human and D.melanogaster genomes covering the masked repeats on the corresponding reference genomes.

	Marking on reference genome					Repeat Classification			
Tools	Species	Num	Total length (kb)	GC level (%)	Base Masked (%)	Repeat type	Num	Length Occupied	Percentage of sequence
LongRepMarker	Human_wgs	455	3209286105.105	40.99%	NA%	Interspersed	NA	NA kb	NA%
		455	3209286105.105	40.99%	NA%	Simple	NA	NA kb	NA%
	D.melanogaster	15	168736.537	41.74%	40.74%	Interspersed	99811	67240.060kb	39.85%
		15	168736.537	41.74%	40.74%	Simple	83075	4030.411kb	2.39%
RepLong	Human_wgs	455	3209286105.105	40.99%	NA %	Interspersed	NA	NA kb	NA %
		455	3209286105.105	40.99%	NA %	Simple	NA	NA kb	NA %
	D.melanogaster	15	168736.537	41.74%	21.31%	Interspersed	331169	59908.910kb	20.24%
		15	168736.537	41.74%	21.31%	Simple	191168	8911.109kb	3.01%

The left sub-table shows the statistics of detection results covering the corresponding reference genome, and the right sub-table shows the statistics of repeat classification. ′Num′ indicates the number of fragments in detection results. ′Base Masked (%)′ indicates the coverage ratio of the reference genome. ′GC(%)′ indicates the GC content.

Supplementary Table S32. Detection results of *de novo* detection methods LongRepMarker and RepLong based on the SMS long reads of the Human and D.melanogaster genomes covering the elements in the corresponding RepBase libraries.

	Marking on repbase libray					Repeat Classification			
Tools	Species	Num	Total length (kb)	GC level (%)	Base Masked (%)	Repeat type	Num	Length Occupied	Percentage of sequence
LongRepMarker	Human_wgs	1381	1438.717kb	44.94%	82.20%	Interspersed	3191	1307.883kb	90.91%
		1381	1438.717kb	44.94%	82.20%	Simple	134	7.593kb	0.53%
	D.melanogaster	2383	7197.137kb	42.75%	44.32%	Interspersed	7142	3558.226kb	49.44%
		2383	7197.137kb	42.75%	44.32%	Simple	1424	68.316kb	0.95%
RepLong	Human_wgs	1381	1438.717kb	44.94%	17.51%	Interspersed	647	249.659kb	17.35%
		1381	1438.717kb	44.94%	17.51%	Simple	355	17.355kb	1.21%
	D.melanogaster	2383	7197.137kb	42.75%	16.66%	Interspersed	1165	1159.780kb	16.11%
		2383	7197.137kb	42.75%	16.66%	Simple	1799	87.913kb	1.22%

The left sub-table shows the statistics of detection results covering the corresponding reference genome, and the right sub-table shows the statistics of repeat classification. ′Num′ indicates the number of fragments in detection results. ′Base Masked (%)′ indicates the coverage ratio of reference genome. ′GC(%)′ indicates the GC content.

Supplementary Note 7.3 Performance analysis of automated repeat sequence classification and masking methods

The performance and composition analysis of the four most important databases (RepBase, Dfam, RepeatDB, REXdb, and msRepDB) in the field of repetitive sequences identification is performed in this study, and the detailed evaluation results are shown in **Supplementary Tables S33 to S41**. Furthermore, the performance of the four most famous classification methods (TERL, PASTEC, TEclass, TEsorter, RepeatClssifier, LTR_Retriver, LTR_Classifier, and DeepTE) are compared in **Supplementary Tables S42 to S46**.

Supplementary Note 7.3.1 Datebase used in repeat sequence automated classification and masking An accurate and complete repeat database is essential to achieve the accurate automated classification and annotation of repeats in genomes. Three well-known repetitive sequence nucleic acid libraries exist, namely RepBase [144], Dfam [145] and msRepDB [146]. In addition, three well-known repetitive sequence protein libraries exist, namely RepeatsDB [147], REXdb [148] and Pfam [149]. The details of these libraries are described as follows.

The RepBase database (https://www.girinst.org/repbase/) is one of the most famous repeat-sequence databases and contains prototypical sequences for repetitive DNA from many eukaryotic species. Most of RepBase's prototypic sequences are consensus sequences of significant families and subfamilies of repeats. The RepBase update currently contains more than 38,000 sequences of different families or subfamilies. In addition, Repbase is used as a reference library for masking and annotating repetitive DNA for some tools, such as RepeatMasker and Censor, and it has been widely used in genome sequencing projects worldwide. Next, the Dfam (https://www.dfam.org/releases/Dfam_3.5/) database is an open collection of TE DNA sequence alignments, HMMs, consensus sequences, and genome annotations. The latest version of the Dfam library houses 285,542 TE models across 595 species, and it has been incorporated into the new version of RepeatMasker. Moreover, msRepDB (https://msrepdb.cbrc.kaust.edu.sa/pages/msRepDB/index.html) is constructed based on the hybrid detection framework LongRepMarker [174]. It contains more than 84,000 species and is currently the most comprehensive cross-species repeat sequence database.

The RepeatsDB database (https://repeatsdb.bio.unipd.it/) collects protein structures of annotated TRs. This database provides the unit position, classification, and reference to other databases. The current version of RepeatsDB is based on an update of RepeatsDB-lite [190], a method for automatically identifying repetitive units in protein structures. The Pfam (http://pfam.xfam.org/) database contains many protein families, each of which is represented by MSAs and HMMs. The latest version of Pfam is v.35.0, which contains 19,632 families and clans constructed by the European Bioinformatics Institute (EMBL-EBI, https://www.ebi.ac.uk/) based on UniProt release 2021_03 (https://www.uniprot.org/), and a sequence database called Pfamseq. The REXdb database (http://repeatexplorer.org/?page_id=918) is a reference for TE protein domains. In addition, REXdb is employed in the repeat analysis tools RepeatExplorer2 [191] and DANTE [192], which are available on the Galaxy server (https://repeatexplorer-elixir.cerit-sc.cz/). The classification table and protein sequences are two files in the database archive. Composition and performance analysis of the five most essential databases (RepBase, Dfam, RepeatDB, REXdb, and msRepDB) used in the field of repetitive sequences identification and classification is performed in **Supplementary Tables S33 to S41**.

Supplementary Table S33. Partial comparison of the length distribution, multiple alignment ratio, proportion of covering the reference genome and duplication ratio of elements contained in msRepDB and Dfam databases.

		**Length distribution**					**Mapping**		**RepeatMasker**	**Other**
Species	Database	Num	Max (bp)	N50 (bp)	N75 (bp)	N95 (bp)	MAR (%)	Non-MAR (%)	Reference (%)	Duplication ratio (%)
H.sapiens(human)	msRepDB	1,628	20,016	2,954	920	492	**82.58%**	**17.41%**	**47.36%**	**0.11%**
	Dfam+RepBase	1,353	9,043	2,532	786	464	80.93%	19.06%	45.62%	0.15%
Mouse	msRepDB	1,792	15,041	3,958	1,145	513	**88.74%**	**11.25%**	**43.26%**	**0.15**%
	Dfam+RepBase	1,407	8,959	2,210	791	437	86.28%	13.71%	40.58%	0.21%
Oryza sativa(Rice)	msRepDB	3,564	13,922	3,712	1,744	810	**85.17%**	**14.82%**	**50.65%**	**4.14%**
	Dfam+RepBase	3,049	20,789	3,879	1,831	892	82.81%	17.18%	50.50%	3.91%
D.melanogaster	msRepDB	510	20,014	4,470	2,010	978	**97.77%**	**2.22%**	**22.03%**	**2.41%**
	Dfam+RepBase	258	15,576	4,802	3,204	1,036	89.77%	10.22%	20.85%	3.36%
Glycine max	msRepDB	1,245	10,856	4,579	3,498	1,408	**95.72%**	**4.27%**	**41.58%**	**0.46%**
	Dfam+RepBase	596	17,080	4,688	4,180	3,207	90.45%	9.54%	36.11%	0.53%

′Num′ represents the number of fragments contained in the database. ′Max(bp)′ represents the length of the longest fragment in the database. ′N50′ represents the length of a fragment, such that all the fragments of at least the same length together cover at least 50% of the total length of all fragments contained in the database. ′N75′ represents the length of a fragment, such that all the fragments of at least the same length together cover at least 75% of the total length of all fragments contained in the database. ′N95′ represents the length of a fragment, such that all the fragments of at least the same length together cover at least 95% of the total length of all fragments contained in the database. ′MAR(%) and Non-MAR(%)′ respectively represent the ratios of multiple alignment and non-multiple alignment. ′Reference(%)′ represents the proportion of covering the reference genome. ′Duplication ratio′ represents the total number of aligned bases in the repetitive sequences divided by the total number of those in the reference. If there are too many repetitive sequences that cover the same regions, the duplication ratio will be greatly increased. This occurs due to multiple reasons, including overestimating repeat multiplicities and overlaps between repetitive sequences.

Supplementary Table S34. Partial comparison of the proportion and detailed classification of detected repeats generated based on two databases of the Human genome.

**Combination of RepBase and Dfam** [**Sequences: 639; Total length: 3,272,089,205bp; GC level: 41.04%; Bases masked: 45.62%**]				**msRepDB** [**Sequences: 639; Total length: 3,272,089,205bp; GC level: 41.04%; Bases masked: 47.36%**]		
Repeat Types	Number of elements	Length occupied	Percentage of sequences	Number of elements	Length occupied	Percentage of sequences
Retroelements:	2,800,814	1,236,215,277bp	37.78%	3,921,320	1,297,267,059bp	39.65%
+SINEs:	1,453,130	369,205,643bp	11.28%	1,599,106	321,120,861bp	9.81%
+Penelope:	75	14,277bp	0.00%	75	14,225bp	0.00%
+LINEs:	807,771	588,058,432bp	17.97%	1,671,568	702,653,923bp	21.47%
++CRE/SLACS:	0	0bp	0.00%	0	0bp	0.00%
+++L2/CR1/Rex:	193,908	56,822,264bp	1.74%	289,067	68,581,491bp	2.10%
+++R1/LOA/Jockey:	0	0bp	0.00%	0	0bp	0.00%
+++R2/R4/NeSL:	399	95,545bp	0.00%	400	95,165bp	0.00%
+++RTE/Bov-B:	9,890	2,788,967bp	0.09%	9,885	2,771,441bp	0.08%
+++L1/CIN4:	603,337	528,287,954bp	16.15%	1,371,979	631,142,544bp	19.29%
+LTR elements:	539,913	278,951,202bp	8.53%	650,646	273,492,275bp	8.36%
++BEL/Pao:	0	0bp	0.00%	0	0bp	0.00%
++Tyl/Copia:	0	0bp	0.00%	12	3,718bp	0.00%
++Gypsy/DTRS1:	14,309	3,767,626bp	0.12%	15,114	3,748,839bp	0.11%
+++Retroviral:	515,395	272,547,814bp	8.33%	625,198	267,126,378bp	8.16%
DNA transposons	425,304	102,360,429bp	3.13%	424,099	100,536,165bp	3.07%
+hobo-Activator:	280,952	57,692,527bp	1.76%	279,963	56,931,920bp	1.74%
+Tc1-IS630-Pogo:	128,851	41,753,772bp	1.28%	128,405	40,705,394bp	1.24%
+En-Spm:	0	0bp	0.00%	0	0bp	0.00%
+MuDR-IS905:	0	0bp	0.00%	0	0bp	0.00%
+PiggyBac:	2,310	554,582bp	0.02%	2,282	546,321bp	0.02%
+Tourist/Harbinger:	321	59,199bp	0.00%	320	59,104bp	0.00%
+Other:	0	0bp	0.00%	0	0bp	0.00%
Rolling circles	1614	402,976bp	0.01%	3,647	1,041,776bp	0.03%
Unclassified	122,691	24,233,010bp	0.74%	206,770	27,820,419bp	0.85%
Total interspersed repeats		1,362,808,716bp	41.65%		1,425,623,643bp	43.57%
Small RNA	12,650	1,358,026bp	0.04%	10,133	977,808bp	0.03%
Satellites	15,404	82,714,065bp	2.53%	11,997	79,154,876bp	2.42%
Simple repeats	710,220	39,030,544bp	1.19%	656,920	37,245,405bp	1.14%
Low complexity	102,465	6,353,924bp	0.19%	92,216	5,545,284bp	0.17%

*The test results are obtained by using RepeatMasker based on the msRepDB database and the combination of Dfam and RepBase, respectively, under the default parameter settings.

Supplementary Table S35. Partial comparison of the proportion and detailed classification of detected repeats generated based on two databases of the Drosophila genome.

**Combination of RepBase and Dfam** [**Sequences: 1,870; Total length: 143,726,002bp; GC level: 42.01%; Bases masked: 20.85%**]				**msRepDB** [**Sequences: 1,870; Total length: 143,726,002bp; GC level: 42.01%; Bases masked: 22.03%**]		
Repeat Types	Number of elements	Length occupied	Percentage of sequences	Number of elements	Length occupied	Percentage of sequences
Retroelements:	15,330	21,048,835bp	14.65%	23,352	22,594,349bp	15.72%
+SINEs:	0	0bp	0.00%	0	0bp	0.00%
+Penelope:	0	0bp	0.00%	0	0bp	0.00%
+LINEs:	5,293	5,447,560bp	4.49%	6,438	6,580,002bp	4.58%
++CRE/SLACS:	0	0bp	0.00%	0	0bp	0.00%
+++L2/CR1/Rex:	811	844,019bp	0.59%	868	841,748bp	0.59%
+++R1/LOA/Jockey:	1014	1,562,240bp	1.09%	1,991	2,357,332bp	1.64%
+++R2/R4/NeSL:	38	39,896bp	0.03%	38	39,900bp	0.03%
+++RTE/Bov-B:	0	0bp	0.00%	0	0bp	0.00%
+++L1/CIN4:	0	0bp	0.00%	0	0bp	0.00%
+LTR elements:	10,037	14,601,275bp	10.16%	16,914	16,014,347bp	11.14%
++BEL/Pao:	2,326	3,123,105bp	2.17%	2,932	3,118,279bp	2.17%
++Tyl/Copia:	500	740,782bp	0.52%	783	733,414bp	0.51%
++Gypsy/DTRS1:	7,211	10,737,388bp	7.47%	13,111	12,139,653bp	8.45%
+++Retroviral:	0	0bp	0.00%	0	0bp	0.00%
DNA transposons	4,135	1,870,086bp	1.30%	4,534	1,868,020bp	1.30%
+hobo-Activator:	189	75,919bp	0.05%	168	76,228bp	0.05%
+Tc1-IS630-Pogo:	1,112	609,344bp	0.42%	1,126	596,800bp	0.42%
+En-Spm:	0	0bp	0.00%	0	0bp	0.00%
+MuDR-IS905:	0	0bp	0.00%	0	0bp	0.00%
+PiggyBac:	23	8,619bp	0.01%	23	8,611bp	0.01%
+Tourist/Harbinger:	0	0bp	0.00%	0	0bp	0.00%
+Other:	2,243	913,674bp	0.64%	2,454	893,743bp	0.62%
Rolling circles	4662	999,082bp	0.70%	5,225	1,022,538bp	0.71%
Unclassified	495	78,825bp	0.05%	885	211,424bp	0.15%
Total interspersed repeats		22,997,746bp	16.00%		24,673,793bp	17.17%
Small RNA	306	86,258bp	0.06%	280	95,863bp	0.07%
Satellites	1,372	1,804,199bp	1.26%	1,828	1,862,604bp	1.30%
Simple repeats	85,083	3,589,418bp	2.50%	83,742	3,522,748bp	2.45%
Low complexity	10,443	488,602bp	0.34%	10,307	481,694bp	0.34%

*The test results are obtained by using RepeatMasker based on the msRepDB database and the combination of Dfam and RepBase, respectively, under the default parameter settings.

Supplementary Table S36. Partial comparison of the proportion and detailed classification of detected repeats generated based on two databases of the Glycine max genome.

**The combination of RepBase and Dfam** [**Sequences: 284; Total length: 978,941,695bp; GC level: 34.74%; Bases masked: 36.11%**]				**msRepDB** [**Sequences: 284; Total length: 978,941,695bp; GC level: 34.74%; Bases masked: 41.58%**]		
Repeat Types	Number of elements	Length occupied	Percentage of sequences	Number of elements	Length occupied	Percentage of sequences
Retroelements:	199,220	289,032,002bp	29.52%	244,640	328,757,414bp	33.58%
+SINEs:	0	0bp	0.00%	0	0bp	0.00%
+Penelope:	0	0bp	0.00%	0	0bp	0.00%
+LINEs:	12,626	10,304,690bp	1.05%	13,156	10,432,965bp	1.07%
++CRE/SLACS:	0	0bp	0.00%	0	0bp	0.00%
+++L2/CR1/Rex:	0	0bp	0.00%	0	0bp	0.00%
+++R1/LOA/Jockey:	0	0bp	0.00%	0	0bp	0.00%
+++R2/R4/NeSL:	0	0bp	0.00%	0	0bp	0.00%
+++RTE/Bov-B:	3,790	2,001,199bp	0.20%	3,945	2,017,968bp	0.21%
+++L1/CIN4:	8,836	8,303,491bp	0.85%	9,211	8,414,997bp	0.86%
+LTR elements:	186,594	278,727,312bp	28.47%	231,484	318,324,449bp	32.52%
++BEL/Pao:	0	0bp	0.00%	0	0bp	0.00%
++Tyl/Copia:	58,199	80,563,666bp	8.23%	82,522	88,004,365bp	8.99%
++Gypsy/DTRS1:	126,690	195,309,037bp	19.95%	141,484	225,436,017bp	23.03%
+++Retroviral:	0	0bp	0.00%	340	206,126bp	0.02%
DNA transposons	58,468	41,514,301bp	4.24%	61,037	42,777,718bp	4.37%
+hobo-Activator:	7,612	2,233,822bp	0.23%	5,901	1,964,862bp	0.20%
+Tc1-IS630-Pogo:	117	56,379bp	0.01%	321	75,504bp	0.01%
+En-Spm:	0	0bp	0.00%	0	0bp	0.00%
+MuDR-IS905:	0	0bp	0.00%	0	0bp	0.00%
+PiggyBac:	0	0bp	0.00%	0	0bp	0.00%
+Tourist/Harbinger:	923	564,171bp	0.06%	1,070	589,379bp	0.06%
+Other:	0	0bp	0.00%	0	0bp	0.00%
Rolling circles	538	252,405bp	0.03%	967	740,463bp	0.08%
Unclassified	0	0bp	0.00%	46,069	9,184,163bp	0.94%
Total interspersed repeats		330,546,303bp	33.77%		380,719,295bp	38.89%
Small RNA	2,223	902,022bp	0.09%	2,221	901,833bp	0.09%
Satellites	19,885	2,175,759bp	0.22%	9,389	6,367,993bp	0.65%
Simple repeats	323,670	15,236,633bp	1.56%	306,680	14,384,955bp	1.47%
Low complexity	82,139	4,344,053bp	0.44%	75,614	3,960,136bp	0.40%

*The test results are obtained by using RepeatMasker based on the msRepDB database and the combination of Dfam and RepBase, respectively, under the default parameter settings.

Supplementary Table S37. Partial comparison of the proportion and detailed classification of detected repeats generated based on two databases of the Rice genome.

**The combination of RepBase and Dfam** [**Sequences: 61; Total length: 374,424,240bp; GC level: 43.57%; Bases masked: 50.50%**]				**msRepDB** [**Sequences: 61; Total length: 374,424,240bp; GC level: 43.57%; Bases masked: 50.65%**]		
Repeat Types	Number of elements	Length occupied	Percentage of sequences	Number of elements	Length occupied	Percentage of sequences
Retroelements:	65,791	95,531,185bp	25.51%	79,315	95,506,323bp	25.51%
+SINEs:	6,826	987,304bp	0.26%	6,867	952,864bp	0.25%
+Penelope:	0	0bp	0.00%	0	0bp	0.00%
+LINEs:	11,557	5,568,202bp	1.49%	11,562	5,572,111bp	1.49%
++CRE/SLACS:	0	0bp	0.00%	0	0bp	0.00%
+++L2/CR1/Rex:	0	0bp	0.00%	0	0bp	0.00%
+++R1/LOA/Jockey:	0	0bp	0.00%	0	0bp	0.00%
+++R2/R4/NeSL:	0	0bp	0.00%	0	0bp	0.00%
+++RTE/Bov-B:	0	0bp	0.00%	0	0bp	0.00%
+++L1/CIN4:	10,365	5,077,865bp	1.36%	10,381	5,087,940bp	1.36%
+LTR elements:	47,408	88,975,679bp	23.76%	60,886	88,981,348bp	23.76%
++BEL/Pao:	0	0bp	0.00%	0	0bp	0.00%
++Tyl/Copia:	10,831	14,340,045bp	3.83%	14,004	14,335,288bp	3.83%
++Gypsy/DTRS1:	32,899	73,328,202bp	19.58%	42,849	73,361,406bp	19.59%
+++Retroviral:	0	0bp	0.00%	0	0bp	0.00%
DNA transposons	241,722	68,736,938bp	18.36%	248,589	69,123,767bp	18.46%
+hobo-Activator:	29,293	6,598,030bp	1.76%	29,091	6,573,553bp	1.76%
+Tc1-IS630-Pogo:	40.793	7,245,966bp	1.94%	43,607	7,258,626bp	1.94%
+En-Spm:	0	0bp	0.00%	0	0bp	0.00%
+MuDR-IS905:	0	0bp	0.00%	0	0bp	0.00%
+PiggyBac:	0	0bp	0.00%	0	0bp	0.00%
+Tourist/Harbinger:	51,501	10,987,662bp	2.93%	52,626	11,058,599bp	2.95%
+Other:	58	7,292bp	0.00%	58	7,292bp	0.00%
Rolling circles	66,680	17,453,430bp	4.66%	66,425	17,410,443bp	4.65%
Unclassified	4,534	1,574,152bp	0.42%	5,066	1,732,111bp	0.46%
Total interspersed repeats		165,842,275bp	44.29%		166,362,201bp	44.43%
Small RNA	4,631	704,192bp	0.19%	4,997	762,938bp	0.20%
Satellites	426	1,368,174bp	0.37%	591	1,382,862bp	0.37%
Simple repeats	88,676	3,867,177bp	1.03%	88,603	3,878,911bp	1.04%
Low complexity	9,277	456,471bp	0.12%	9,235	454,107bp	0.12%

*The test results are obtained by using RepeatMasker based on the msRepDB database and the combination of Dfam and RepBase, respectively, under the default parameter settings.

Supplementary Table S38. Partial comparison of the proportion and detailed classification of detected repeats generated based on two databases of the Mouse genome.

**The combination of RepBase and Dfam** [**Sequences: 61; Total length: 2,728,222,451bp; GC level: 41.67%; Bases masked: 40.58%**]				**msRepDB** [**Sequences: 61; Total length: 2,728,222,451bp; GC level: 41.67%; Bases masked: 43.26%**]		
Repeat Types	Number of elements	Length occupied	Percentage of sequences	Number of elements	Length occupied	Percentage of sequences
Retroelements:	2,604,809	985,247,550bp	36.11%	3,497,950	1,065,736,604bp	39.06%
+SINEs:	1,211,566	162,662,859bp	5.96%	1,293,615	162,373,734bp	5.95%
+Penelope:	34	6,243bp	0.00%	34	6,243bp	0.00%
+LINEs:	623,172	523,121,773bp	19.17%	1,181,500	583,037,969bp	21.37%
++CRE/SLACS:	0	0bp	0.00%	0	0bp	0.00%
+++L2/CR1/Rex:	13,069	2,187,962bp	0.08%	13,330	2,187,279bp	0.08%
+++R1/LOA/Jockey:	0	0bp	0.00%	0	0bp	0.00%
+++R2/R4/NeSL:	92	18,578bp	0.00%	82	17,994bp	0.00%
+++RTE/Bov-B:	1,195	223,045bp	0.01%	1,194	222,866bp	0.01%
+++L1/CIN4:	608,739	520,675,766bp	19.08%	1,166,817	580,593,408bp	21.28%
+LTR elements:	770,071	299,462,918bp	10.98%	1,022,835	320,324,901bp	11.74%
++BEL/Pao:	0	0bp	0.00%	0	0bp	0.00%
++Tyl/Copia:	0	0bp	0.00%	0	0bp	0.00%
++Gypsy/DTRS1:	1,058	176,553bp	0.01%	1,069	176,306bp	0.01%
+++Retroviral:	767,530	298,945,097bp	10.96%	1,020,331	319,857,078bp	11.72%
DNA transposons	101,050	19,397,414bp	0.71%	101,523	19,514,699bp	0.72%
+hobo-Activator:	80,289	15,218,974bp	0.56%	81,366	15,449,005bp	0.57%
+Tc1-IS630-Pogo:	17,991	3,780,493bp	0.14%	17,390	3,668,389bp	0.13%
+En-Spm:	0	0bp	0.00%	0	0bp	0.00%
+MuDR-IS905:	0	0bp	0.00%	0	0bp	0.00%
+PiggyBac:	166	40,102bp	0.00%	166	39,950bp	0.00%
+Tourist/Harbinger:	161	25,353bp	0.00%	160	25,316bp	0.00%
+Other:	0	0bp	0.00%	0	0bp	0.00%
Rolling circles	180	31,909bp	0.00%	180	31,865bp	0.00%
Unclassified	125,730	15,031,702bp	0.55%	189,664	24,656,083bp	0.90%
Total interspersed repeats		1,019,676,666bp	37.38%		1,109,907,386bp	40.68%
Small RNA	16,041	1,313,388bp	0.05%	8,468	696,855bp	0.03%
Satellites	69,015	8,721,290bp	0.32%	29,094	4,768,705bp	0.17%
Simple repeats	1,319,791	67,604,107bp	2.48%	1,148,193	57,689,551bp	2.11%
Low complexity	147,721	9,696,829bp	0.36%	114,082	7,077,301bp	0.26%

*The test results are obtained by using RepeatMasker based on the msRepDB database and the combination of Dfam and RepBase, respectively, under the default parameter settings.

Supplementary Table S39. TE reference sequences of 23 genomes with about 39,039 TE consensus are collected in the RepetDB database.

Species	Genome assembly annotated (without gap)	Cumulative coverage	Genome coverage	No. of consensus sequences	No. of genome copies	No. of full-length genome copies
Arabidopsis lyrata	206,667,935	76,899,516	37.21	2,408	112,563	9,527
Arabidopsis thaliana	119,146,348	22,954,742	19.27	641	37,129	2,513
Arabis alpina	309,171,870	152,175,264	49.22	3,204	268,936	11,729
Brassica rapa	283,841,084	101,457,103	35.74	2,660	239,373	10,881
Capsella rubella	134,834,574	27,975,436	20.75	873	54,560	3,326
Schrenkiella parvula	123,600,562	19,838,473	16.05	455	37,597	1,356
Fragaria vesca	211,673,467	58,062,323	27.43	1,543	112,822	8,576
Malus domestica	624,851,326	365,363,669	58.47	2,456	564,270	25,280
Prunus persica	227,411,381	99,590,159	43.79	1,738	170,681	9,056
Pyrus communis	577,335,413	194,166,715	33.63	975	482,345	11,435
Vitis vinifera	486,205,130	290,981,308	59.85	2,473	475,119	10,551
Triticum aestivum	986,092,508	894,245,831	90.69	6,671	785,986	15,905
Zea mays	2,059,701,728	1,768,705,851	85.87	7,319	1,381,303	41,666
Blumeria graminis hordei	87,976,437	59,069,666	67.14	733	122,756	8,909
Botrytis cinerea B0510	42,630,066	1,583,714	3.72	15	1,927	263
Botrytis cinerea T4	37,887,365	254,124	0.67	24	611	62
Colletotrichum higginsianum	50,819,261	3,505,545	6.90	41	1,482	440
Magnaporthe oryzae	40,949,321	4,549,294	11.11	37	4,358	463
Melampsora larici populina	97,682,699	49,975,736	51.16	1,779	88,708	6,942
Microtryum violaceum	25,201,507	4,423,374	17.55	286	9,620	640
Puccinia graminis	81,521,292	37,620,112	46.15	1,625	6,9167	6,648
Sclerotinia sclerotiorum	38,001,451	3,459,261	9.10	178	13,868	622
Tuber melanosporum	123,533,734	73,821,108	59.76	905	72,212	3,845

Supplementary Table S40. The types of transposon elements in eukaryotic genomes collected in the RepBase database.

Type of TE	Super-family
DNA transposon	Academ, Crypton (CryptonA, CryptonF, CryptonI, CryptonS, CryptonV), Dada, EnSpm/CACTA, Ginger1, Ginger2, Harbinger, hAT, Helitron, IS3EU, ISL2EU, Kolobok, Mariner/Tc1, Merlin, MuDR, Novosib, P, piggyBac, Polinton, Sola (Sola1, Sola2, Sola3), Transib, Zator, Zisupton
LTR retrotransposon	BEL, Copia, DIRS, Gypsy, ERV1, ERV2, ERV3, ERV4, Lentivirus
Non-LTR retrotransposon	Ambal a, CR1, CRE, Crack, Daphne, Hero, I, Ingi, Jockey, Kiri a, L1, L2, L2A, L2B, Loa, NeSL, Nimb, Outcast, Penelope, Proto1, Proto2, R1, R2, R4, RandI/Dualen, Rex1, RTE, RTETP, RTEX, Tad1, Tx1, Vingi
	SINE (SINE1/7SL, SINE2/tRNA, SINE3/5S, SINE4, SINEU)

Supplementary Table S41. The types of transposon elements in eukaryotic genomes collected in the REXdb database.

Type of TE	Super-family	Family	Sub-family
Class I	SINE		
	LTR	Ty1	copia, Ale, Alesia, Angela, Bianca, Bryco, Lyco, Gymco-III, Gymco-I, Gymco-II, Ikeros, Ivana, Gymco-IV, Osser, SIRE, TAR, Tork, Ty1-outgroup
		Ty3	gypsy, non-chromovirus, non-chromo-outgroup, Phygy, Selgy, OTA, Athila, Tat, TatI, TatII, TatIII, Ogre, Retand, chromovirus, Chlamyvir, Tcn1, chromo-outgroup, CRM, Galadriel, Tekay, Reina, chromo-unclass
	pararetrovirus		
	DIRS		
	Penelope		
	LINE		
Class II	Subclass_1	TIR, MITE, EnSpm, CACTA, hAT, Kolobok, Merlin, MuDR, Mutator, Novosib , P, PIF , Harbinger, PiggyBac, Sola1, Sola2, Tc1, Mariner	
	Subclass_2	Helitron	

Supplementary Note 7.3.2 Comparison of the automated classification and masking methods
Comparison performance of the four most famous classification methods (TERL, PASTEC, TEclass, and DeepTE) is performed based on three datasets (**Supplementary Table S42**), and evaluation results are shown in **Tables S43 to S45**. Data from RepBase and PGSB database are combined to train the DeepTE models. Among them, dataset #1 consists of orders (LTR and LINE) and class (Class II) from RepBase consensus sequences and generated non-TE sequences, dataset #2 consists of orders (LTR, LINE, and SINE) and class (Class II) from the seven databases and non-TE sequences, and dataset #3 are sampled from orders consensus sequences from RepBase database and undersampled to 2850, which is the total sequence of the class with the least total sequences on RepBase (i.e., LINE). Furthermore, we compared the performance of LTR elements classification among six classifiers (DeepTE, TERL, TEsorter, RepeatClassifier, LTR_Retriever, and LTR_Classifier) on Rice and Maize genomes (**Supplementary Tables S46**).

Supplementary Table S42. The datasets used in evaluation of classification methods.

Dataset	Superfamily	DPTE	PGSB	RepBase	RiTE	SPTE	TEfam	TREP	Total
#1	LTR	-	-	-	-	-	-	-	24,505
	LINE	-	-	-	-	-	-	-	2,850
	Class II	-	-	-	-	-	-	-	9,623
	Non-TE	-	-	-	-	-	-	-	-
#2	LTR	10,370	11,192	24,505	77,380	9,574	1,271	943	135,235
	LINE	1,299	470	2,850	784	278	368	8	6057
	SINE	0	191	685	3,072	0	0	0	3,948
	Class II	260	1,150	9,623	150,142	59	128	996	162,358
#3	LTR	10,370	11,192	24,505	77,380	9,574	1,271	943	110,730
	LINE	1,299	470	2,850	784	278	368	8	3,207
	Class II	260	1,150	9,623	150,142	59	128	996	152,735

Supplementary Table S43. Performance comparison of different classification methods on dataset #1.

Class	Methods	Accuracy	precision	Recall	Specificity	F1-score
LTR	TERL	0.947 ± 0.008	0.895 ± 0.029	0.896 ± 0.026	0.965 ± 0.011	0.895 ± 0.015
	PASTEC	0.984	0.998	0.939	0.999	0.967
	Teclass	0.911	0.739	0.995	0.883	0.848
LINE	TERL	0.961 ± 0.005	0.910 ± 0.018	0.937 ± 0.013	0.969 ± 0.007	0.923 ± 0.008
	PASTEC	0.995	0.998	0.982	0.999	0.99
	Teclass	0.896	0.709	0.989	0.865	0.826
Class II	TERL	0.952 ± 0.006	0.935 ± 0.014	0.868 ± 0.028	0.980 ± 0.005	0.900 ± 0.013
	PASTEC	0.97	0.95	0.928	0.984	0.939
	Teclass	0.978	0.938	0.977	0.978	0.957
Non-TE	TERL	0.969 ± 0.002	0.921 ± 0.012	0.957 ± 0.013	0.973 ± 0.005	0.938 ± 0.004
	PASTEC	0.973	0.906	0.995	0.965	0.948
	Teclass	0.793	0.895	0.195	0.992	0.32
Macro mean	TERL	0.957 ± 0.004	0.915 ± 0.007	0.914 ± 0.007	0.972 ± 0.002	0.915 ± 0.007
	PASTEC	0.98	0.963	0.961	0.987	0.962
	Teclass	0.895	0.82	0.789	0.93	0.804

Supplementary Table S44. Performance comparison of different classification methods on dataset #2.

Class	Methods	Accuracy	precision	Recall	Specificity	F1-score
LTR	TERL	0.846 ± 0.0125	0.594 ± 0.0331	0.749 ± 0.0484	0.870 ± 0.0257	0.660 ± 0.0133
	PASTEC	0.906	0.991	0.537	0.999	0.696
	Teclass	0.796	0.491	0.542	0.859	0.515
LINE	TERL	0.895 ± 0.0050	0.819 ± 0.0366	0.614 ± 0.0530	0.965 ± 0.0115	0.699 ± 0.0269
	PASTEC	0.947	0.992	0.742	0.998	0.849
	Teclass	0.823	0.551	0.616	0.874	0.582
SINE	TERL	0.958 ± 0.0100	0.882 ± 0.0510	0.919 ± 0.0185	0.968 ± 0.0160	0.899 ± 0.0194
	PASTEC	0.953	0.987	0.775	0.997	0.868
	Teclass	0.863	0.806	0.411	0.975	0.545
Class II	TERL	0.867 ± 0.0063	0.714 ± 0.0384	0.565 ± 0.0358	0.942 ± 0.0150	0.629 ± 0.0125
	PASTEC	0.885	0.914	0.468	0.989	0.619
	Teclass	0.809	0.52	0.565	0.87	0.542
Non-TE	TERL	0.898 ± 0.0063	0.717 ± 0.0264	0.814 ± 0.0256	0.919 ± 0.0131	0.762 ± 0.0083
	PASTEC	0.716	0.413	0.996	0.646	0.584
	Teclass	0.68	0.246	0.291	0.777	0.267
Macro mean	TERL	0.893 ± 0.0026	0.745 ± 0.0088	0.732 ± 0.0066	0.933 ± 0.0017	0.739 ± 0.0061
	PASTEC	0.881	0.859	0.704	0.926	0.774
	Teclass	0.794	0.523	0.485	0.871	0.503

Supplementary Table S45. Performance comparison of different classification methods on dataset #3.

Class	Methods	Accuracy	precision	Recall	Specificity	F1-score
LTR	TERL	0.768 ± 0.0131	0.564 ± 0.0449	0.363 ± 0.0598	0.903 ± 0.0347	0.436 ± 0.0332
	PASTEC	0.861	0.981	0.454	0.997	0.621
	Teclass	0.748	0.496	0.504	0.829	0.5
LINE	TERL	0.820 ± 0.0158	0.669 ± 0.0508	0.570 ± 0.0585	0.904 ± 0.0276	0.613 ± 0.0337
	PASTEC	0.952	0.994	0.812	0.998	0.894
	Teclass	0.788	0.567	0.639	0.837	0.601
Class II	TERL	0.826 ± 0.0180	0.649 ± 0.0517	0.683 ± 0.0493	0.874 ± 0.0367	0.663 ± 0.0180
	PASTEC	0.918	0.936	0.721	0.984	0.815
	Teclass	0.839	0.67	0.698	0.885	0.684
Non-TE	TERL	0.829 ± 0.0179	0.613 ± 0.0351	0.870 ± 0.0234	0.815 ± 0.0308	0.718 ± 0.0172
	PASTEC	0.76	0.51	0.995	0.682	0.675
	Teclass	0.672	0.31	0.253	0.812	0.278
Macro mean	TERL	0.811 ± 0.0056	0.624 ± 0.0143	0.621 ± 0.0116	0.874 ± 0.0037	0.623 ± 0.0128
	PASTEC	0.873	0.855	0.746	0.915	0.797
	Teclass	0.762	0.511	0.523	0.841	0.517

Supplementary Table S46. Comparison of performance among six TE classifiers on Rice and Maize genomes.

		LTR/Copia			LTR/Gypsy			all LTR-RTs		other TEs		
Species	Methods	ST	PC	CD	ST	PC	CD	ST	PC	ST	PC	CPU/h
Rice	TEsorter (REXdb)	0.893	1.000	89.3%	0.786	1.000	78.6%	0.782	0.994	0.160	1.000	0.09
	TEsorter (GyDB)	0.843	0.993	83.0%	0.768	0.989	76.8%	0.765	0.994	NA	NA	0.15
	RepeatClassifier	0.887	0.922	NA	0.768	0.864	NA	0.773	0.908	0.396	0.881	11.3
	DeepTE	0.874	0.842	NA	0.866	0.713	NA	0.826	0.813	0.671	0.954	0.3
	TERL	0.818	0.435	NA	0.728	0.608	NA	0.729	0.522	0.186	0.828	0.03
	LTR_retriever	0.868	1.000	NA	0.830	0.979	NA	0.814	0.991	NA	NA	0.01
	LTR_classifier	0.824	1.000	NA	0.576	0.679	NA	0.645	0.822	NA	NA	1.0
Maize	TEsorter (REXdb)	0.919	0.966	91.9%	0.930	1.000	91.8%	0.793	0.998	0.329	0.997	0.1
	TEsorter (GyDB)	0.914	0.977	89.7%	0.922	0.991	90.6%	0.770	0.998	NA	NA	0.12
	RepeatClassifier	0.968	0.821	NA	0.971	0.707	NA	0.878	0.958	0.365	0.938	12.8
	DeepTE	0.914	0.790	NA	0.963	0.671	NA	0.862	0.925	0.753	0.905	0.21
	TERL	0.541	0.543	NA	0.791	0.448	NA	0.725	0.710	0.464	0.882	0.02
	LTR_retriever	0.892	0.859	NA	0.918	0.878	NA	0.757	1.000	NA	NA	0.01
	LTR_classifier	0.789	0.913	NA	0.664	0.818	NA	0.547	0.916	NA	NA	1.2

'ST' represents sensitivity, 'PC' represents precision, 'CD' represents the percentage of elements that are assigned to clades. 'CPU/h' represents the CPU time (hour). The database used in RepeatClassifier is Dfam. 'TEsorter (REXdb)' represents the tool TEsorter running based on the database REXdb. 'TEsorter (GyDB)' represents the tool TEsorter running based on the database GyDB. 'NA' represents the data that is not available.

Supplementary References

1. Richard G.F., Kerrest A. and Dujon B. (2008) Comparative genomics and molecular dynamics of DNA repeats in eukaryotes[J]. *Microbiol Mol Biol Rev*, **72(4)**, 686-727.
2. Paço A., Freitas R. and Vieira-da-Silva A. (2019) Conversion of DNA Sequences: From a Transposable Element to a Tandem Repeat or to a Gene[J]. *Genes*, **10(12)**, 1014.
3. Pray L. (2008) Transposons: The jumping genes[J]. *Nature Education*, **1(1)**, 204.
4. Cordaux R. and Batzer M.A. (2009) The impact of retrotransposons on human genome evolution[J]. *Nat Rev Genet*, **10(10)**, 691-703.
5. Copeland C.S., Brindley P.J., Heyers O. *et al.* (2003) Boudicca, a retrovirus-like long terminal repeat retrotransposon from the genome of the human blood fluke Schistosoma mansoni[J]. *J Virol*, **77(11)**, 6153-6166.
6. Muñoz-López M. and García-Pérez J.L. (2010). DNA transposons: nature and applications in genomics[J]. *Current genomics*, **11(2)**, 115–128.
7. Pace J.K. 2nd and Feschotte C. (2007) The evolutionary history of human DNA transposons: evidence for intense activity in the primate lineage[J]. *Genome Res*, **17(4)**, 422-432.
8. Kojima K.K. (2018) Human transposable elements in Repbase: genomic footprints from fish to humans[J]. *Mobile DNA*, **9**, 2.
9. Smit A.F. and Riggs A.D. (1996) Tiggers and DNA transposon fossils in the human genome[J]. *Proceedings of the National Academy of Sciences*, **93(4)**, 1443-1448.
10. Han J.S. (2010) Non-long terminal repeat (non-LTR) retrotransposons: mechanisms, recent developments, and unanswered questions[J]. *Mobile DNA*, **1**, 15.
11. Scott E.C., Gardner E.J., Masood A. *et al.* (2016) A hot L1 retrotransposon evades somatic repression and initiates human colorectal cancer[J]. *Genome Res*, **26(6)**, 745-755.
12. Miki Y., Nishisho I., Horii A. *et al.* (1992) Disruption of the APC gene by a retrotransposal insertion of L1 sequence in a colon cancer[J]. *Cancer Res*, **52(3)**, 643-645.
13. Larsen P.A., Lutz M.W., Hunnicutt K.E. *et al.* (2017) The Alu neurodegeneration hypothesis: A primate-specific mechanism for neuronal transcription noise, mitochondrial dysfunction, and manifestation of neurodegenerative disease[J]. *Alzheimers Dement*, **13(7)**, 828-838.
14. Payer L.M., Steranka J.P., Yang W.R. *et al.* (2017) Structural variants caused by Alu insertions are associated with risks for many human diseases[J]. *Proceedings of the National Academy of Sciences*, **114(20)**, E3984-E3992.
15. Gianfrancesco O., Bubb V.J. and Quinn J.P. (2017) SVA retrotransposons as potential modulators of neuropeptide gene expression[J]. *Neuropeptides*, **64**, 3-7.
16. Petrozziello T., Dios A.M., Mueller K.A. *et al.* (2020) SVA insertion in X-linked Dystonia Parkinsonism alters histone H3 acetylation associated with TAF1 gene[J]. *PLoS One*, **15(12)**, e0243655.
17. Lerat E. and Capy P. (1999). Retrotransposons and retroviruses: analysis of the envelope gene[J]. *Molecular biology and evolution*, **16(9)**, 1198-1207.
18. Havecker E.R., Gao X., Voytas D.F. (2004) The diversity of LTR retrotransposons[J]. *Genome Biol*, **5(6)**, 225.
19. Gröger V., Wieland L., Naumann M. *et al.* (2020) Formation of HERV-K and HERV-Fc1 Envelope Family Members is Suppressed on Transcriptional and Translational Level[J]. *Int J Mol Sci*, **21(21)**, 7855.
20. Nelson P.N., Hooley P., Roden D. *et al.* (2004) Human endogenous retroviruses: transposable elements with potential?[J]. *Clin Exp Immunol*, **138(1)**, 1-9.
21. Zhao J., Rycaj K., Geng S. *et al.* (2011) Expression of Human Endogenous Retrovirus Type K Envelope Protein is a Novel Candidate Prognostic Marker for Human Breast Cancer[J]. *Genes Cancer*, **2(9)**, 914-922.
22. Sawaya S., Bagshaw A., Buschiazzo E. *et al.* (2013) Microsatellite tandem repeats are abundant in human promoters and are associated with regulatory elements[J]. *PLoS One*, **8(2)**, e54710.
23. Richard G.F. and Pâques F. (2000) Mini- and microsatellite expansions: the recombination connection[J]. *EMBO Rep*, **1(2)**, 122-126.
24. Sullivan L.L., Chew K. and Sullivan B.A. (2017) α satellite DNA variation and function of the human centromere[J]. *Nucleus*, **8(4)**, 331-339.
25. Li H. (2019) Identifying centromeric satellites with dna-brnn[J]. *Bioinformatics*, **35(21)**, 4408-4410.
26. Alaguponniah S., Velayudhan Krishna D., Paul S. *et al.* (2020) Finding of novel telomeric repeats and their distribution in the human genome[J]. *Genomics*, **112(5)**, 3565-3570.
27. Riethman H. (2008) Human subtelomeric copy number variations[J]. *Cytogenet Genome Res*, **123(1-4)**, 244-252.
28. Bagshaw A.T.M. (2017) Functional Mechanisms of Microsatellite DNA in Eukaryotic Genomes[J]. *Genome Biol Evol*, **9(9)**, 2428-2443.
29. Payseur B.A., Jing P. and Haasl R.J. (2011) A genomic portrait of human microsatellite variation[J]. *Mol Biol Evol*, **28(1)**, 303-312.
30. Vergnaud G. and Denoeud F. (2000) Minisatellites: mutability and genome architecture[J]. *Genome Res*, **10(7)**, 899-907.
31. Giannuzzi G., Logsdon G.A., Chatron N. *et al.* (2021) Alpha Satellite Insertion Close to an Ancestral Centromeric Region[J]. *Mol Biol Evol*, **38(12)**, 5576-5587.
32. Hartley G. and O'Neill R.J. (2019) Centromere Repeats: Hidden Gems of the Genome[J]. *Genes (Basel)*, **10(3)**, 223.
33. Barra V. and Fachinetti D. (2018) The dark side of centromeres: types, causes and consequences of structural abnormalities implicating centromeric DNA[J]. *Nat Commun*, **9(1)**, 4340.
34. Krutilina R.I., Smirnova A.N., Mudrak O.S. *et al.*. (2003) Recognition of internal (TTAGGG)n repeats by telomeric protein TRF1 and its role in maintenance of chromosomal stability in Chinese hamster cells[J]. *Tsitologiia*, **45(12)**, 1211-1220.
35. O'Sullivan R.J. and Karlseder J. (2010) Telomeres: protecting chromosomes against genome instability[J]. *Nat Rev Mol Cell Biol*, **11(3)**, 171-181.
36. Aguilar M. and Prieto P. (2021) Telomeres and Subtelomeres Dynamics in the Context of Early Chromosome Interactions During Meiosis and Their Implications in Plant Breeding[J]. *Front Plant Sci*, **12**, 672489.
37. Jang-il S. and Jin-Wu N. (2018) The present and future of de novo whole-genome assembly[J]. *Briefings in Bioinformatics*, **19(1)**, 23–40.
38. Liao X., Li M., Zou Y. *et al.* (2019) Current challenges and solutions of de novo assembly[J]. *Quant Biol*, **7**, 90–109.
39. Liao X., Li M., Junwei L. *et al.* (2021) EPGA-SC : A Framework for de novo Assembly of Single-Cell Sequencing Reads[J]. *IEEE/ACM Transactions on Computational Biology and Bioinformatics*, **18(4)**, 1492-1503.
40. Dmitry A., Anton K., Jeffrey S.M. and Pavel A.P. (2016) HYBRIDSPADES: an algorithm for hybrid assembly of short and long reads[J]. *Bioinformatics*, **32(7)**, 1009–1015.
41. Kamath G.M., Shomorony I., Xia F., Courtade T.A. and Tse D.N. (2017) HINGE: long-read assembly achieves optimal repeat resolution[J]. *Genome Res*, **27(5)**, 747-756.
42. Miga K.H., Koren S., Rhie A. *et al.* (2020) Telomere-to-telomere assembly of a complete human X chromosome[J]. *Nature*, **585**, 79–84.
43. Narzisi G. and Schatz M.C. (2015) The challenge of small-scale repeats for indel discovery[J]. *Front Bioeng Biotechnol*, **3**, 8.

44. Lee H., Popodi E., Foster P.L. and Tang H. (2014) Detection of structural variants involving repetitive regions in the reference genome[J]. *J Comput Biol*, **21(3)**, 219-33.
45. Gao D. *et al.* (2015) Transposons play an important role in the evolution and diversification of centromeres among closely related species[J]. *Front Plant Sci*, **6**, 216.
46. Chuong E.B., Elde N.C. and Feschotte C. (2017) Regulatory activities of transposable elements: from conflicts to benefits[J]. *Nat Rev Genet*, **18(2)**, 71-86.
47. González J. *et al.* (2008) High rate of recent transposable element-induced adaptation in Drosophila melanogaster[J]. *PLoS Biol*, **6(10)**, e251.
48. Bourque G. *et al.* (2018) Ten things you should know about transposable elements[J]. *Genome Biol*, **19**, 199.
49. Ayarpadikannan S. & Kim H.S. (2014) The impact of transposable elements in genome evolution and genetic instability and their implications in various diseases[J]. *Genomics Inform*, **12(3)**, 98-104.
50. Uzunović J., Josephs E.B., Stinchcombe J.R. & Wright S.I. (2019) Transposable Elements Are Important Contributors to Standing Variation in Gene Expression in Capsella Grandiflora[J]. *Mol Biol Evol*, **36(8)**, 1734-1745.
51. Chishima T., Iwakiri J. & Hamada M. (2018) Identification of Transposable Elements Contributing to Tissue-Specific Expression of Long Non-Coding RNAs[J]. *Genes*, **9(1)**, 23.
52. Horváth V., Merenciano M. & González J. (2017) Revisiting the Relationship between Transposable Elements and the Eukaryotic Stress Response[J]. *Trends Genet*, **33(11)**, 832-841.
53. Kim Y.J., Lee J. & Han K. (2012) Transposable Elements: No More 'Junk DNA'[J]. *Genomics Inform*, **10(4)**, 226-233.
54. Lupski J.R. & Stankiewicz P. (2005) Genomic disorders: molecular mechanisms for rearrangements and conveyed phenotypes[J]. *PLoS Genet*, **1(6)**, e49.
55. Klein S.J. & O'Neill R.J. (2018) Transposable elements: genome innovation, chromosome diversity, and centromere conflict[J]. *Chromosome Res*, **26(1-2)**, 5-23.
56. Burns K. (2017) Transposable elements in cancer[J]. *Nat Rev Cancer*, **17**, 415-424.
57. Ahmadi A. *et al.* (2020) Transposable elements in brain health and disease[J]. *Ageing Res Rev*, **64**, 101153.
58. Saleh A., Macia A. & Muotri A.R. (2019) Transposable Elements, Inflammation, and Neurological Disease[J]. *Front Neurol*, **10**, 894.
59. Niu Y. *et al.* (2022) Characterizing mobile element insertions in 5675 genomes[J]. *Nucleic Acids Res*, **50(5)**, 2493-2508.
60. Huang C.R., Burns K.H. & Boeke J.D. (2012) Active transposition in genomes[J]. *Annu Rev Genet*, **46**, 651-675.
61. Cordaux R., Hedges D.J., Herke S.W. & Batzer M.A. (2006) Estimating the retrotransposition rate of human Alu elements[J]. *Gene*, **373**, 134-137.
62. Rosser J.M. & An W. (2012) L1 expression and regulation in humans and rodents[J]. *Front Biosci (Elite Ed)*, **4(6)**, 2203-2225.
63. Kannan S. *et al.* (2015) Transposable Element Insertions in Long Intergenic Non-Coding RNA Genes[J]. *Front Bioeng Biotechnol*, **3**, 71.
64. Etchegaray E., Naville M., Volff J.N. & Haftek-Terreau Z. (2021) Transposable element-derived sequences in vertebrate development[J]. *Mob DNA*, **12(1)**, 1.
65. Johnson R. and Guigó R. (2014) The RIDL hypothesis: transposable elements as functional domains of long noncoding RNAs[J]. *RNA*, **20(7)**, 959-976.
66. Hermant C. & Torres-Padilla M.E. (2021) TFs for TEs: the transcription factor repertoire of mammalian transposable elements[J]. *Genes Dev*, **35(1-2)**, 22-39.
67. Senft A.D. & Macfarlan T.S. (2021) Transposable elements shape the evolution of mammalian development[J]. *Nat Rev Genet*, **22(11)**, 691–711.
68. Usdin K. (2008) The biological effects of simple tandem repeats: lessons from the repeat expansion diseases[J]. *Genome Res*, **18(7)**, 1011-1019.
69. Haubold B. & Wiehe T. (2006) How repetitive are genomes?[J]. *BMC Bioinformatics*, **7**, 541-551.
70. Yi H. *et al.* (2014) The Tandem Repeats Enabling Reversible Switching between the Two Phases of β-Lactamase Substrate Spectrum[J]. *PLOS Genetics*, **10(9)**, e1004640.
71. Bulik-Sullivan B. *et al.* (2015) An atlas of genetic correlations across human diseases and traits[J]. *Nat Genet*, **47(11)**, 1236-1241.
72. O'Dushlaine C.T., Edwards R.J., Park S.D. & Shields D.C. (2005) Tandem repeat copy-number variation in protein-coding regions of human genes[J]. *Genome Biol*, **6(8)**, R69.
73. Hannan A.J. (2012) Tandem repeat polymorphisms: Mediators of genetic plasticity modulators of biological diversity and dynamic sources of disease susceptibility[J]. *Adv Exp Med Biol*, **769**, 1-9.
74. Fan H. & Chu J.Y. (2007) A brief review of short tandem repeat mutation[J]. *Genomics Proteomics Bioinformatics*, **5(1)**, 7-14.
75. Castillo-Lizardo M., Henneke G. & Viguera E. (2014) Replication slippage of the thermophilic DNA polymerases B and D from the Euryarchaeota Pyrococcus abyssi[J]. *Front Microbiol*, **5**, 403.
76. Gymrek M., Willems T., Reich D. & Erlich Y. (2017) Interpreting short tandem repeat variations in humans using mutational constraint[J]. *Nat Genet*, **49(10)**, 1495-1501.
77. Gemayel R., Vinces M.D., Legendre M. & Verstrepen K.J. (2010) Variable tandem repeats accelerate evolution of coding and regulatory sequences[J]. *Annu Rev Genet*, **44**, 445-477.
78. Farré M., Bosch M., López-Giráldez F., Ponsá M. & Ruiz-Herrera A. (2011) Assessing the role of tandem repeats in shaping the genomic architecture of great apes[J].*PLoS One*,**6(11)**, e27239.
79. Gemayel R., Cho J., Boeynaems S. and Verstrepen K.J. (2012) Beyond junk-variable tandem repeats as facilitators of rapid evolution of regulatory and coding sequences[J]. *Genes*, **3(3)**, 461-80.
80. Lamb J.C. & Birchler J.A. (2003) The role of DNA sequence in centromere formation[J]. *Genome Biol*, **4(5)**, 214.
81. Weider L.J., James J.E., Teresa J.C. *et al.* (2005) The functional significance of ribosomal (r) DNA variation: impacts on the evolutionary ecology of organisms[M], *Annual Review of Ecology, Evolution, and Systematics*, **2005**, 219-242.
82. Kobayashi T. (2011) Regulation of ribosomal RNA gene copy number and its role in modulating genome integrity and evolutionary adaptability in yeast[J]. *Cell Mol Life Sci*, **68(8)**, 1395-1403.
83. Raghupathy N. & Durand D. (2009) Gene cluster statistics with gene families[J]. *Mol Biol Evol*, **26(5)**, 957-968.
84. Gymrek M., Willems T., Reich D. & Erlich Y. (2017) Interpreting short tandem repeat variations in humans using mutational constraint[J]. *Nat Genet*, **49(10)**, 1495-1501.
85. Sonay T.B., Koletou M. and Wagner A. (2015) A survey of tandem repeat instabilities and associated gene expression changes in 35 colorectal cancers[J]. *BMC Genomics*, **16(1)**, 702.
86. Trost B. *et al.* (2020) Genome-wide detection of tandem DNA repeats that are expanded in autism[J]. *Nature*, **586(7827)**, 80-86.
87. Chintalaphani S.R. *et al.* (2021) An update on the neurological short tandem repeat expansion disorders and the emergence of long-read sequencing diagnostics[J]. *Acta Neuropathol Commun*, **9(1)**, 98.
88. Fotsing S.F., Margoliash J., Wang C. *et al.* (2019) The impact of short tandem repeat variation on gene expression[J]. *Nat Genet*, **51(11)**, 1652-1659.

89. Trigiante G., Blanes Ruiz N. and Cerase A. (2021) Emerging Roles of Repetitive and Repeat-Containing RNA in Nuclear and Chromatin Organization and Gene Expression[J]. *Front Cell Dev Biol*, **9**, 735527.
90. Deininger P. (2011) *Alu* elements: know the *SINEs*[J]. *Genome Biol*, **12**, 236.
91. Skipper M. (2004) *Alu* elements - a complex human affair[J]. *Nat Rev Genet*, **5**, 406.
92. Mustafina O.E. (2013) The possible roles of human *Alu* elements in aging[J]. *Front Genet*, **4**, 96.
93. Pisano M.P., Grandi N. and Tramontano E. (2021) Human Endogenous Retroviruses (HERVs) and Mammalian Apparent LTRs Retrotransposons (MaLRs) Are Dynamically Modulated in Different Stages of Immunity[J]. *Biology (Basel)*, **10(5)**, 405.
94. Ade C., Roy-Engel A.M. and Deininger P.L. (2013) *Alu* elements: an intrinsic source of human genome instability[J]. *Curr Opin Virol*, **3(6)**, 639-645.
95. Gentilini D., Mari D., Castaldi D. *et al.* (2013) Role of epigenetics in human aging and longevity: genome-wide DNA methylation profile in centenarians and centenarians' offspring[J]. *Age (Dordr)*, **35(5)**, 1961-1973.
96. Jintaridth P. and Mutirangura A. (2010) Distinctive patterns of age-dependent hypomethylation in interspersed repetitive sequences[J]. *Physiol Genomics*, **41(2)**, 194-200.
97. Ovchinnikov I., Troxel A.B. and Swergold G.D. (2001) Genomic characterization of recent human LINE-1 insertions: evidence supporting random insertion[J].*Genome Res*,**11(12)**, 2050-2058.
98. Zeggar H.R., How-Kit A., Daunay A. *et al.* (2020) Tumor DNA hypomethylation of LINE-1 is associated with low tumor grade of breast cancer in Tunisian patients[J]. *Oncol Lett*, **20(2)**, 1999-2006.
99. Estécio M.R., Gharibyan V., Shen L., *et al.* (2007) LINE-1 hypomethylation in cancer is highly variable and inversely correlated with microsatellite instability[J].*PLoS One*,**2(5)**, e399.
100. Dmitriy I.V., Natalya N.T., and Svetlana B.P. (2018) LINE-1 hypomethylation in colon cancer[J]. *Journal of Clinical Oncology*, **4_suppl**, 588-588.
101. Wolff E.M., Byun H.M., Han H.F. *et al.* (2010) Hypomethylation of a LINE-1 promoter activates an alternate transcript of the MET oncogene in bladders with cancer[J]. *PLoS Genet*, **6(4)**, e1000917.
102. Zhang X., Zhang R. and Yu J. (2020) New Understanding of the Relevant Role of LINE-1 Retrotransposition in Human Disease and Immune Modulation[J]. *Front Cell Dev Biol*, **8**, 657.
103. McKerrow W, Wang X, Mendez-Dorantes C, Mita P, Cao S, Grivainis M, Ding L, LaCava J, Burns KH, Boeke JD, Fenyö D. (2022) LINE-1 expression in cancer correlates with p53 mutation, copy number alteration, and S phase checkpoint[J]. *Proc Natl Acad Sci U S A*, **119(8)**, e2115999119.
104. Zhang R., Zhang F., Sun Z. *et al.* (2019) LINE-1 Retrotransposition Promotes the Development and Progression of Lung Squamous Cell Carcinoma by Disrupting the Tumor-Suppressor Gene FGGY[J]. *Cancer Res*, **79(17)**, 4453-4465.
105. Rodriguez-Martin B., Alvarez E.G., Baez-Ortega A. *et al.* (2020) Pan-cancer analysis of whole genomes identifies driver rearrangements promoted by LINE-1 retrotransposition[J]. *Nat Genet*, **52(3)**, 306-319.
106. Chen L., Dahlstrom J.E., Chandra A. (2012) Prognostic value of LINE-1 retrotransposon expression and its subcellular localization in breast cancer[J]. *Breast Cancer Res Treat*, **136(1)**, 129-142.
107. Wilson M., Xuya W., Carlos M.D. (2022) LINE-1 expression in cancer correlates with p53 mutation, copy number alteration, and S phase checkpoint[J]. *Proc. Natl. Acad. Sci. U.S.A.*, **119(8)**, e2115999119.
108. Ardeljan D., Taylor M.S., Ting D.T. and Burns K.H. (2017) The Human Long Interspersed Element-1 Retrotransposon: An Emerging Biomarker of Neoplasia[J]. *Clin Chem*, **63(4)**, 816-822.
109. Zhang Y., Cao L., Nguyen D. *et al.* (2016) TP53 mutations in epithelial ovarian cancer[J]. *Transl Cancer Res*, **5(6)**, 650-663.
110. Tubbs A. and Nussenzweig A. (2017) Endogenous DNA Damage as a Source of Genomic Instability in Cancer[J]. *Cell*, **168(4)**, 644-656.
111. De Luca C., Guadagni F., Sinibaldi-Vallebona P. *et al.* (2016) Enhanced expression of LINE-1-encoded ORF2 protein in early stages of colon and prostate transformation[J]. *Oncotarget*, **7(4)**, 4048-4061.
112. Quinn J.P. and Bubb V.J. SVA retrotransposons as modulators of gene expression[J]. *Mob Genet Elements*, **4**, e32102.
113. Park M.K., Lee J.C., Lee J.W., Hwang S.J. Alu cell-free DNA concentration, Alu index, and LINE-1 hypomethylation as a cancer predictor[J], *Clin Biochem*, **94**, 67-73.
114. Anastasia A.Z., Olga B., Maria V.S. *et al.* (2012) Transcriptional regulation of human-specific SVAF1 retrotransposons by cis-regulatory MAST2 sequences[J]. *Gene*, **505(1)**, 128-136.
115. Spiegel J., Adhikari S. and Balasubramanian S. (2020) The Structure and Function of DNA G-Quadruplexes[J]. *Trends Chem*, **2(2)**, 123-136.
116. Huppert J.L. and Balasubramanian S. (2007) G-quadruplexes in promoters throughout the human genome[J]. *Nucleic Acids Res*, **35(6)**, 2105.
117. Harris L.M. and Merrick C.J. (2015) G-quadruplexes in pathogens: a common route to virulence control?[J]. *PLoS Pathog*, **11(2)**, e1004562.
118. Cuevas-Diaz D.R., Wei H., Kim D.H. *et al.* (2019) Long non-coding RNAs: important regulators in the development, function and disorders of the central nervous system[J]. *Neuropathol Appl Neurobiol*, **45(6)**, 538-556.
119. Schulte A.M., Lai S., Kurtz A. *et al.* (1996) Human trophoblast and choriocarcinoma expression of the growth factor pleiotrophin attributable to germ-line insertion of an endogenous retrovirus[J]. *Proc. Natl. Acad. Sci. U.S.A.*, **93(25)**, 14759-1464.
120. Grandi N. and Tramontano E. (2018) HERV Envelope Proteins: Physiological Role and Pathogenic Potential in Cancer and Autoimmunity[J]. *Front Microbiol*, **9**, 462.
121. Mao J., Zhang Q. and Cong Y.S. (2021) Human endogenous retroviruses in development and disease[J]. *Comput Struct Biotechnol J*, **19**, 5978-5986.
122. Bilgin Sonay T., Carvalho T., Robinson M.D. *et al.* (2015) Tandem repeat variation in human and great ape populations and its impact on gene expression divergence[J]. *Genome Res*, **25(11)**, 1591-1599.
123. Bakhtiari M., Park J., Ding Y.C. *et al.* (2021) Variable number tandem repeats mediate the expression of proximal genes[J]. *Nat Commun*, **12**, 2075.
124. Aksenova A.Y. and Mirkin S.M. (2019) At the Beginning of the End and in the Middle of the Beginning: Structure and Maintenance of Telomeric DNA Repeats and Interstitial Telomeric Sequences[J]. *Genes*, **10(2)**, 118.
125. Longhese M.P. (2008) DNA damage response at functional and dysfunctional telomeres[J]. *Genes Dev*, **22(2)**, 125-140.
126. Plohl M., Me?trovi? N. and Mravinac B.(2014) Centromere identity from the DNA point of view[J].*Chromosoma*,**123(4)**,313-325.
127. Choo K.H. (2001) Domain organization at the centromere and neocentromere[J]. *Dev Cell*, **1(2)**, 165-177.
128. Willems T., Gymrek M., Highnam G. *et al.* (2014) The landscape of human STR variation[J]. *Genome Res*, **24(11)**, 1894-1904.
129. Ananda G., Walsh E., Jacob K.D. *et al.* (2013) Distinct mutational behaviors differentiate short tandem repeats from microsatellites in the human genome[J].*Genome Biol Evol*,**5(3)**,606-620.
130. Buskin A., Zhu L., Chichagova V. *et al.* (2018) Disrupted alternative splicing for genes implicated in splicing and ciliogenesis causes PRPF31 retinitis pigmentosa[J]. *Nat Commun*, **9(1)**, 4234.

131. Shang E., Cui Q., Wang X. *et al.* (2011) The bromodomain-containing gene BRD2 is regulated at transcription, splicing, and translation levels[J]. *J Cell Biochem*, **112(10)**, 2784-2793.
132. Li K., Luo H., Huang L., Luo H. and Zhu X. (2020) Microsatellite instability: a review of what the oncologist should know[J]. *Cancer Cell Int*, **20**, 16.
133. Chen W., Swanson B.J. and Frankel W.L. (2017) Molecular genetics of microsatellite-unstable colorectal cancer for pathologists[J]. *Diagn Pathol*, **12(1)**, 24.
134. Taylor J.P., Brown R.H. Jr and Cleveland D.W. (2016) Decoding ALS: from genes to mechanism[J]. *Nature*, **539(7628)**, 197-206.
135. Trost B., Engchuan W., Nguyen C.M. *et al.* (2020) Genome-wide detection of tandem DNA repeats that are expanded in autism[J]. *Nature*, **586(7827)**, 80-86.
136. Mitra I., Huang B., Mousavi N. *et al.* (2021) Patterns of de novo tandem repeat mutations and their role in autism[J]. *Nature*, **589(7841)**, 246-250.
137. Shpyleva S., Melnyk S., Pavliv O., Pogribny I., Jill James S. Overexpression of LINE-1 Retrotransposons in Autism Brain[J]. *Mol Neurobiol*, **55(2)**, 1740-1749.
138. Payer L.M., Steranka J.P., Kryatova M.S., Grillo G., Lupien M., Rocha P.P., and Burns K.H. (2021) Alu insertion variants alter gene transcript levels[J]. *Genome Res*, **31(12)**, 2236–48.
139. Lindsay M.P., Jared P.S., Daniel A. *et al.* (2019) Alu insertion variants alter mRNA splicing[J]. *Nucleic Acids Research*, **47(1)**, 421–431.
140. Pfaff A.L., Bubb V.J., Quinn J.P. *et al.* (2021) Reference SVA insertion polymorphisms are associated with Parkinson's Disease progression and differential gene expression[J]. *npj Parkinsons Dis*, **7**, 44.
141. Ko E.J., Song K.S., Ock M.S. *et al.* (2021) Expression profiles of human endogenous retrovirus (HERV)-K and HERV-R Env proteins in various cancers[J]. *BMB Rep*, **54(7)**, 368-373.
142. Fujimoto A., Fujita M., Hasegawa T. *et al.* (2020) Comprehensive analysis of indels in whole-genome microsatellite regions and microsatellite instability across 21 cancer types[J]. *Genome Res*, **30(3)**, 334–46.
143. Trost B., Engchuan W., Nguyen C.M. *et al.* (2020) Genome-wide detection of tandem DNA repeats that are expanded in autism[J]. *Nature*, **586(7827)**, 80-86.
144. Bao W., Kojima K.K. and Kohany O. (2015) Repbase Update, a database of repetitive elements in eukaryotic genomes[J]. *Mobile DNA*, **6**, 11-17.
145. Hubley R., Finn R.D., Clements J. *et al.* (2016) The Dfam database of repetitive DNA families[J]. *Nucleic Acids Res*, **44(D1)**, D81-D89.
146. Liao X, Hu K, Salhi A. *et al.* (2021) msRepDB: a comprehensive repetitive sequence database of over 80 000 species[J]. *Nucleic Acids Research*, **50(D1)**, D236-D245.
147. Paladin L., Bevilacqua M., Errigo S. *et al.* (2021) RepeatsDB in 2021: improved data and extended classification for protein tandem repeat structures[J]. *Nucleic Acids Res*, **49(D1)**, D452-D457.
148. Neumann P., Novák P., Hoštáková N. *et al.* (2019) Systematic survey of plant LTR-retrotransposons elucidates phylogenetic relationships of their polyprotein domains and provides a reference for element classification[J]. *Mobile DNA*, **10**, 1-18.
149. Jaina M., Sara C., Lowri W. *et al.* (2021) Pfam: The protein families database in 2021[J]. *Nucleic Acids Research*, **49(D1)**, D412–D419.
150. Scott M. and Thomas L.M. (2004) BLAST: at the core of a powerful and diverse set of sequence analysis tools[J]. *Nucleic Acids Research*, **32(supp_2)**, W20-5.
151. Jurka J., Klonowski P., Dagman V. and Pelton P. (1996) CENSOR-a program for identification and elimination of repetitive elements from DNA sequences. *Computers & chemistry*, **20**, 119-121.
152. Kennedy R.C., Unger M.F., Christley S. *et al.* (2011) An automated homology-based approach for identifying transposable elements[J]. *BMC Bioinformatics*, **12**, 130.
153. Li X., Kahveci T. and Settles A.M. (2007) A novel genome-scale repeat finder geared towards transposons. *Bioinformatics*, **24**, 468-476.
154. Fiston-Lavier A.S., Carrigan M., Petrov D.A. and González J. (2010) T-lex: a program for fast and accurate assessment of transposable element presence using next-generation sequencing data. *Nucleic acids research*, **39**, e36-e36.
155. Wicker T., Sabot F., Hua-Van A. *et al.* (2007) A unified classification system for eukaryotic transposable elements[J]. *Nat Rev Genet*, **8**, 973–982.
156. Ellinghaus D., Kurtz S. and Willhoeft U. (2008) LTRharvest, an efficient and flexible software for de novo detection of LTR retrotransposons[J]. *BMC bioinformatics*, **9**, 18.
157. Darzentas N., Bousios A., Apostolidou V. and Tsaftaris A.S. (2010) MASiVE: Mapping and Analysis of SireVirus Elements in plant genome sequences[J]. *Bioinformatics*, **26**, 2452-2454.
158. Rho M., Choi JH, Kim S, Lynch M. and Tang H. (2007) De novo identification of LTR retrotransposons in eukaryotic genomes[J]. *BMC Genomics*, **8**, 90.
159. Matej L., Pavel J., Ivan V., Michal C. and Eduard K. (2020) TE-greedy-nester: structure-based detection of LTR retrotransposons and their nesting[J]. *Bioinformatics*, **36(20)**, 4991–4999.
160. Wenke T., Döbel T., Sörensen T.R. *et al.* (2011) Targeted identification of short interspersed nuclear element families shows their widespread existence and extreme heterogeneity in plant genomes[J]. *Plant Cell*, **23(9)**, 3117-3128.
161. Hongliang M. and Hao W. (2017) SINE_scan: an efficient tool to discover short interspersed nuclear elements (SINEs) in large-scale genomic datasets[J]. *Bioinformatics*, **33(5)**, 743–745.
162. Yang L., Ning J. and Yanni S. (2022) AnnoSINE: a short interspersed nuclear elements annotation tool for plant genomes[J]. *Plant Physiology*, **188(2)**, 955–970.
163. Zhijian T. (2001) Eight novel families of miniature inverted repeat transposable elements in the African malaria mosquito Anopheles gambiae[J]. *Proceedings of the National Academy of Sciences*, **98**, 1699-1704.
164. Chen Y., Zhou F., Li G. and Xu Y. (2009) MUST: a system for identification of miniature inverted-repeat transposable elements and applications to Anabaena variabilis and Haloquadratum walsbyi[J]. *Gene*, **436**, 1-7.
165. Ye C., Ji G. and Liang C. (2016) detectMITE: A novel approach to detect miniature inverted repeat transposable elements in genomes[J]. *Sci Rep*, **6**, 19688.
166. Han Y. and Wessler S.R. (2010) MITE-Hunter: a program for discovering miniature inverted-repeat transposable elements from genomic sequences[J]. *Nucleic Acids Res*, **38(22)**, e199.
167. Yang G. (2013) MITE Digger, an efficient and accurate algorithm for genome wide discovery of miniature inverted repeat transposable elements[J]. *BMC Bioinformatics*, **14**, 186.
168. Crescente J.M., Zavallo D., Helguera M. and Vanzetti L.S. (2018) MITE Tracker: an accurate approach to identify miniature inverted-repeat transposable elements in large genomes[J]. *BMC Bioinformatics*, **19**, 348.
169. Lerat E. (2010) Identifying repeats and transposable elements in sequenced genomes: how to find your way through the dense forest of programs[J]. *Heredity*, **104**, 520-533.
170. Agarwal P. and States DJ. (1994) The Repeat Pattern Toolkit (RPT): analyzing the structure and evolution of the C. elegans genome[J]. *Proc Int Conf Intell Syst Mol Biol*, **2**, 1-9.
171. Gwo-Liang Chen, Yun-Juan Chang and Chun-Hway Hsueh. (2013) PRAP: an ab initio software package for automated genome-wide analysis of DNA repeats for prokaryotes[J]. *Bioinformatics*, **29**, 2683-2689.

172. Robert C.E. and Eugene W.M. (2005) PILER: identification and classification of genomic repeats[J]. *Bioinformatics*, **21**, i152-i158.
173. Nicolas J., Peterlongo P. and Tempel S. (2016) Finding and characterizing repeats in plant genomes[J]. *Plant Bioinformatics*, **1374**, 293-337.
174. Liao X., Li M., Hu K. *et al.* (2021) A sensitive repeat identification framework based on short and long reads[J]. *Nucleic Acids Research*, **49(17)**, e100-e100.
175. Ou S., Su W., Liao Y. *et al.* (2019) Benchmarking transposable element annotation methods for creation of a streamlined, comprehensive pipeline[J]. *Genome Biol*, **20**, 275.
176. Saha S., Bridges S., Magbanua Z.V. and Peterson D.G. (2008) Empirical comparison of ab initio repeat finding programs[J]. *Nucleic Acids Research*, **36**, 2284-2294.
177. Price A.L., Jones N.C. and Pevzner P.A. (2005) De novo identification of repeat families in large genomes[J]. *Bioinformatics*, **21**, i351-i358.
178. Li R., Ye J., Li S. *et al.* (2005) ReAS: Recovery of ancestral sequences for transposable elements from the unassembled reads of a whole genome shotgun[J]. *PLoS Comput Biol*, **1**, e43.
179. Shi J. and Liang C. (2019) Generic Repeat Finder: A High-Sensitivity Tool for Genome-Wide De Novo Repeat Detection[J]. *Plant Physiol*, **180**, 1803-1815.
180. Flynn J.M., Hubley R., Goubert C. *et al.* (2020) RepeatModeler2 for automated genomic discovery of transposable element families[J]. *Proceedings of the National Academy of Sciences*, **117**, 9451-9457.
181. Kurtz S., Choudhuri J.V., Ohlebusch E. *et al.* (2001) REPuter: the manifold applications of repeat analysis on a genomic scale[J]. *Nucleic Acids Res*, **29(22)**, 4633-4642.
182. Koch P., Platzer M. and Downie B.R. (2014) RepARK-de novo creation of repeat libraries from whole-genome NGS reads[J]. *Nucleic acids research*, **42**, e80-e80.
183. Chu C., Nielsen R. and Wu Y. (2016) REPdenovo: inferring de novo repeat motifs from short sequence reads[J]. *PloS one*, **11**, e0150719.
184. Liao X., Gao X., Zhang X., Wu F.X. and Wang J. (2020) RepAHR: an improved approach for de novo repeat identification by assembly of the high-frequency reads[J]. *BMC Bioinformatics*, **21(1)**, 463.
185. Guo R., Li Y.R., He S. *et al.* (2017) RepLong: de novo repeat identification using long read sequencing data[J]. *Bioinformatics*, **34**, 1099-1107.
186. M. E. J. Newman. (2006) Modularity and community structure in networks[J]. *Proceedings of the National Academy of Sciences*, **103**, 8577-8582.
187. Vincent D.B., Jean-Loup G., Renaud L. and Etienne L. (2008) Fast unfolding of communities in large networks[J]. *J. Stat. Mech. Theory Exp.*, **2008**, P10008.
188. Yang Z., Algesheimer R. and Tessone C. (2016) Comparative Analysis of Community Detection Algorithms on Artificial Networks[J]. *Scientific Reports*, **6**, 30750.
189. Abrusán G., Grundmann N., DeMester L. and Makalowski W. (2009) TEclass: a tool for automated classification of unknown eukaryotic transposable elements[J]. *Bioinformatics*, **25(10)**, 1329-1330.
190. Hirsh L., Paladin L., Piovesan D. and Tosatto S.C.E. (2018) RepeatsDB-lite: a web server for unit annotation of tandem repeat proteins[J]. *Nucleic Acids Res*, **46(W1)**, W402-W407.
191. Novák P., Neumann P. and Macas J. (2020) Global analysis of repetitive DNA from unassembled sequence reads using RepeatExplorer2. *Nat Protoc*, **15**, 3745–3776.
192. Budiš J., Kucharík M., Ďuriš F. *et al.* (2019) Dante: genotyping of known complex and expanded short tandem repeats[J]. *Bioinformatics*, **35(8)**, 1310-1317.
