# Supplementary material for: Repetitive DNA sequence detection and its role in the human genome
Source: Commun Biol. 2023 Sep 19;6:954. doi: 10.1038/s42003-023-05322-y (PMC10509279; doi:10.1038/s42003-023-05322-y)
Supplement: Supplementary file 2 — Reporting Summary [file 42003_2023_5322_MOESM2_ESM.pdf]

Corresponding author(s): Xin Gao

Last updated by author(s): Aug 29, 2023

Reporting Summary

Nature Portfolio wishes to improve the reproducibility of the work that we publish. This form provides structure for consistency and transparency in reporting. For further information on Nature Portfolio policies, see our Editorial Policies and the Editorial Policy Checklist.

Statistics

For all statistical analyses, confirm that the following items are present in the figure legend, table legend, main text, or Methods section.

n/a	Confirmed	
☐	☒	The exact sample size (*n*) for each experimental group/condition, given as a discrete number and unit of measurement
☐	☒	A statement on whether measurements were taken from distinct samples or whether the same sample was measured repeatedly
☐	☒	The statistical test(s) used AND whether they are one- or two-sided<br>*Only common tests should be described solely by name; describe more complex techniques in the Methods section.*
☒	☐	A description of all covariates tested
☐	☒	A description of any assumptions or corrections, such as tests of normality and adjustment for multiple comparisons
☐	☒	A full description of the statistical parameters including central tendency (e.g. means) or other basic estimates (e.g. regression coefficient) AND variation (e.g. standard deviation) or associated estimates of uncertainty (e.g. confidence intervals)
☐	☒	For null hypothesis testing, the test statistic (e.g. *F*, *t*, *r*) with confidence intervals, effect sizes, degrees of freedom and *P* value noted<br>*Give P values as exact values whenever suitable.*
☒	☐	For Bayesian analysis, information on the choice of priors and Markov chain Monte Carlo settings
☐	☒	For hierarchical and complex designs, identification of the appropriate level for tests and full reporting of outcomes
☐	☒	Estimates of effect sizes (e.g. Cohen's *d*, Pearson's *r*), indicating how they were calculated

Our web collection on statistics for biologists contains articles on many of the points above.

Software and code

Policy information about availability of computer code

Data collection	To evaluate the performance of each tool, we collected the reference genomes of six species: Homo sapiens, Gallus, Mouse, Drosophila melanogaster, Glycine max, and Leafcutter Ant, five groups NGS short reads, namely Leafcutter Ant, Mouse, Human-chr14 and HG003_24149_father, three groups of barcode linked reads namely HG003_24149_father, HG004_NA24143_mother and HG002_NA24385_son, three groups of CCS long reads, namely HG003_24149_father, HG004_NA24143_mother and HG002_NA24385_son , and four groups of PacBio long reads, namely dro_100k, human_100k, dmel_filtered and human polished in this study.

Data analysis

In order to comprehensively evaluate the performance of the compared methods, we used 19 evaluation metrics in this experiment, which are Num, Max(kb), N50(kb), N75(kb), N90(kb), 0 time, 1 time, >1 times, Mapping Rate(%), Reference(%), Repbase(%), Time (hour) and Memory(MB). 'Num' denotes the number of segments; 'Max(kb)' denotes the length of the larges segment; 'N50(kb)' is the length of the longest segment such that all the segments longer than this segment cover at least half (50%) of the total length of all segments; 'N75' and ' N90' are calculated in a similar way; '0 time' indicates the proportion of segments that cannot be aligned to the reference sequence in all segments; '1 time' indicates the proportion of segments that can be aligned to a unique location on the reference sequence in all segments; ' >1 times' indicates the proportion of segments that can be aligned to multiple locations on the reference sequence in all segments; 'Mapping Rate(%)' indicates the proportion of segments that can be aligned to the reference sequence in all segments; 'Reference(%)' indicates the proportion of regions marked as repetitive regions in the reference sequence that can be covered with the segments; 'Repbase(%)' indicates the proportion of fragments in Repbase that can be covered with segments; 'Annotations' indicates the total number of annotation transposable elements in the dataset; 'Predictions' indicates the number of transposable elements predicted by method; 'Sensitivity' indicates the ability to predict the true positives of each available category; 'Specificity' indicates the ability to predict the true negatives of each available category; 'PDR' indicates the false discovery rate; 'F1-score' indicates the precision and recall of a classifier into a single metric by taking their harmonic mean; 'Time (hour)' indicates the time consumption of algorithms; 'Memory(MB)' indicates the peak memory consumption of algorithms.

For manuscripts utilizing custom algorithms or software that are central to the research but not yet described in published literature, software must be made available to editors and reviewers. We strongly encourage code deposition in a community repository (e.g. GitHub). See the Nature Portfolio guidelines for submitting code & software for further information.

Data

Policy information about availability of data

All manuscripts must include a data availability statement. This statement should provide the following information, where applicable:

- Accession codes, unique identifiers, or web links for publicly available datasets
- A description of any restrictions on data availability
- For clinical datasets or third party data, please ensure that the statement adheres to our policy

The reference genomes of six species: Homo sapiens (GCF_000001405.39_GRCh38.p13_genomic_Human.fna), Gallus (GCF_016699485.2), Mouse (GCF_000001635.27), Drosophila melanogaster (GCA_018903765.1), Glycine max (GCA_000004515.5) and Leafcutter ant (GCA_000204515.1). The reference genome sequences of these six species are downloaded from the NCBI website (https://www.ncbi.nlm.nih.gov/), five groups of NGS short reads: Leafcutter Ant (ERR034186, https://www.ncbi.nlm.nih.gov/), D.melanogaster (SRR350908, https://www.ncbi.nlm.nih.gov/), Mouse (ERR2894257, https://www.ncbi.nlm.nih.gov/), Human-chr14 (https://gage.cbcb.umd.edu/) and HG003_24149_father (D2 S2 L001 R1 001, ftp://ftp-trace.ncbi.nlm.nih.gov/giab/ftp/data), three groups of barcode linked reads (HG003_24149_father, HG004_NA24143, and HG002_NA24385_son, ftp://ftp-trace.ncbi.nlm.nih.gov/giab/ftp/data) , three groups of CCS long reads (HG003_24149_father, HG004_NA24143_mother and HG002_NA24385_son, ftp://ftp-trace.ncbi.nlm.nih.gov/giab/ftp/data), and four groups of PacBio long reads (dro_100k, human_100k, dmel_filtered and human_polished, https://github.com/ruiguo-bio/replong) are used to evaluate the performance of each tool in this study.

Research involving human participants, their data, or biological material

Policy information about studies with human participants or human data. See also policy information about sex, gender (identity/presentation), and sexual orientation and race, ethnicity and racism.

Reporting on sex and gender	NA
Reporting on race, ethnicity, or other socially relevant groupings	NA
Population characteristics	NA
Recruitment	NA
Ethics oversight	NA

Note that full information on the approval of the study protocol must also be provided in the manuscript.

Field-specific reporting

Please select the one below that is the best fit for your research. If you are not sure, read the appropriate sections before making your selection.

☒ Life sciences ☐ Behavioural & social sciences ☐ Ecological, evolutionary & environmental sciences

For a reference copy of the document with all sections, see nature.com/documents/nr-reporting-summary-flat.pdf

Life sciences study design

All studies must disclose on these points even when the disclosure is negative.

Sample size

The reference sequence sizes of these six species are 3.196 Gb (H.sapiens(hg38)), 2.752 Gb(Mouse), 289 Mb(Leafcutter Ant), 168 Mb

Sample size	(D.melanogaster), 956 Mb(soybean) and 1.040 Gb(Gallus). The data sizes of five NGS datasets are 17,580,863 KB, 5,767,698 KB, 26,655,537 KB, 4,913,897 KB, 23,534,426 KB, respectively. The data sizes of four Pacbio long read datasets are 919,162 KB, 507,871 KB, 30,885,716 KB, and 109,716,724 KB, respectively.
Data exclusions	No data were excluded.
Replication	The detailed information was listed in Methods and Supplementary information.
Randomization	We conducted random splitting and reported all results in average.
Blinding	We used public data, so blinding was not relevant to our study.

Reporting for specific materials, systems and methods

We require information from authors about some types of materials, experimental systems and methods used in many studies. Here, indicate whether each material, system or method listed is relevant to your study. If you are not sure if a list item applies to your research, read the appropriate section before selecting a response.

Materials & experimental systems

n/a	Involved in the study
☒	☐ Antibodies
☒	☐ Eukaryotic cell lines
☒	☐ Palaeontology and archaeology
☒	☐ Animals and other organisms
☒	☐ Clinical data
☒	☐ Dual use research of concern
☒	☐ Plants

Methods

n/a	Involved in the study
☒	☐ ChIP-seq
☒	☐ Flow cytometry
☒	☐ MRI-based neuroimaging
